# Synthesis and Physical Properties of Tunable Aryl Alkyl Ionic Liquids (TAAILs) Comprising Imidazolium Cations Blocked with Methyl‐, Propyl‐ and Phenyl‐Groups at the C2 Position

**DOI:** 10.1002/chem.202202795

**Published:** 2022-12-12

**Authors:** Harry Biller, Thomas Strassner

**Affiliations:** ^1^ Physikalische Organische Chemie Technische Universität Dresden Bergstrasse 66 01069 Dresden Germany

**Keywords:** conductivity, electrochemical window, ionic liquids, TAAILs, viscosity

## Abstract

Imidazolium‐based ionic liquids are very popular for different applications because of their low viscosity and melting point. However, the hydrogen atom at the C2 position of the imidazolium cation can easily be deprotonated by a base, resulting in a reactive carbene. If an inert ionic liquid is needed, it is necessary to introduce an unreactive alkyl or aryl group at the C2 position to prevent deprotonation. Tunable aryl alkyl ionic liquids (TAAILs) were first introduced by our group in 2009 and are characterized by a phenyl group at the N1 position, which offers the possibility to fine‐tune the physicochemical properties by using different electron‐donating or ‐withdrawing substituents. In this work, we present a new series of TAAILs where the C2 position is blocked by a methyl, propyl or phenyl group. For each of the blocking groups, the phenyl and three different phenyl derivatives (2‐Me, 4‐OMe, 2,4‐F_2_) are compared with respect to melting point, viscosity, conductivity and electrochemical window. In addition, the differences between blocked and unblocked TAAILs with regard to their electrochemical reduction potentials are investigated by quantum chemical methods.

## Graphical abstract

**Blocking the C2 position** of imidazolium‐based tunable aryl alkyl ionic liquids (TAAILs) with methyl, propyl and phenyl groups has a strong influence on their physicochemical properties like viscosity and conductivity. Especially the electrochemical window can be enlarged. Electron‐donating and ‐withdrawing substituents on the aryl ring could further change the electron density which has also been analyzed by quantum chemical DFT calculations.

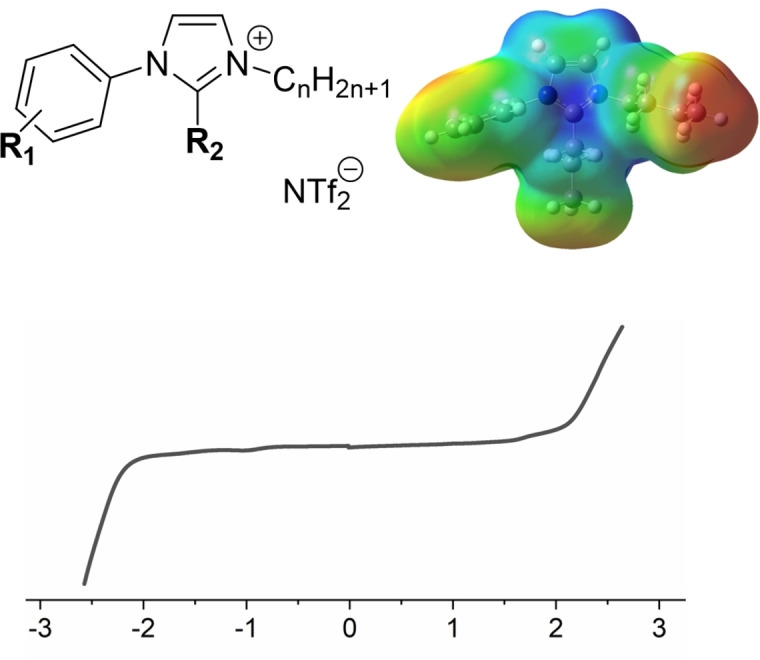

## Introduction

Ionic liquids (ILs) stand out from conventional solvents due to their interesting and unusual physical properties, and are already present in many areas of chemistry, especially in synthesis, catalysis, biochemistry and material science.[^1^, ^2^, ^3^, ^4^, ^5^, ^6^, ^7^, ^8^] Since they can be specifically customized to their fields of application due to the numerous possibilities to modify their structure, they are also referred to as green solvents or designer solvents in industry and academia.[^9^, ^10^] Ionic liquids are defined as salts with a melting point below 100 °C and consist of organic cations, such as the commonly used 1‐butyl‐3‐methylimidazolium (BMIM), and organic as well as inorganic anions, for example [Br]^−^ or bis(trifluoromethylsulfonyl)imide [NTf_2_]^−^. Although a wide variety of ILs are known, imidazolium has emerged as the most widely studied cation for ionic liquids. The advantages of this cation are generally the low melting point, low viscosity and ease of synthesis.^[11]^

Despite the advantages, there are limitations in the range of applications, especially under strongly basic conditions. The reactivity of the imidazolium cation is caused by the distinct acidity in the C2 position, which forms a carbene in the presence of a base.[^12^, ^13^] To prevent such deprotonation and the formation of a reactive carbene, it is necessary to replace the acidic proton with unreactive moieties such as alkyl or aryl groups. Yet imidazolium compounds with a methyl group at the C2 position can still be deprotonated by strong bases, forming N‐heterocyclic olefins.^[14]^ By attaching a phenyl group to the C2 position, there is no longer a proton in the beta position, but with the use of a very strong base, deprotonation in the backbone and formation of an mesoionic N‐heterocyclic carbene is still possible.[^15^, ^16^]

The addition of a substituent at the C2 position also affects the physicochemical properties significantly, even enzymatic reactions in ionic liquids could be influenced. ^[3]^ While the addition of blocking groups reduces hydrogen bonding and Coulomb attractions, the anion is restricted in its freedom of movement around the cation due to higher sterically hindrance, leading to an increase in melting point and viscosity. ^[17–20]^

Our group introduced a new type of ionic liquids by a combination of an aryl and alkyl group at the N1 and N3 of the imidazole, which allows for the preparation of a wide range of ionic liquids, the so called tuneable aryl alkyl ionic liquids (TAAILs).^[21]^ Different functional groups on the phenyl ring can be used to modify the properties of the TAAILs for various applications, such as catalysis or material synthesis.^[22]^ Here we present a series of new blocked TAAILs with a methyl, propyl and phenyl moiety at the C2 position of the imidazolium cation, which were synthesized as halide and [NTf_2_]^−^ salts. Different substituents at the aryl unit, such as the electron donating 2‐methyl and 4‐methoxy, as well as electron‐withdrawing substituents, such as the difluoro (2,4‐F_2_) are compared. For the TAAILs with [NTf_2_]^−^ anion, the viscosity, conductivity, and the electrochemical window were determined for different alkyl chain lengths.

## Results and Discussion

### Synthesis

**Figure 1:**
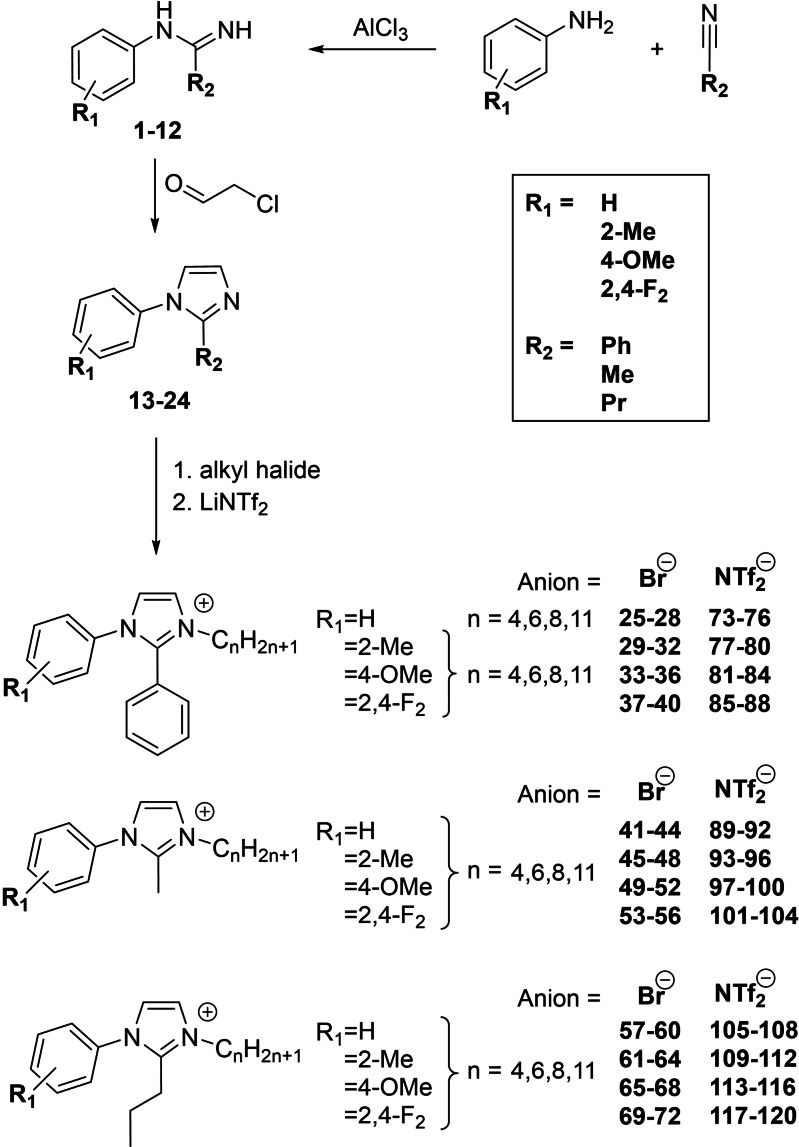
Overview of the synthetic route for compounds **1**–**120**. The functional groups R_1_ (H, 2‐methyl, 4‐methoxy, 2,4‐difluoro) and R_2_ (phenyl, methyl, propyl) are described in the legend.

The synthesis of a substituted imidazole can be accomplished by various ways which include one‐pot syntheses, cycloadditions and transition metal catalyzed coupling reactions.^[23]^ In the search for a suitable synthetic route, the focus of this work was good scalability on a multigram range, low‐cost starting materials and the possibility to introduce different substituents easily. A three‐step synthetic route was chosen, with the first step being the synthesis of an amidine (**1**–**12**) by reaction of an aniline and a nitrile in the presence of a Lewis acid such as AlCl_3_ (Figure 1).^[24]^ Since no solvent was needed for this reaction, it can be conducted at high temperatures and short reaction times can be achieved.

The desired functionality at the R^1^‐ and R^2^‐positions can be obtained by selecting the aniline and nitrile compounds accordingly. To obtain the imidazoles **13**–**24** in the second step of the synthesis, a ring closure reaction with chloroacetaldehyde was carried out. Subsequent quaternization of the imidazole with bromoalkanes of different chain lengths leads to the respective imidazolium bromide salts **25**–**72**. The final step of the synthetic route is an anion metathesis with [Li][NTf_2_] in a dichloromethane/ H_2_O two phase reaction leading to the corresponding ionic liquids **73**–**120**. All ionic compounds were dried under high vacuum at elevated temperatures to remove water residues, which has a large effect on the melting point. This was especially important for the phenyl‐blocked bromide salts, because they are strongly hygroscopic and showed good water solubility up to a chain length of 6, while the methyl‐ and propyl blocked bromide salts were poorly soluble in water.

### Thermal properties

Most compounds of the type [cation][Br] with a phenyl‐blocked cationic moiety have a melting point above 100 °C and therefore do not qualify as ionic liquids. Unfortunately, for some compounds no melting point could be determined due to a super‐cooled state, hence they are referred to as amorphous. They have a glassy solid appearance and are brittle on impact. Table 1 shows the melting points of synthesized compounds with [Br]^−^ anion in dependence of the chain length and the functionalization in the N1‐ and C2‐position.

**Table 1:** Melting points of bromide salts **25**–**72**. rt=liquid at 25 °C. amorph.=no melting point.

R_2_=phenyl				
n	Ph	(2‐Me)Ph	(4‐OMe)Ph	(2,4‐F_2_)Ph
4	143	121	amorph.	102
6	110	111	122	141
8	121	109	109	amorph.
11	126	127	amorph.	104

R_2_=methyl				
n	Ph	(2‐Me)Ph	(4‐OMe)Ph	(2,4‐F_2_)Ph
4	120	122	116	151
6	85	71	59	81
8	85	93	72	82
11	66	amorph.	78	amorph.

R_2_=propyl				
n	Ph	(2‐Me)Ph	(4‐OMe)Ph	(2,4‐F_2_)Ph
4	139	94	158	142
6	48	rt	121	114
8	88	rt	84	70
11	72	rt	92	86

The methyl‐blocked compounds with a chain length of n=4 also have a melting point above 100 °C, and therefore do not count as ILs. But as soon as the chain length increases to n=6, a significant drop in melting point is observed, ranging from 35 °C for the phenyl group(Ph) to 70 °C for the (2,4‐F_2_)Ph group, and therefore these can be classified as ILs. A significant decrease in melting points is also observed for R_2_=propyl. Here the effect of the substituents on the phenyl ring is quite strong. For R_1_=(4‐OMe)Ph and (2,4‐F_2_)Ph the melting points drop 30–35 °C per two CH_2_‐units in the alkyl chain, while in the case of 2‐MePh, the melting points for all synthesized compounds are below 100 °C and for chain lengths of n=6 and higher they are (highly viscous) room temperature ionic liquids (RTILs). Because of the methyl group present in the ortho position of the phenyl ring, steric hindrance occurs with the propyl chain, which leads to particularly bulky and crystallization‐inhibited structures as well as lower melting points.

**Figure 2:**
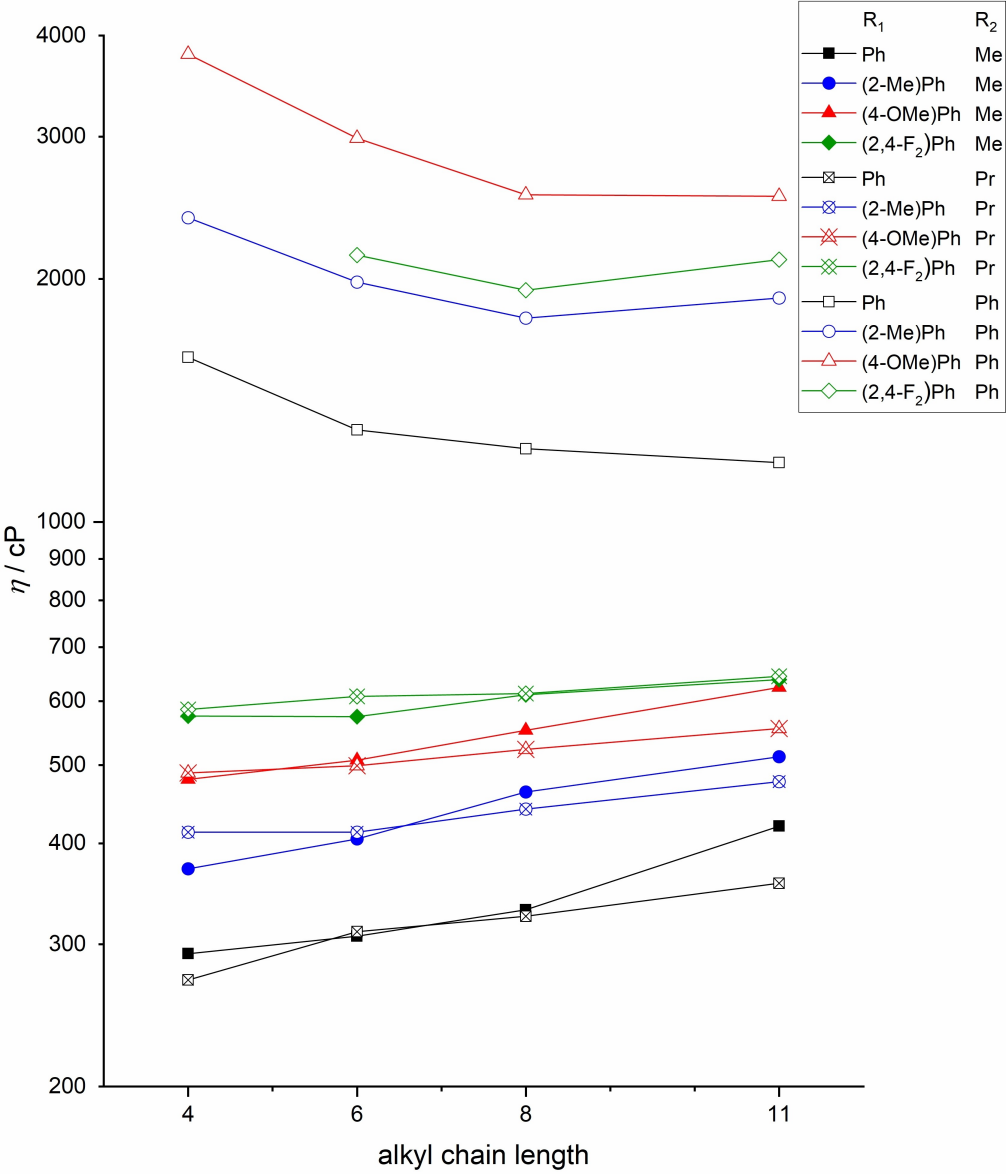
Viscosity of TAAILs **73**–**120** measured at 25 °C. Compound **85** is not displayed due to its high melting point.

The synthesized TAAILs with [NTf_2_]^−^ anions are all liquid at room temperature except for **85** with a melting point of 40 °C. Therefore, the measurement of viscosity, conductivity and electrochemical window at room temperature has not been conducted for this compound. The viscosities of the liquid [cation][NTf_2_] compounds are shown in Figure 2. The phenyl blocked compounds exhibit almost an order of magnitude higher viscosities, which can be attributed to the higher molar mass and stronger π‐π interactions of the aromatic moieties. The lowest viscosities for this type can be found for the unsubstituted Ph, followed by (2‐Me)Ph, (2,4‐F_2_)Ph and (4‐OMe)Ph.

The viscosity of the methyl‐ and propyl‐blocked TAAILs is almost an order of magnitude lower than that of the phenyl‐blocked ones and very similar for the respective substituents. Here, the (2,4‐F_2_)Ph substituent shows the highest viscosity. This indicates that the increasing viscosity in this case is not just a matter of steric hindrance, but is also influenced by the electron‐withdrawing effects of the fluorine atoms, leading to stronger π‐π and dispersion interactions.^[25]^

Table 2 shows the viscosities of the TAAILs with a butyl chain and the different substituents at the N1‐ and C2‐atoms. ^[21b]^ The unblocked ILs have the lowest viscosities, followed by the Me‐ and Pr‐blocked ILs, which have 22 % to 35 % increased viscosity and up to 50 % for the (2,4‐F_2_)Ph compounds. For the Ph‐blocked ILs, the viscosity is 7 to 10 times higher.

**Table 2:** Viscosity of different TAAILs with a butyl chain measured at 25 °C. Numbers are given in centipoise (cP).

R_1_=	R_2_=			
	H	Me	Pr	Ph
Ph	218	292	271	1599
(2‐Me)Ph	306	372	413	2380
(4‐OMe)Ph	386	480	489	3794
(2,4‐F_2_)Ph	338	575	586	solid

### Conductivity

**Figure 3:**
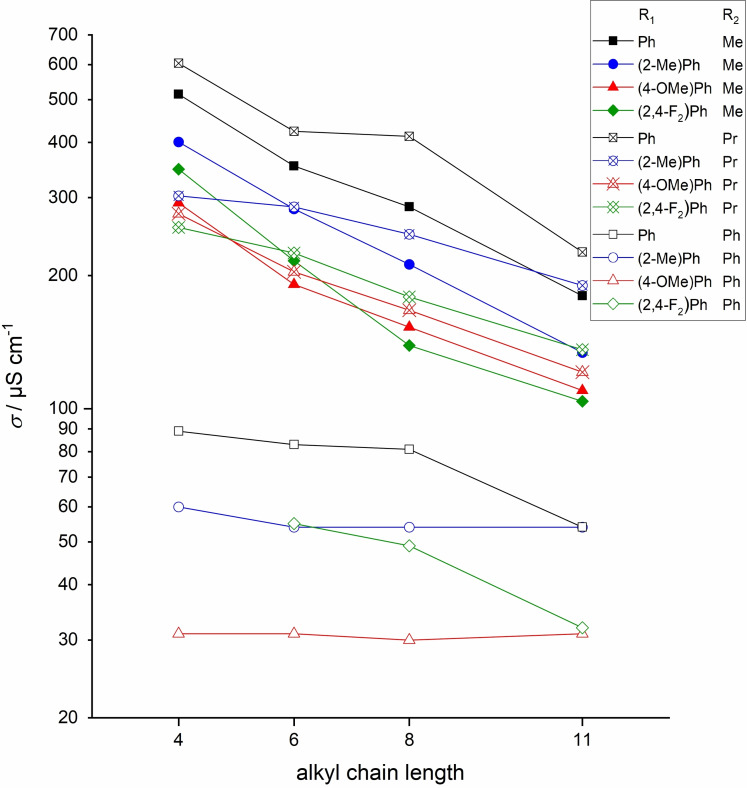
Conductivity of TAAILs **73**–**120** measured at 25 °C. Compound **85** is not displayed due to its high melting point.

The conductivity of the phenyl‐blocked TAAILs is in a much lower range of 30 to 89 μS cm^−1^, a consequence of the much higher viscosity. The conductivity of ILs **73**–**76** roughly follows their viscosity, while for the 2‐MePh and 4‐OMePh substituents, the increasing chain length and viscosity have almost no effect on the conductivity, as it increases by only 5 μS cm^−1^ and 1 μS cm^−1^, respectively. The conductivities of the liquid blocked [NTf_2_]^−^ TAAILs are shown in Figure 3.

The propyl‐blocked TAAILs show the highest conductivity for a chain length of n=6 and higher. At a chain length of 4, the methyl‐blocked (2‐Me)Ph, (2,4‐F_2_)Ph and (4‐OMe)Ph compounds exhibit a higher conductivity than the analogous propyl compounds, but the conductivity decreases significantly faster with increasing chain length. Comparing the chain lengths from n=4 to 11, the conductivity of the (2‐Me)Ph‐propyl TAAILs decreases the most.

### Electrochemical stability

**Figure 4:**
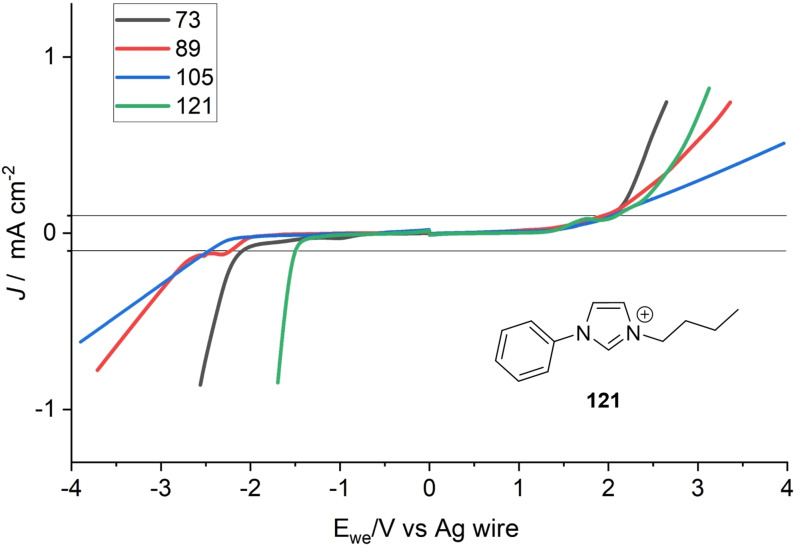
Linear sweep voltammetry of TAAILs **73**, **89**, **105** and **121**. Working electrode: glassy carbon (7.07 mm^2^); counter electrode: platinum wire; reference electrode: silver wire; scan rate: 50 mV s^−1^. Scan starts at 0 V.

The electrochemical characterization was performed using linear sweep voltammetry (LSV). A glassy carbon working electrode and a platinum wire counter electrode were used together with a silver wire as reference electrode. To compare the redox behavior of TAAILs with different blocked moieties, the LSV measurements of compounds **73**, **89**, **105** and **121** are shown in Figure 4. The lines at −0.1 and 0.1 mA cm^−2^ show the cutoff at which the cathodic limit E_red_ and anodic limit E_ox_ were determined. The electrochemical window E_EW_ is obtained from the range between E_red_ and E_ox_.

The oxidative potential E_ox_ is primarily dependent on the anion and shows a similar curve for all measured compounds. For compounds with high viscosity, it can be observed that the increase in current density is flatter once the applied voltage has exceeded the electrochemical window, which can be explained by the increased electrical resistance. The reductive potential E_red_ depends on the cation and determines at which voltage an electron is transferred. For comparison we included an IL without blocked C2 position. IL **121** is a representative of the regular TAAILs with a Ph group at N1 and a butyl chain at N3.^[26]^ With a reduction limit of −1.5 V, **121** shows much lower stability than the blocked TAAILs, which have an E_red_ greater than −2 V. On average, the blocked TAAILs have an E_red_ in the range of −2.5 V. In Table 3 all values of E_red_, E_ox_ and E_EW_ for all TAAILs are listed. The plots of all LSV measurements can be found in the Supporting Information (Figures S1‐S12).

**Table 3:** Physicochemical data of [cation][NTf_2_] ionic liquids **73**–**121**. Viscosities *η* and specific conductivity *σ* were measured at 25 °C. Anodic and cathodic cut‐off limit: 0.1 mA cm^−2^. **85** is excluded due to its high melting point.

Nr.	Cation	*η*_[_cP]	*σ* [μS cm^−1^]	E_red [_V]	E_ox_ [V]	E_EW_ [V]
**73**	PhPhC_4_Im	1599	89	−2.5	2.0	4.5
**74**	PhPhC_6_Im	1300	83	−2.5	2.1	4.6
**75**	PhPhC_8_Im	1232	81	−2.4	1.7	4.1
**76**	PhPhC_11_Im	1184	54	−2.4	2.4	4.6
**77**	(2‐MePh)PhC_4_Im	2380	60	−2.5	2.4	5.6
**78**	(2‐MePh)PhC_6_Im	1981	54	−2.3	2.9	5.2
**79**	(2‐MePh)PhC_8_Im	1788	54	−2.7	2.1	4.8
**80**	(2‐MePh)PhC_11_Im	1893	54	−2.5	2.7	5.3
**81**	(4‐OMePh)PhC_4_Im	3794	31	−2.9	1.7	4.6
**82**	(4‐OMePh)PhC_6_Im	2986	31	−2.8	2.2	5.0
**83**	(4‐OMePh)PhC_8_Im	2541	30	−2.5	1.9	4.4
**84**	(4‐OMePh)PhC_11_Im	2530	31	−3.0	2.0	5.0
**86**	(2,4‐F_2_Ph)PhC_6_Im	2139	55	−2.8	2.0	4.8
**87**	(2,4‐F_2_‐Ph)PhC_8_Im	1936	49	−2.2	2.2	4.4
**88**	(2,4‐F_2_‐Ph)PhC_11_Im	2112	32	−2.1	2.6	4.7
**89**	PhMeC_4_Im	292	514	−2.1	2.0	4.1
**90**	PhMeC_6_Im	307	354	−2.1	2.1	4.2
**91**	PhMeC_8_Im	331	286	−2.6	2.0	4.6
**92**	PhMeC_11_Im	420	180	−2.4	1.9	4.3
**93**	(2‐MePh)MeC_4_Im	372	401	−2.3	1.7	4.0
**94**	(2‐MePh)MeC_6_Im	405	283	−2.6	1.8	4.4
**95**	(2‐MePh)MeC_8_Im	463	212	−2.5	2.3	4.8
**96**	(2‐MePh)MeC_11_Im	512	134	−2.9	2.4	5.3
**97**	(4‐OMePh)MeC_4_Im	480	292	−2.2	2.0	4.2
**98**	(4‐OMePh)MeC_6_Im	507	191	−2.6	1.8	4.4
**99**	(4‐OMePh)MeC_8_Im	552	153	−2.9	1.6	4.5
**100**	(4‐OMePh)MeC_11_Im	624	110	−2.7	1.6	4.3
**101**	(2,4‐F_2_Ph)MeC_4_Im	575	348	−2.1	2.0	4.1
**102**	(2,4‐F_2_Ph)MeC_6_Im	574	216	−2.5	2.3	4.8
**103**	(2,4‐F_2_Ph)MeC_8_Im	611	139	−2.5	2.2	4.6
**104**	(2,4‐F_2_Ph)MeC_11_Im	638	104	−2.9	2.2	5.1
**105**	PhPrC_4_Im	271	604	−2.2	2.0	4.2
**106**	PhPrC_6_Im	311	424	−2.5	1.9	4.4
**107**	PhPrC_8_Im	325	413	−2.2	2.0	4.2
**108**	PhPrC_11_Im	357	226	−2.3	1.7	4.0
**109**	(2‐MePh)PrC_4_Im	413	303	−2.0	2.0	4.0
**110**	(2‐MePh)PrC_6_Im	415	286	−2.5	1.8	4.3
**111**	(2‐MePh)PrC_8_Im	441	248	−2.5	1.8	4.3
**112**	(2‐MePh)PrC_11_Im	477	190	−2.3	1.7	4.0
**113**	(4‐OMePh)PrC_4_Im	489	276	−2.5	1.6	4.1
**114**	(4‐OMePh)PrC_6_Im	499	204	−2.3	1.9	4.2
**115**	(4‐OMePh)PrC_8_Im	523	167	−2.2	1.8	4.0
**116**	(4‐OMePh)PrC_11_Im	555	121	−2.7	2.0	4.7
**117**	(2,4‐F_2_Ph)PrC_4_Im	586	257	−2.7	2.0	4.7
**118**	(2,4‐F_2_Ph)PrC_6_Im	608	225	−2.8	1.7	4.5
**119**	(2,4‐F_2_Ph)PrC_8_Im	613	179	−2.4	1.7	4.1
**120**	(2,4‐F_2_Ph)PrC_11_Im	644	136	−2.8	1.9	4.7
**121**	PhC_4_Im	218	675	−1.5	2.1	3.6

**Figure 5:**
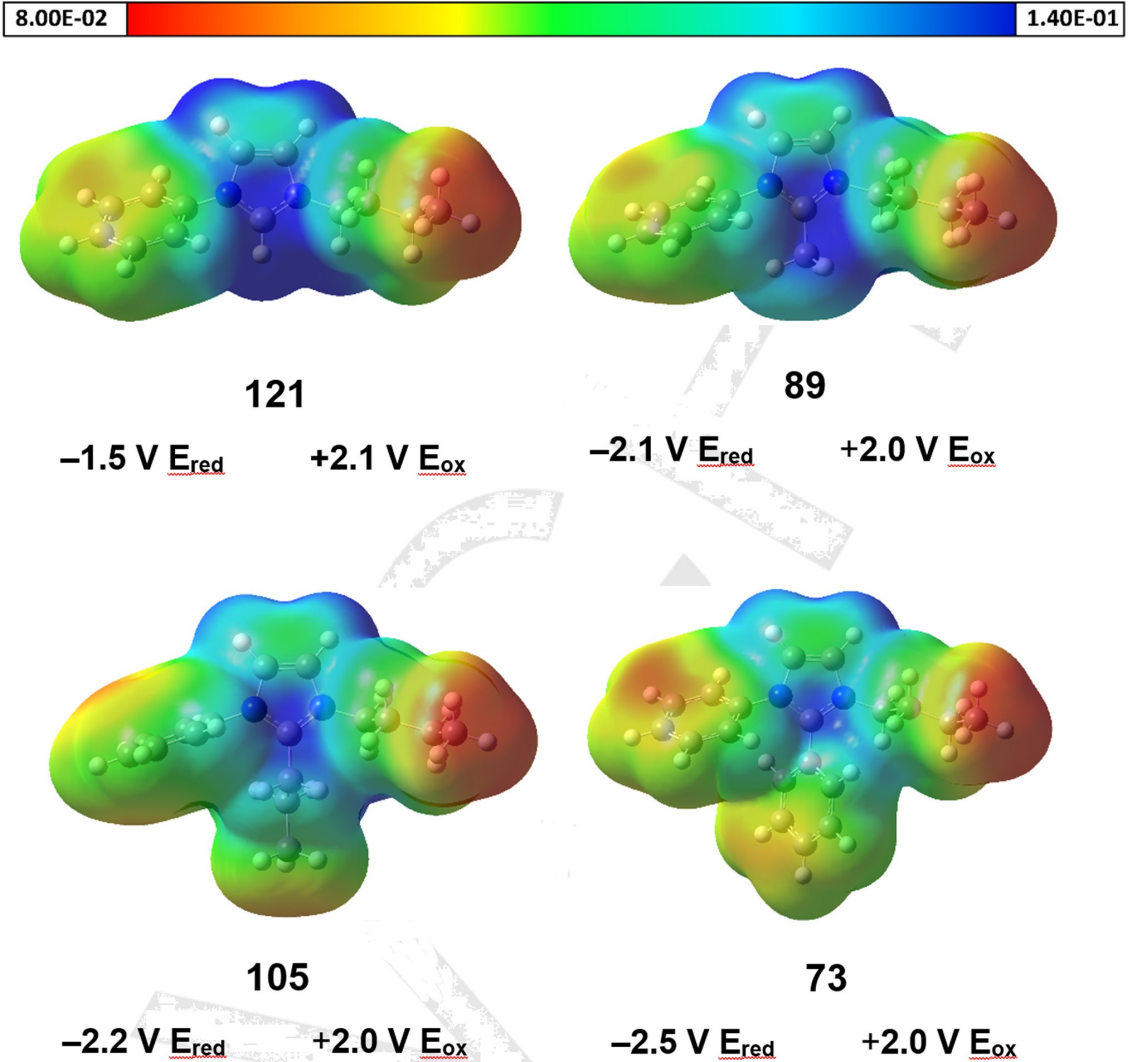
Structures of the imidazolium cations are obtained by DFT‐calculations (B3LYP/6‐311++G(d,p)). The electrostatic potential energies are indicated by color and given in Hartree atomic units.

Figure 5 shows the molecular electrostatic potential (MEP) of the imidazolium cations without the [NTf_2_]^−^ anion and the limits of the electrochemical window of the unblocked TAAIL **121** and the blocked TAAILs **73**, **89** and **105**. The structures were optimized with the Gaussian16 software package,^[27]^ using the hybrid functional Becke3LYP[^28^, ^29^, ^30^, ^31^] together with the split valence triple ζ basis set 6‐311++G(d,p)[^32^, ^33^, ^34^] and the D3 dispersion correction with the Becke–Johnson damping scheme.[^35^, ^36^] All optimized structures were confirmed to be true minima by the absence of negative frequencies after harmonic vibrational modes calculation. The molecular electrostatic potential was visualized with the GaussView6 software.

Although from the MEPs alone the observed differences of the redox properties cannot be explained, together with the experimentally determined electrochemical window, which is larger for the blocked TAAILs, they do give an indication where the charge is located. It was already reported before that the positive charge of TAAILs is mainly concentrated on the imidazole core.^[18]^ Based on the MEP of compound **121**, it can be seen that the electrostatic potential is highest at the C2 position. This location thus offers the best point of attack for a reduction, resulting in a low reduction limit of the IL. By blocking the C2 position, the shielding of the positive charge of the imidazole increases with the size of the blocking group, as indicated by the charge distribution of the MEPs. This might contribute to the increasing stability of the imidazolium core against electrochemical reduction from −1,5 V to −2.5 V.

## Conclusion

In this work, new imidazolium‐based tunable aryl alkyl ionic liquids (TAAILs) with electronically different substituents on the aryl moiety and the methyl, propyl, and phenyl blocking groups on the C2 atom of the imidazolium cation were prepared. The physicochemical properties of both the halide and the [NTf_2_]^−^ salts were investigated. It was found that the introduction of a substituent at C2 strongly influences the properties of the ionic liquids. While the increase in viscosity due to the methyl and propyl groups is only moderate, a very large increase in viscosity is observed for the phenyl‐blocked ionic liquids. The substituents on the aryl group in particular have a strong influence on the viscosity, which is due to different dispersion and π‐π interactions. When measuring the electrochemical window E_EW_, it was found that blocking the C2 position resulted in a significant increase in the reduction potential E_red_ and ionic liquids with an E_EW_ between 4 V to 5.6 V were obtained. Using quantum chemical calculations, electrostatic potential maps showed that the positive charge of the imidazolium core is better shielded by the blocking moieties, leading to an improved stability against electrochemical reduction.

## Conflict of interest

The authors declare no conflict of interest.

1

## Supporting information

As a service to our authors and readers, this journal provides supporting information supplied by the authors. Such materials are peer reviewed and may be re‐organized for online delivery, but are not copy‐edited or typeset. Technical support issues arising from supporting information (other than missing files) should be addressed to the authors.

Supporting Information

## Data Availability

The data that support the findings of this study are available in the supplementary material of this article.
