# Supplementary material for: Synthesis and Physical Properties of Tunable Aryl Alkyl Ionic Liquids (TAAILs) Comprising Imidazolium Cations Blocked with Methyl‐, Propyl‐ and Phenyl‐Groups at the C2 Position
Source: Chemistry. 2022 Dec 12;29(7):e202202795. doi: 10.1002/chem.202202795 (PMC10107658; doi:10.1002/chem.202202795)
Supplement: Supplementary file 1 — Supporting Information [file CHEM-29-0-s001.pdf]

Chemistry–A European Journal

Supporting Information

Synthesis and Physical Properties of Tunable Aryl Alkyl Ionic Liquids (TAAILs) Comprising Imidazolium Cations Blocked with Methyl-, Propyl- and Phenyl-Groups at the C2 Position

Harry Biller and Thomas Strassner*

Table of contents:

1. General information S2

2. Synthesis of amidine derivatives S3

3. Synthesis of imidazole derivatives S9

4. Synthesis of imidazolium bromides S15

5. Synthesis of NTf_2-TAAILs S39

6. Electrochemical measurements S56

7. Viscosity measurements S62

1. General informations

The solvents dichloromethane (tech. grade), isopropanol (tech. grade), toluene (tech. grade) and isohexane (tech. grade) were distilled prior to use. Diethyl ether (99 %, Merck ACS grade), ethyl acetate (99% Merck ACS grade), acetonitrile (99 %, VWR, HPLC grade), DMSO (99 %, VWR) were used as received.

Aluminum(III)chloride (granules, 99% Acros Organics), sodium hydride (60% dispersion in mineral oil, Acros Organics), aniline (99% Acros Organics), 2-methylaniline (99% Alfa Aesar), 4-methoxyaniline (99% Acros Organics), 2,4-difluoroaniline (99% ChemPur) benzonitrile (99% Acros Organics), butyronitrile (99%, Merck), chloroacetaldehyde (Merck, 50 wt% in H_2O), 1-bromobutane (98 %, abcr), 1-bromohexane (99% ChemPur) 1-bromooctane (99 %, ChemPur), 1-bromoundecane (98 %, Alfa Aesar) and [Li][NTf_2] lithium bis(trifluoromethane)sulfonimide (70% in H_2O, abcr) were used without further purification.

NMR spectroscopic data were recorded at room temperature with a Bruker AV II 300 spectrometer and a Bruker AV III 600. Chemical shifts δ are referenced to solvent signals [^{1}H NMR: $CDCl_3$ (7.26 ppm) DMSO-D_6 (2.50 ppm). ^{13}C NMR: $CDCl_3$ (77.16 ppm), DMSO-D_6 (39.52 ppm)]. Signal patterns are indicated as s, singlet; d, doublet; dd, double doublet; t, triplet; q, quartet; quint, quintet; sext, sextet; m, multiplet. Coupling constants *J* are given in Hertz (Hz).

Elemental analyses were performed with a EURO EA Element Analyzer 2010 (HEKAtech GmbH).

Viscosities were determined using a Brookfield DV2T viscosimeter with a CPA-52 rotating disc. The measurements were carried out from 20 °C to 60 °C in 5 K steps.

Conductivities were measured at 25 °C with a Mettler Toledo cond probe InLab 752-6mm connected to a Mettler Toledo SevenEasy conductivity meter.

Melting points were determined on a hotplate equipped with a microscope and digital thermometer. Samples were prepared on a microscope slide and covered with a glass slip.

Linear sweep volumetry was performed using a BioLogic SP-150 potentiostat with a glassy carbon working electrode (diameter 3mm), a Pt-wire counter electrode and an Ag-wire as pseudo reference electrode. The measurements were conducted with a sweep rate of 50 mV/s.

2. Synthesis of amidine derivatives

N-phenylbenzimidamide [1]

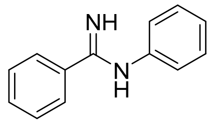

Under an argon atmosphere freshly distilled aniline (187 g, 2 mol) was mixed with benzonitrile (208 g, 2 mmol) and during 10 minutes, granulated aluminum(III)chloride (272 g, 2 mol) was added in portions with thorough stirring. The mixture was then heated at 200 °C for 4 hours. The greenish mixture, while still molten, is poured carefully into ice-cooled water. The solution was filtered, and the filtrate was poured in a steady stream into a solution of sodium hydroxide (500 mL, pH 14). The precipitate was filtered, washed with water and dried in an oven overnight. The solid was dissolved in dichloromethane and washed twice with water and brine. The organic phase was dried with $MgSO_4$ and filtered. After removing the solvent *in vacuo*, the crude product was recrystallized from toluene to afford *N*-phenylbenzimidamide as a white solid (293 g, 1.49 mol, 76 %).

^{1}H NMR (300 MHz, $CDCl_3$) δ 7.74 (d, *J* = 6.9 Hz, 2H), 7.48 – 7.31 (m, 3H), 7.27 (t, *J* = 7.8 Hz, 2H), 7.01 (t, *J* = 7.4 Hz, 1H), 4.98 (s, 2H).

^{13}C NMR (75 MHz, $CDCl_3$) δ 130.9, 129.5, 128.6, 127.0, 123.6, 122.0.

Melting point: 100°C

N-2-methylphenylbenzimidamide [2]

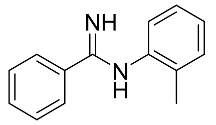

Under an argon atmosphere freshly distilled 2-methylaniline (216 g, 2 mol) was mixed with benzonitrile (208 g, 2 mmol) and during 10 minutes, granulated aluminum(III)chloride (272 g, 2 mol) was added in portions with thorough stirring. The mixture was then heated at 200 °C for 2 hours. The mixture, while still molten, is poured carefully into ice-cooled water. The solution was filtered, and the filtrate was poured in a steady stream into a solution of sodium hydroxide (500 mL, pH 14). The precipitate was filtered, washed with water and dried in an oven overnight. The solid was dissolved in and washed twice with water and brine. The organic phase was dried with $MgSO_4$ and filtered. After removing the solvent *in vacuo*, the crude product was recrystallized from toluene to afford *N*-2-methylphenylbenzimidamide as a white solid (364 g, 1.73 mol, 87 %).

^{1}H NMR (300 MHz, $CDCl_3$) δ 7.77 (d, *J* = 6.6 Hz, 2H), 7.45 – 7.23 (m, 3H), 7.20 – 7.00 (m, 2H), 6.91 (td, *J* = 7.4, 1.2 Hz, 1H), 6.77 (d, *J* = 7.7 Hz, 1H), 2.10 (s, 3H).

^{13}C NMR (75 MHz, $CDCl_3$) δ 154.3, 147.6, 135.5, 130.8, 130.6, 129.7, 128.6, 126.9, 126.9, 123.3, 121.3, 17.7.

Melting point: 95°C

N-(4-methoxyphenyl)benzimidamide [3]

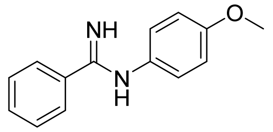

Under an argon atmosphere, sodium hydride (15 g, 750 mmol) was dissolved in dry DMSO (200 mL) and cooled externally with an ice bath. *p*-Anisidine (68.4 g, 550 mmol) and benzonitrile (52.1 g, 500 mmol) were added and the mixture was stirred at 0 °C for 60 minutes and additionally 4 h at room temperature. Ice-water (250 mL) was poured into the mixture under vigorous stirring. The solid was filtered off and dissolved in ethyl acetate. The filtrate was extracted with ethyl acetate and the combined extracts were washed with water three times, dried with $MgSO_4$ and filtered. After removing the solvent *in vacuo*, the crude product was washed with *iso*-hexane and dried under high vacuum to yield *N*-(4-methoxyphenyl)benzimidamide as a white solid (70.6 g, 312 mmol, 62 %).

^{1}H NMR (300 MHz, $CDCl_3$) δ 7.73 (d, *J* = 6.5 Hz, 2H), 7.43 – 7.22 (m, 3H), 6.82 (s, 4H), 4.85 (s, 2H), 3.71 (s, 3H).

^{13}C NMR (75 MHz, $CDCl_3$) δ 155.7, 142.1, 135.7, 130.6, 128.6, 128.5, 126.8, 122.7, 114.9, 55.5.

Melting point: 91°C

N-(2,4-difluorophenyl)benzimidamide [4]

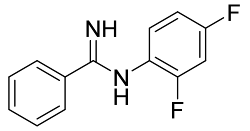

Under an argon atmosphere freshly distilled 2,4-difluoroaniline (129 g, 1 mol) was mixed with benzonitrile (104 g, 1 mmol) and during 10 minutes, granulated aluminum(III)chloride (272 g, 2 mol) was added in portions with thorough stirring. The mixture was then heated at 200 °C for 4 hours. The mixture, while still molten, is poured carefully into ice-cooled water. The solution was filtered, and the filtrate was poured in a steady stream into a solution of sodium hydroxide (500 mL, pH 14). The precipitate was filtered, washed with water and dried in an oven overnight. The solid was dissolved in dichloromethane and washed twice with water and brine. The organic phase was dried with $MgSO_4$ and filtered. After removing the solvent *in vacuo*, the crude product was recrystallized from toluene to afford *N*-(2,4-difluorophenyl)benzimidamide as an off-white solid (92.8 g, 0.4 mol, 40 %).

^{1}H NMR (300 MHz, $CDCl_3$) δ 7.77 (d, *J* = 4.4 Hz, 2H), 7.44 – 7.32 (m, 3H), 6.96 – 6.75 (m, 3H), 4.81 (s, 2H).

^{13}C NMR (75 MHz, $CDCl_3$) δ 158.7 (dd, *J* = 243.7, 11.1 Hz), 153.6 (dd, *J* = 247.5, 11.9 Hz), 135.1, 132.9 (dd, *J* = 14.8, 1.2 Hz), 130.8, 128.6, 126.9, 124.5 (dd, *J* = 9.1, 4.0 Hz), 111.5 (dd, *J* = 21.6, 3.4 Hz), 104.6 (dd, *J* = 25.6, 24.5 Hz).

Melting point: 107°C

N-phenylacetimidamide [5]

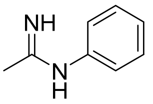

Under an argon atmosphere freshly distilled aniline (18.7 g, 200 mmol) was mixed with acetonitrile (20.8 g, 220 mmol) and cooled in an ice bath. Over the duration of 30 minutes, granulated aluminum(III)chloride (27.2 g, 200 mol) was added in portions with thorough stirring while maintaining cooling. After complete addition, the mixture was then heated to 100 °C for 1 hour. The mixture, while still molten, is poured carefully into ice-cooled water. After dissolving it was poured into a solution of sodium hydroxide (500 mL, pH 14). The solution was extracted with dichloromethane and washed twice with water and brine. The organic phase was dried with $MgSO_4$ and filtered. After removing the solvent *in vacuo*, the crude product was recrystallized from benzene to afford *N*-phenylacetimidamide as a white solid (19.9 g, 149 mol, 74 %).

^{1}H NMR (300 MHz, $CDCl_3$) δ 7.25 – 7.19 (m, 2H), 6.95 (t, *J* = 7.4 Hz, 1H), 6.79 (dd, *J* = 8.3, 1.1 Hz, 2H), 4.68 (s, 2H), 1.95 (s, 3H).

^{13}C NMR (75 MHz, $CDCl_3$) δ 149.3, 129.4, 128.9, 123.0, 122.0, 24.5.

Melting point: 49°C

N-2-methylphenylacetimidamide [6]

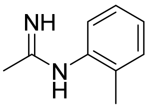

Under an argon atmosphere freshly distilled 2-methylaniline (54.1 g, 500 mmol) was mixed with acetonitrile (30.8 g, 750 mmol) and cooled in an ice bath. Over the duration of 30 minutes, granulated aluminum(III)chloride (80.0 g, 600 mol) was added in portions with thorough stirring while maintaining cooling. After complete addition, the mixture was then heated to 130 °C for 1 hour. The mixture, while still molten, is poured carefully into ice-cooled water. After dissolving it was poured into a solution of sodium hydroxide (500 mL, pH 14). The solution was extracted with dichloromethane and washed twice with water and brine. The organic phase was dried with $MgSO_4$ and filtered. After removing the solvent *in vacuo*, the crude product was overlayed with isohexane and stored at 0°C overnight. The precipitated crystals where filtered and washed with cold isohexane to afford *N*-2-methylphenylacetimidamide as a white solid (35.3 g, 238 mol, 48 %).

^{1}H NMR (300 MHz, $CDCl_3$) δ 7.23 – 7.07 (m, 2H), 7.05 – 6.96 (m, 1H), 6.78 (dd, *J* = 7.7, 1.0 Hz, 1H), 5.24 (s, 2H), 2.13 (s, 3H), 2.04 (s, 3H).

^{13}C NMR (75 MHz, $CDCl_3$) δ 146.2, 130.8, 130.4, 126.9, 123.8, 122.2, 18.1, 17.7.

N-4-methoxyphenylacetimidamide [7]

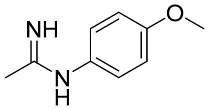

Under an argon atmosphere 4-methoxyaniline (24.9 g, 200 mmol) was mixed with acetonitrile (16.4 g, 400 mmol) and cooled in an ice bath. Over the duration of 30 minutes, granulated aluminum(III)chloride (80.0 g, 600 mol) was added in portions with thorough stirring while maintaining cooling. After complete addition, the mixture was then heated to 130 °C for 4 hours. The mixture, while still molten, is poured carefully into ice-cooled water. After dissolving it was poured into a solution of sodium hydroxide (500 mL, pH 14). The solution was extracted with ethyl acetate and washed twice with water and brine. The organic phase was dried with $MgSO_4$ and filtered. After removing the solvent *in vacuo*, the crude product was overlayed with isohexane and stored at 0°C overnight. The crude product was dissolved in water and the black residue was filtered off. The aqueous solution was extracted with ethyl acetate and washed with water and brine. The organic phase was dried with $MgSO_4$ and filtered. After removing the solvent under vacuum, the crude product was purified through column chromatography (silica, DCM/MeOH/Et_3N; 10/1/0.5) to afford *N*-4-methoxyphenylacetimidamide as a brown solid (11.9 g, 75.5 mol, 36 %).

^{1}H NMR (300 MHz, $CDCl_3$) δ 6.80 (d, *J* = 7.9 Hz, 4H), 4.78 (s, 2H), 3.75 (s, 3H), 1.99 (s, 3H).

^{13}C NMR (75 MHz, $CDCl_3$) δ 155.4, 122.7, 116.2, 114.6, 114.5, 76.5, 55.3.

Melting point: 53°C

N-(2,4-difluorophenyl)acetimidamide [8]

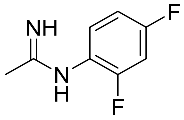

Under an argon atmosphere freshly distilled 2,4-difluoroaniline (56.2 g, 500 mmol) was mixed with acetonitrile (22.6 g, 550 mmol) and cooled in an ice bath. Over the duration of 30 minutes, granulated aluminum(III)chloride (67.3 g, 500 mol) was added in portions with thorough stirring while maintaining cooling. After complete addition, the mixture was then heated to 100 °C for 1 hour. The mixture, while still molten, is poured carefully into ice-cooled water. After dissolving it was poured into a solution of sodium hydroxide (500 mL, pH 14). The solution was extracted with dichloromethane and washed twice with water and brine. The organic phase was dried with $MgSO_4$ and filtered. After removing the solvent *in vacuo*, the crude product was overlayed with isohexane and stored at 0°C overnight. The precipitated crystals where filtered and washed with cold isohexane to afford *N*-(2,4-difluorophenyl)acetimidamide as an off-white solid (73.1 g, 430 mol, 86 %).

^{1}H NMR (300 MHz, $CDCl_3$) δ 6.95 – 6.61 (m, 3H), 4.95 (s, 2H), 1.99 (s, 3H).

^{13}C NMR (75 MHz, $CDCl_3$) δ 158.6 (dd, *J* = 243.3, 11.0 Hz), 154.1 (dd, *J* = 246.4, 12.0 Hz), 133.1, 124.9 (dd, *J* = 9.2, 3.9 Hz), 116.9 (dd, *J* = 8.9, 4.7 Hz), 111.4 (dd, *J* = 21.4, 3.4 Hz), 104.5 (t, *J* = 25.1 Hz), 77.4.

Melting point: 81°C

N-phenylbutyrimidamide [9]

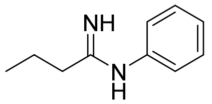

Under an argon atmosphere granulated aluminum(III)chloride (67.3 g, 500 mol) was added in portions to butyronitrile (69.1 g, 1 mol). Freshly distilled aniline (93.3 g, 1 mol) was added and the mixture was then heated to 140 °C for 1 hour. The mixture, while still molten, is poured carefully into ice-cooled water. After dissolving it was poured into a solution of sodium hydroxide (500 mL, pH 14). The solution was extracted with dichloromethane and washed twice with water and brine. The organic phase was dried with $MgSO_4$ and filtered. After removing the solvent *in vacuo*, the crude product was overlayed with isohexane and stored at 0°C overnight. The precipitated crystals where filtered and washed with cold isohexane to afford *N*-phenylbutyrimidamide as an off-white solid (47.1 g, 290 mol, 29 %).

^{1}H NMR (300 MHz, $CDCl_3$) δ 7.22 (t, *J* = 7.8 Hz, 2H), 6.94 (dd, *J* = 10.6, 4.2 Hz, 1H), 6.79 (d, *J* = 7.3 Hz, 2H), 4.48 (s, 2H), 2.19 (t, *J* = 8.0 Hz, 2H), 1.63 (sext, *J* = 7.6 Hz, 2H), 0.94 (t, *J* = 7.4 Hz, 3H).

^{13}C NMR (75 MHz, $CDCl_3$) δ 158.8, 149.4, 129.4, 122.8, 122.0, 37.6, 20.7, 13.7.

Melting point: 51°C

N-2-methylphenylbutyrimidamide [10]

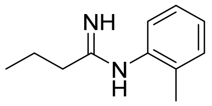

Under an argon atmosphere freshly distilled 2-methylaniline (54.1 g, 500 mmol) was mixed with butyronitrile (34.6 g, 500 mmol). Over the duration of 10 minutes, granulated aluminum(III)chloride (67.3 g, 500 mol) was added in portions with thorough stirring. After complete addition, the mixture was then heated to 150 °C for 4 hours. The mixture, while still molten, is poured carefully into ice-cooled water. After dissolving it was poured into a solution of sodium hydroxide (500 mL, pH 14). The solution was extracted with dichloromethane and washed twice with water and brine. The organic phase was dried with $MgSO_4$ and filtered. After removing the solvent *in vacuo*, the crude product was dried under high vacuum to afford *N*-phenylacetimidamide as a brown oil. (75.8 g, 430 mol, 86 %).

^{1}H NMR (300 MHz, $CDCl_3$) δ 7.14 – 7.00 (m, 2H), 6.88 (t, *J* = 7.4 Hz, 1H), 6.70 (d, *J* = 7.7 Hz, 1H), 4.52 (s, 2H), 2.22 (t, *J* = 7.6 Hz, 2H), 2.06 (s, 3H), 1.66 (sext, *J* = 7.5 Hz, 2H), 0.95 (t, *J* = 7.4 Hz, 3H).

^{13}C NMR (75 MHz, $CDCl_3$) δ 157.8, 147.6, 130.6, 129.7, 126.8, 123.0, 121.6, 37.5, 20.7, 17.6, 13.8.

Melting point: liquid at room temperature

N-4-methoxyphenylbutyrimidamide [11]

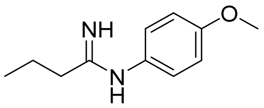

Under an argon atmosphere 4-methoxyaniline (61.6 g, 500 mmol) was mixed with butyronitrile (69.8 g, 1 mol) and cooled in an ice bath. Over the duration of 10 minutes, granulated aluminum(III)chloride (80.0 g, 600 mol) was added in portions with thorough stirring. After complete addition, the mixture was then heated at 130 °C for 1 hour. The mixture, while still molten, is poured carefully into ice-cooled water. After dissolving it was poured into a solution of sodium hydroxide (500 mL, pH 14). The solution was extracted with dichloromethane and washed twice with water and brine. The organic phase was dried with $MgSO_4$ and filtered. After removing the solvent *in vacuo*, the crude product was purified through column chromatography (silica, DCM/MeOH/Et_3N; 10/1/0.5) to afford *N*-4-methoxyphenylbutyrimidamide as a brown liquid (49.1 g, 256 mol, 51 %).

^{1}H NMR (300 MHz, $CDCl_3$) δ 6.79 (d, *J* = 7.1 Hz, 4H), 4.88 (s, 2H), 3.73 (s, 3H), 2.22 (t, *J* = 7.7 Hz, 2H), 1.66 (sext, *J* = 7.5 Hz, 2H), 0.96 (t, *J* = 7.4 Hz, 3H).

^{13}C NMR (75 MHz, $CDCl_3$) δ 178.92, 155.52, 142.05, 122.87, 114.75, 55.48, 37.62, 20.71, 13.71.

Melting point: liquid at room temperature

N- (2,4-difluorophenyl)butyrimidamide [12]

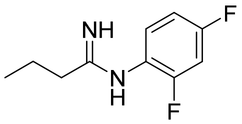

Under an argon atmosphere freshly distilled 2,4-difluoroaniline (65.2 g, 500 mol) was mixed with butyronitrile (39.4 g, 550 mmol) and cooled in an ice bath. Over the duration of 10 minutes, granulated aluminum(III)chloride (67.3 g, 500 mmol) was added and the mixture was then heated to 100 °C for 1 hour. The mixture, while still molten, is poured carefully into ice-cooled water. After dissolving it was poured into a solution of sodium hydroxide (500 mL, pH 14). The solution was extracted with dichloromethane and washed twice with water and brine. The organic phase was dried with $MgSO_4$ and filtered. After removing the solvent *in vacuo*, the crude product was overlayed with isohexane and stored at 0°C overnight. The precipitated crystals where filtered and washed with cold isohexane to afford *N*-(2,4-difluorophenyl)butyrimidamide as an off-white solid (78.2 g, 394 mol, 79 %).

^{1}H NMR (300 MHz, $CDCl_3$) δ 6.97 – 6.75 (m, 3H), 4.86 (s, 2H), 2.29 (t, *J* = 7.5 Hz, 2H), 1.70 (sext, *J* = 7.2 Hz, 2H), 1.00 (t, *J* = 7.3 Hz, 3H).

^{13}C NMR (75 MHz, $CDCl_3$) δ 161.0, 158.7 (dd, *J* = 243.7, 10.9 Hz), 154.0 (dd, *J* = 247.2, 12.1 Hz), 132.2 (dd, *J* = 13.8, 2.3 Hz), 124.9 (dd, *J* = 8.9, 3.4 Hz), 111.5 (dd, *J* = 21.7, 3.8 Hz), 104.5 (dd, *J* = 25.9, 24.4 Hz), 37.3, 20.5, 13.5.

Melting point: 62°C

3. Synthesis of imidazole derivatives

1,2-diphenyl-1*H*-imidazole [13]

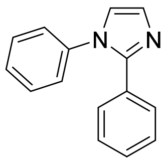

N-Phenylbenzamidine **1** (292 g, 1.49 mol), chloroacetaldehyde (467 g, 2.98 mol, 50 wt% in H_2O) and $NaHCO_3$ (125 g, 1.49 mmol) were put into 2-propanol (3 L) and refluxed for 16 h. The solvent was partially removed *in vacuo* and H_2O (150 mL) was added to the residue. The aqueous phase was extracted twice with dichloromethane and the combined organic phases were washed with water, diluted $NaHCO_3$ (5 %) and brine. The solution was dried with $MgSO_4$, filtered and concentrated under vacuum. The black solution was then added to an excess of diethyl ether, which immediately precipitated a black solid. The precipitate was filtered off and the filtrate was concentrated *in vacuo*. Precipitation in *iso*-hexane gave 1,2-diphenyl-1*H*-imidazole as a white solid (248 g, 1.08 mol, 72 %).

^{1}H NMR (300 MHz, $CDCl_3$) δ 7.34 – 7.29 (m, 5H), 7.22 – 7.13 (m, 6H), 7.08 (d, *J* = 1.3 Hz, 1H).

^{13}C NMR (75 MHz, $CDCl_3$) δ 146.6, 138.5, 130.0, 129.5, 128.8, 128.6, 128.5, 128.3, 128.2, 125.9, 122.9.

Melting point: 76°C

1-(2-Methylphenyl)-2-phenyl-1*H*-imidazole [14]

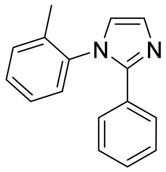

N-2-Methylphenylbenzamidine **2** (127 g, 604 mmol), chloroacetaldehyde (142 g, 906 mmol, 50 wt% in H_2O) and $NaHCO_3$ (76 g, 906 mmol) were put into 2-propanol (500 mL) and refluxed for 16 h. The solvent was partially removed *in vacuo* and H_2O (150 mL) was added to the residue. The aqueous phase was extracted twice with dichloromethane and the combined organic phases were washed with water, diluted $NaHCO_3$ (5 %) and brine. The solution was dried with $MgSO_4$, filtered and concentrated under vacuum. The black solution was then added to an excess of diethyl ether, which immediately precipitated a black solid. The precipitate was filtered off and the filtrate was concentrated *in vacuo*. Precipitation in *iso*-hexane gave 1-(2-methylphenyl)-2-phenyl-1*H*-imidazole as a yellow solid (104 g, 442 mmol, 73 %).

^{1}H NMR (300 MHz, $CDCl_3$) δ 7.33 – 7.10 (m, 10H), 6.94 (d, *J* = 1.2 Hz, 1H), 1.86 (s, 3H).

^{13}C NMR (75 MHz, $CDCl_3$) δ 146.8, 137.8, 135.0, 131.2, 130.4, 129.0, 128.9, 128.2, 128.1, 127.4, 127.4, 126.9, 122.7, 17.3.

Melting point: 98°C

1-(4-methoxyphenyl)-2-phenyl-1H-imidazole [15]

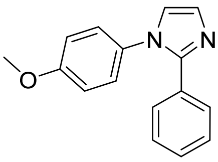

N-(4-Methoxyphenyl)benzamidine **3** (70.6 g, 312 mmol), chloroacetaldehyde (98 g, 624 mmol, 50 wt% in H_2O) and $NaHCO_3$ (52.4 g, 642 mmol) were put into 2-propanol (300 mL) and refluxed for 16 h. The solvent was partially removed *in vacuo* and H_2O (150 mL) was added to the residue. The aqueous phase was extracted twice with dichloromethane and the combined organic phases were washed with water, diluted $NaHCO_3$ (5 %) and brine. The solution was dried with $MgSO_4$, filtered and concentrated under vacuum. The black solution was then added to an excess of diethyl ether, which immediately precipitated a black solid. The precipitate was filtered off and the filtrate was concentrated *in vacuo*. Precipitation in *iso*-hexane gave 1-(4-methoxyphenyl)-2-phenyl-1*H*-imidazole as a yellow solid (58 g, 232 mmol, 74 %).

^{1}H NMR (300 MHz, $CDCl_3$) δ 7.39 – 7.29 (m, 2H), 7.23 – 7.13 (m, 4H), 7.07 (d, *J* = 8.9 Hz, 2H), 7.03 (d, *J* = 1.3 Hz, 1H), 6.83 (d, *J* = 8.9 Hz, 2H), 3.76 (s, 3H).

^{13}C NMR (75 MHz, $CDCl_3$) δ 159.2, 146.6, 131.4, 130.1, 128.5, 128.4, 128.2, 128.1, 127.0, 123.1, 114.5, 55.4.

Melting point: 87°C

1-(2,4-difluorophenyl)-2-phenyl-1*H*-imidazole [16]

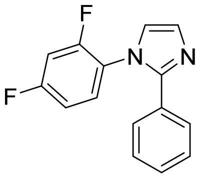

N-(2,4-difluorophenyl)benzimidamide **4** (67.3 g, 290 mmol), chloroacetaldehyde (68.3 g, 435 mmol, 50 wt% in H_2O) and $NaHCO_3$ (36.5 g, 435 mmol) were put into 2-propanol (300 mL) and refluxed for 16 h. The solvent was partially removed *in vacuo* and H_2O (150 mL) was added to the residue. The aqueous phase was extracted twice with dichloromethane and the combined organic phases were washed with water, diluted $NaHCO_3$ (5 %) and brine. The solution was dried with $MgSO_4$, filtered and concentrated under vacuum. The black solution was then added to an excess of diethyl ether, which immediately precipitated a black solid. The precipitate was filtered off and the filtrate was concentrated *in vacuo*. Precipitation in *iso*-hexane gave 1-(2,4-difluorophenyl)-2-phenyl-1*H*-imidazole as a yellow solid (47.1 g, 184 mmol, 63 %).

^{1}H NMR (300 MHz, $CDCl_3$) δ 7.37 – 7.26 (m, 2H), 7.26 – 7.07 (m, 5H), 7.02 (s, 1H), 6.95 – 6.78 (m, 2H).

^{13}C NMR (75 MHz, $CDCl_3$) δ 162.4 (dd, *J* = 252.3, 10.9 Hz), 156.9 (dd, *J* = 254.9, 12.5 Hz), 147.6, 129.8, 129.6 (dd, *J* = 10.0, 1.3 Hz), 129.2, 128.8, 128.4, 128.0, 123.02. 112.1 (dd, *J* = 22.5, 4.0 Hz), 105.5 (dd, *J* = 26.4, 23.4 Hz).

Melting point: 99°C

2-methyl-1-phenyl-1*H*-imidazole [17]

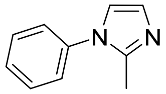

N-phenylacetimidamide **5** (80.5 g, 600 mmol) and chloroacetaldehyde (113 g, 720 mmol, 50 wt% in H_2O) were dissolved in toluene and stirred at 80 °C for 4 h. NaOH solution was added until the aqueous phase was basic. The reaction was continued for 4 h under reflux. After the solution had cooled down, the phases were separated. The aqueous phase was extracted with toluene and the combined organic phases were concentrated *in vacuo*. The solution was poured into an excess of diethyl ether and the formed black precipitate was filtered off. The filtrate was washed with water and brine and dried with $MgSO_4$. The solvent was evaporated *in vacuo* and the crude product was purified through distillation to give 2-methyl-1-phenyl-1*H*-imidazole as a yellow liquid (55.5 g, 351 mmol, 59 %).

^{1}H NMR (300 MHz, $CDCl_3$) δ 7.47 – 7.26 (m, 3H), 7.26 – 7.16 (m, 2H), 6.94 (dd, *J* = 7.9, 1.4 Hz, 2H), 2.29 (s, 3H).

^{13}C NMR (75 MHz, $CDCl_3$) δ 144.4, 137.8, 129.3, 128.0, 127.5, 125.3, 120.5, 13.6.

Melting point: liquid at room temperature

1-(2-methylphenyl)-2-methyl-1*H*-imidazole [18]

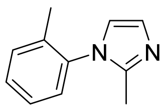

N-2-methylphenylacetimidamide **6** (34.5 g, 233 mmol) and chloroacetaldehyde (43.9 g, 280 mmol, 50 wt% in H_2O) were dissolved in toluene and stirred at 90 °C for 16 h. NaOH solution was added until the aqueous phase was basic. The reaction was continued for 4 h under reflux. After the solution had cooled down, the phases were separated. The aqueous phase was extracted with toluene and the combined organic phases were concentrated *in vacuo*. The solution was poured into an excess of diethyl ether and the formed black precipitate was filtered off. The filtrate was washed with water and brine and dried with $MgSO_4$. The solvent was evaporated *in vacuo* and the crude product was purified through distillation to yield 1-(2-methylphenyl)-2-methyl-1*H*-imidazole as a yellow liquid (16.4 g, 95.5 mmol, 41 %)

^{1}H NMR (500 MHz, $CDCl_3$) δ 7.35 – 7.23 (m, 3H), 7.13 (d, *J* = 7.7 Hz, 1H), 7.01 (d, *J* = 1.3 Hz, 1H), 6.83 (d, *J* = 1.3 Hz, 1H), 2.14 (s, 3H), 2.00 (s, 3H).

^{13}C NMR (75 MHz, $CDCl_3$) δ 145.1, 136.8, 135.3, 131.1, 129.1, 127.5, 127.4, 126.9, 120.4, 17.2, 13.0.

Melting point: liquid at room temperature

1-(4-methoxyphenyl)-2-methyl-1*H*-imidazole [19]

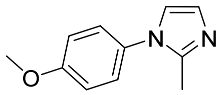

N-4-methoxyphenylacetimidamide **7** (25.4 g, 155 mmol) and chloroacetaldehyde (36.4 g, 232 mmol, 50 wt% in H_2O) were dissolved in toluene and stirred at 90 °C for 16 h. NaOH solution was added until the aqueous phase was basic. The reaction was continued for 4 h under reflux. After the solution had cooled down, the phases were separated. The aqueous phase was extracted with toluene and the combined organic phases were concentrated *in vacuo*. The solution was poured into an excess of diethyl ether and the formed black precipitate was filtered off. The filtrate was washed with water and brine and dried with $MgSO_4$. The solvent was evaporated *in vacuo* and the crude product was purified through column chromatography (silica, dichloromethane/MeOH; 20/1) to yield 1-(4-methoxyphenyl)-2-methyl-1*H*-imidazole as a brown solid (20.2 g, 107 mmol, 69 %)

^{1}H NMR (300 MHz, $CDCl_3$) δ 7.17 (d, *J* = 9.0 Hz, 2H), 7.02 – 6.87 (m, 4H), 3.83 (s, 3H), 2.30 (s, 3H).

^{13}C NMR (75 MHz, $CDCl_3$) δ 159.4, 145.0, 130.9, 127.3, 126.9, 121.0, 114.6, 55.6, 13.6.

Melting point: 49°C

1-(2,4-difluorophenyl)-2-methyl-1*H*-imidazole [20]

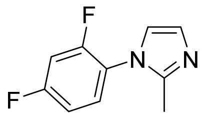

N-(2,4-difluorophenyl)acetimidamide **8** (68.1 g, 400 mmol) and chloroacetaldehyde (75.4 g, 480 mmol, 50 wt% in H_2O) were dissolved in toluene and stirred at 80 °C for 4 h. NaOH solution was added until the aqueous phase was basic. The reaction was continued for 4 h under reflux. After the solution had cooled down, the phases were separated. The aqueous phase was extracted with toluene and the combined organic phases were concentrated *in vacuo*. The solution was poured into an excess of diethyl ether and the formed black precipitate was filtered off. The filtrate was washed with water and brine and dried with $MgSO_4$. The solvent was evaporated *in vacuo* and the crude product was purified through distillation to yield 1-(2,4-difluorophenyl)-2-methyl-1*H*-imidazole as a white solid (41.4 g, 211 mmol, 53 %)

^{1}H NMR (300 MHz, $CDCl_3$) δ 7.23 (td, *J* = 8.8, 5.8 Hz, 1H), 7.01 – 6.89 (m, 3H), 6.86 (s, 1H), 2.21 (d, *J* = 0.9 Hz, 3H).

^{13}C NMR (75 MHz, $CDCl_3$) δ 162.6 (dd, *J* = 252.1, 11.0 Hz), 157.1 (dd, *J* = 254.6, 12.5 Hz), 145.7, 129.4 (dd, *J* = 10.1, 1.4 Hz), 128.0, 122.1 (dd, *J* = 13.0, 4.1 Hz), 121.04, 112.2 (dd, *J* = 22.5, 4.0 Hz), 105.4 (dd, *J* = 26.4, 23.6 Hz), 13.1 (d, *J* = 2.3 Hz).

Melting point: 40°C

1-phenyl-2-propyl-1*H*-imidazole [21]

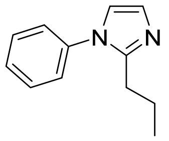

N-phenylbutyrimidamide **9** (32.4 g, 200 mmol), chloroacetaldehyde 375.7 g, 240 mmol, 50 wt% in H_2O) and $NaHCO_3$ (16.0 g, 400 mmol) were put into 2-propanol (300 mL) and refluxed for 16 h. The solvent was partially removed *in vacuo* and the residue was dissolved in dichloromethane (150 mL). The aqueous phase was extracted twice with dichloromethane and the combined organic phases were washed with water, diluted $NaHCO_3$ (5 %) and brine. The solution was dried with $MgSO_4$, filtered and concentrated under vacuum. The black solution was then added to an excess of diethyl ether, which immediately precipitated a black solid. The precipitate was filtered off and the filtrate was concentrated *in vacuo*. The crude product was purified through distillation to yield 1-phenyl-2-propyl-1*H*-imidazole as a yellow liquid (29.1 g, 156 mol, 78 %).

^{1}H NMR (300 MHz, $CDCl_3$) δ 7.47 – 7.30 (m, 3H), 7.20 (d, *J* = 8.0 Hz, 2H), 6.98 (t, *J* = 1.1 Hz, 1H), 6.89 (t, *J* = 1.2 Hz, 1H), 2.53 (t, *J* = 7.5 Hz, 2H), 1.62 (sext, *J* = 7.5 Hz, 2H), 0.81 (t, *J* = 7.4 Hz, 3H).

^{13}C NMR (75 MHz, $CDCl_3$) δ 148.6, 138.1, 129.5, 128.3, 127.7, 126.0, 120.6, 29.1, 21.5, 13.9.

Melting point: liquid at room temperature

1-(2-methylphenyl)-2-propyl-1*H*-imidazole [22]

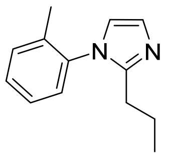

N-2-methylphenylbutyrimidamide **10** (68.7 g, 399 mmol) and chloroacetaldehyde (73.5 g, 468 mmol, 50 wt% in H_2O) were dissolved in toluene and stirred at 80 °C for 16 h. NaOH solution was added until the aqueous phase was basic. The reaction was continued for 4 h under reflux. After the solution had cooled down, the phases were separated. The aqueous phase was extracted with toluene and the combined organic phases were concentrated *in vacuo*. The solution was poured into an excess of diethyl ether and the formed black precipitate was filtered off. The filtrate was washed with water and brine and dried with $MgSO_4$. The solvent was evaporated *in vacuo* and the crude product was purified through distillation to give 1-(2-methylphenyl)-2-propyl-1*H*-imidazole as a yellow liquid (53.4 g, 266 mmol, 68 %).

^{1}H NMR (500 MHz, $CDCl_3$) δ 7.35 – 7.24 (m, 3H), 7.14 (d, *J* = 7.7 Hz, 1H), 7.05 (s, 1H), 6.82 (s, 1H), 2.38 (t, *J* = 7.8 Hz, 2H), 2.01 (s, 3H), 1.62 (sext, *J* = 7.5 Hz, 2H), 0.83 (t, *J* = 7.4 Hz, 3H).

^{13}C NMR (75 MHz, $CDCl_3$) δ 148.7, 136.8, 135.4, 131.0, 129.0, 127.7, 127.6, 126.7, 120.2, 28.7, 21.2, 17.2, 13.8.

Melting point: liquid at room temperature

1-(4-methoxyphenyl)-2-propyl-1*H*-imidazole [23]

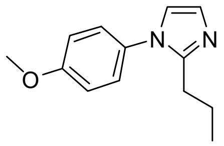

N-4-methoxyphenylbutyrimidamide **11** (42.3 g, 220 mmol) and chloroacetaldehyde (41.4 g, 264 mmol, 50 wt% in H_2O) were dissolved in toluene and stirred at 80 °C for 16 h. NaOH solution was added until the aqueous phase was basic. The reaction was continued for 4 h under reflux. After the solution had cooled down, the phases were separated. The aqueous phase was extracted with toluene and the combined organic phases were concentrated *in vacuo*. The solution was poured into an excess of diethyl ether and the formed black precipitate was filtered off. The filtrate was washed with water and brine and dried with $MgSO_4$. The solvent was evaporated *in vacuo* and the crude product was purified through distillation to give 1-(4-methoxyphenyl)-2-propyl-1*H*-imidazole as a yellow liquid (17.8 g, 82.4 mmol, 38 %).

^{1}H NMR (300 MHz, $CDCl_3$) δ 7.17 – 7.04 (m, 2H), 7.01 – 6.78 (m, 4H), 3.78 (s, 3H), 2.50 (t, *J* = 7.7 Hz, 2H), 1.60 (sext, *J* = 7.6 Hz, 2H), 0.80 (t, *J* = 7.4 Hz, 3H).

^{13}C NMR (75 MHz, $CDCl_3$) δ 159.4, 148.8, 130.7, 127.2, 121.0, 114.5, 55.5, 28.9, 21.5, 13.8.

Melting point: liquid at room temperature

1-(2,4-difluorophenyl)-2-propyl-1*H*-imidazole [24]

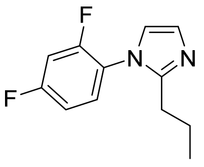

N-(2,4-difluorophenyl)butyrimidamide **12** (65.4 g, 330 mmol) and chloroacetaldehyde (62.2 g, 396 mmol 50 wt% in H_2O) were dissolved in toluene and stirred at 80 °C for 4 h. NaOH solution was added until the aqueous phase was basic. The reaction was continued for 4 h under reflux. After the solution had cooled down, the phases were separated. The aqueous phase was extracted with toluene and the combined organic phases were concentrated *in vacuo*. The solution was poured into an excess of diethyl ether and the formed black precipitate was filtered off. The filtrate was washed with water and brine and dried with $MgSO_4$. The solvent was evaporated *in vacuo* and the crude product was purified through distillation to yield 1-(2,4-difluorophenyl)-2-propyl-1*H*-imidazole as a white solid (46.9 g, 211 mmol, 64 %).

^{1}H NMR (300 MHz, $CDCl_3$) δ 7.30 – 7.16 (m, 1H), 7.02 (s, 1H), 7.00 – 6.85 (m, 2H), 6.84 (s, 1H), 2.43 (t, *J* = 7.6 Hz, 2H), 1.62 (sext, *J* = 7.6 Hz, 2H), 0.81 (t, *J* = 7.4 Hz, 3H).

^{13}C NMR (75 MHz, $CDCl_3$) δ 162.61 (dd, *J* = 252.1, 10.9 Hz), 157.34 (dd, *J* = 254.5, 12.5 Hz), 149.39, 129.73 (dd, *J* = 10.0, 1.3 Hz), 128.05, 122.06 (dd, *J* = 13.1, 4.1 Hz), 120.85, 112.08 (dd, *J* = 22.5, 4.0 Hz), 105.35 (dd, *J* = 26.4, 23.6 Hz), 28.67, 21.14, 13.77.

Melting point: 32 °C

4. Synthesis of imidazolium bromides

3-butyl-1,2-diphenyl-1*H*-imidazol-3-ium bromide [25]

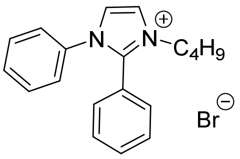

In an Ace pressure tube 1,2-diphenyl-1*H*-imidazole **13** (22 g, 100 mmol) and 1-brombutane (15.2 g, 110 mmol) were dissolved in acetonitrile (30 mL). The reaction was stirred for 72 h at 90 °C. When the reaction was complete, the solvent was evaporated and the residue dissolved in a small amount of DCM. The solution was then poured into a large excess of diethyl ether. The precipitate formed was filtered off and washed with diethyl ether. After drying *in vacuo* the product was obtained as a white solid (22.9 g, 64.1 mmol, 64 %).

^{1}H NMR (300 MHz, $CDCl_3$) δ 8.19 (d, *J* = 2.2 Hz, 1H), 7.69 (d, *J* = 2.1 Hz, 1H), 7.55 – 7.30 (m, 10H), 4.23 (t, *J* = 7.5 Hz, 2H), 1.78 (quint, *J* = 7.5 Hz, 2H), 1.22 (sext, *J* = 7.5 Hz, 2H), 0.75 (t, *J* = 7.3 Hz, 3H).

^{13}C NMR (75 MHz, $CDCl_3$) δ 144.1, 134.7, 132.3, 130.8, 130.3, 129.8, 129.5, 126.0, 123.6, 123.4, 121.1, 49.22, 31.7, 19.4, 13.2.

Melting point: 143°C

Elemental analysis $C_{19}H_{21}BrN_2$ calc.: C: 63.87 %, H: 5.92 %, N: 7.84 %.
found: C: 63.87 %, H: 5.97 %, N: 7.83 %.

3-hexyl-1,2-diphenyl-1*H*-imidazol-3-ium bromide [26]

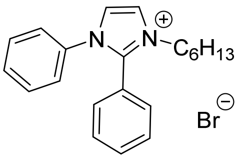

In an Ace pressure tube 1,2-diphenyl-1*H*-imidazole **13** (5.86 g, 26.6 mmol) and 1-bromohexane (4.9 g, 29.3 mmol) were dissolved in acetonitrile (5 mL). The reaction was stirred for 72 h at 90 °C. When the reaction was complete, the solvent was evaporated and the residue dissolved in a small amount of DCM. The solution was then poured into a large excess of diethyl ether. The precipitate formed was filtered off and washed with diethyl ether. After drying *in vacuo* the product was obtained as a white solid (8.98 g, 23.3 mmol, 88 %).

^{1}H NMR (300 MHz, $CDCl_3$) δ 8.19 (d, *J* = 2.1 Hz, 1H), 7.72 (d, *J* = 2.1 Hz, 1H), 7.61 – 7.28 (m, 10H), 4.26 (t, *J* = 7.7 Hz, 2H), 1.82 (quint, *J* = 7.5 Hz, 2H), 1.26 – 1.10 (m, 6H), 0.77 (t, *J* = 6.8 Hz, 3H).

^{13}C NMR (75 MHz, $CDCl_3$) δ 144.34, 134.87, 132.43, 131.04, 130.39, 129.97, 129.58, 126.17, 123.69, 123.47, 121.36, 49.61, 30.94, 29.87, 25.98, 22.36, 13.92.

Melting point: 110°C

Elemental analysis $C_{21}H_{25}BrN_2$ calc.: C: 65.46 %, H: 6.54 %, N: 7.27 %.
found: C: 64.88 %, H: 6.41 %, N: 7.14 %.

3-octyl-1,2-diphenyl-1*H*-imidazol-3-ium bromide [27]

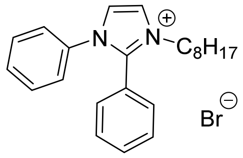

In an Ace pressure tube 1,2-diphenyl-1*H*-imidazole **13** (33 g, 150 mmol) and 1-bromooctane (31.9 g, 165 mmol) were dissolved in acetonitrile (30 mL). The reaction was stirred for 72 h at 90 °C. When the reaction was complete, the solvent was evaporated and the residue dissolved in a small amount of DCM. The solution was then poured into a large excess of diethyl ether. The precipitate formed was filtered off and washed with diethyl ether. After drying *in vacuo* the product was obtained as a white solid (40.3 g, 97.6 mmol, 65 %).

^{1}H NMR (300 MHz, $CDCl_3$) δ 8.17 (d, *J* = 2.1 Hz, 1H), 7.71 (d, *J* = 2.1 Hz, 1H), 7.59 – 7.27 (m, 10H), 4.23 (t, *J* = 7.5 Hz, 2H), 1.80 (quint, *J* = 7.4 Hz, 2H), 1.23 – 1.03 (m, 10H), 0.77 (t, *J* = 6.8 Hz, 3H).

^{13}C NMR (75 MHz, $CDCl_3$) δ 144.3, 134.8, 132.4, 131.0, 130.4, 129.9, 129.6, 126.1, 123.7, 123.5, 121.3, 49.6, 31.6, 29.9, 28.9, 28.8, 26.3, 22.6, 14.1.

Melting point: 121°C

Elemental analysis $C_{23}H_{29}BrN_2$ calc.: C: 66.82 %, H: 7.02 %, N: 6.87 %.
found: C: 67.12 %, H: 7.38 %, N: 6.81 %.

1,2-diphenyl-3-undecyl-1*H*-imidazol-3-ium bromide [28]

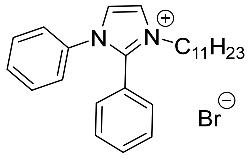

In an Ace pressure tube 1,2-diphenyl-1*H*-imidazole **13** (33 g, 150 mmol) and 1-bromoundecane (39.6 g, 165 mmol) were dissolved in acetonitrile (30 mL). The reaction was stirred for 72 h at 90 °C. When the reaction was complete, the solvent was evaporated and the residue dissolved in a small amount of DCM. The solution was then poured into a large excess of diethyl ether. The precipitate formed was filtered off and washed with diethyl ether. After drying *in vacuo* the product was obtained as a white solid (58.5 g, 128 mmol, 85.5 %).

^{1}H NMR (300 MHz, $CDCl_3$) δ 8.18 (d, *J* = 2.1 Hz, 1H), 7.72 (d, *J* = 2.1 Hz, 1H), 7.61 – 7.28 (m, 11H), 4.25 (t, *J* = 7.6 Hz, 2H), 1.81 (quint, *J* = 7.4 Hz, 2H), 1.25 – 1.09 (m, 16H), 0.81 (t, *J* = 6.8 Hz, 3H).

^{13}C NMR (75 MHz, $CDCl_3$) δ 144.3, 134.8, 132.4, 131.0, 130.3, 129.9, 129.5, 126.1, 123.7, 123.4, 121.3, 49.5, 31.8, 29.9, 29.5, 29.4, 29.3, 29.2, 28.8, 26.3, 22.6, 14.1.

Melting point: 126°C

Elemental analysis $C_{26}H_{35}BrN_2$ calc.: C: 68.56 %, H: 7.75 %, N: 6.15 %.
found: C: 68.91 %, H: 7.78 %, N: 6.16 %.

3-butyl-1-(2-methylphenyl)-2-phenyl-1*H*-imidazol-3-ium bromide [29]

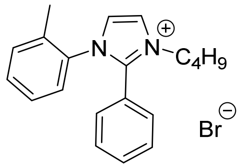

In an Ace pressure tube 1-(2-methylphenyl)-2-phenyl-1*H*-imidazole **14** (9.37 g, 40 mmol) and 1-brombutane (6.09 g, 44 mmol) were dissolved in acetonitrile (10 mL). The reaction was stirred for 72 h at 90 °C. When the reaction was complete, the solvent was evaporated and the residue dissolved in a small amount of DCM. The solution was then poured into a large excess of diethyl ether. The precipitate formed was filtered off and washed with diethyl ether. After drying *in vacuo* the product was obtained as a white solid (13.1 g, 35.3 mmol, 88 %).

^{1}H NMR (300 MHz, $CDCl_3$) δ 8.40 (d, *J* = 2.1 Hz, 1H), 7.54 – 7.29 (m, 8H), 7.24 – 7.16 (m, 2H), 4.38 (t, *J* = 7.7 Hz, 2H), 2.07 (s, 3H), 1.87 (quint, *J* = 7.5 Hz, 2H), 1.28 (sext, *J* = 7.5 Hz, 2H), 0.83 (t, *J* = 7.3 Hz, 3H).

^{13}C NMR (75 MHz, $CDCl_3$) δ 144.7, 134.1, 133.7, 132.5, 131.5, 131.0, 130.4, 129.5, 128.1, 127.6, 123.9, 123.7, 121.1, 49.6, 32.0, 19.5, 17.6, 13.4.

Melting point: 121°C

Elemental analysis $C_{20}H_{23}BrN_2$ calc.: C: 64.69 %, H: 6.24 %, N: 7.54 %.

found: C: 64.77 %, H: 6.31 %, N: 7.53 %.

3-hexyl-1-(2-methylphenyl)-2-phenyl-1*H*-imidazol-3-ium bromide [30]

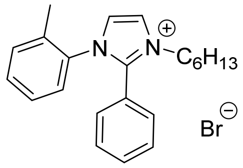

In an Ace pressure tube 1-(2-methylphenyl)-2-phenyl-1*H*-imidazole **14** (35.1 g, 150 mmol) and 1-bromohexane (27.5 g, 165 mmol) were dissolved in acetonitrile (30 mL). The reaction was stirred for 72 h at 90 °C. When the reaction was complete, the solvent was evaporated and the residue dissolved in a small amount of DCM. The solution was then poured into a large excess of diethyl ether. The precipitate formed was filtered off and washed with diethyl ether. After drying *in vacuo* the product was obtained as a white solid (52.1 g, 130 mmol, 87 %).

^{1}H NMR (300 MHz, $CDCl_3$) δ 8.45 (d, *J* = 2.1 Hz, 1H), 7.55 – 7.30 (m, 8H), 7.24 – 7.18 (m, 2H), 4.38 (t, *J* = 7.5 Hz, 2H), 2.07 (s, 3H), 1.87 (quint, *J* = 7.4 Hz, 2H), 1.29 – 1.13 (m, 6H), 0.79 (t, *J* = 6.8 Hz, 3H).

^{13}C NMR (75 MHz, $CDCl_3$) δ 144.8, 134.2, 133.8, 132.6, 131.6, 131.1, 130.5, 129.6, 128.2, 127.7, 124.1, 123.7, 121.3, 49.9, 31.0, 30.0, 26.0, 22.4, 17.7, 14.0.

Melting point: 121°C

Elemental analysis $C_{22}H_{27}BrN_2$ calc.: C: 66.16 %, H: 6.81 %, N: 7.01 %.

found: C: 66.42 %, H: 6.45 %, N: 7.01 %.

1-(2-methylphenyl)-3-octyl-2-phenyl-1*H*-imidazol-3-ium bromide [31]

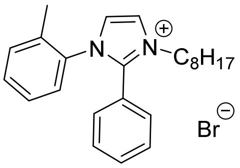

In an Ace pressure tube 1-(2-methylphenyl)-2-phenyl-1*H*-imidazole **14** (35.1 g, 150 mmol) and 1-bromooctane (31.9 g, 165 mmol) were dissolved in acetonitrile (30 mL). The reaction was stirred for 72 h at 90 °C. When the reaction was complete, the solvent was evaporated and the residue dissolved in a small amount of DCM. The solution was then poured into a large excess of a mixture of *iso*hexane and diethyl ether. The precipitate formed was filtered off and washed with isohexane. After drying *in vacuo* the product was obtained as a white solid (56.9 g, 133 mmol, 89 %).

^{1}H NMR (300 MHz, $CDCl_3$) δ 8.46 (d, *J* = 2.1 Hz, 1H), 7.55 – 7.31 (m, 8H), 7.26 – 7.19 (m, 2H), 4.40 (t, *J* = 7.7 Hz, 2H), 2.09 (s, 3H), 1.89 (quint, *J* = 7.4 Hz, 2H), 1.30 – 1.13 (m, 10H), 0.84 (t, *J* = 6.8 Hz, 3H).

^{13}C NMR (75 MHz, $CDCl_3$) δ 144.8, 134.2, 133.8, 132.6, 131.6, 131.1, 130.5, 129.6, 128.3, 127.7, 124.1, 123.7, 121.3, 49.9, 31.7, 30.1, 29.1, 28.9, 26.4, 22.7, 17.7, 14.2.

Melting point: 109°C

Elemental analysis $C_{24}H_{31}BrN_2 \cdot 0.15\ C_8H_{17}Br$ calc: C: 68.11 %, H: 7.61 %, N: 6.30 %.
found: C: 68.51 %, H: 7.51 %, N: 6.67 %.

1-(2-methylphenyl)- 2-phenyl-3-undecyl-1*H*-imidazol-3-ium bromide [32]

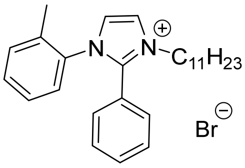

In an Ace pressure tube 1-(2-methylphenyl)-2-phenyl-1*H*-imidazole **14** (3.51 g, 15 mmol) and 1-bromoundecane (3.96 g, 16.5 mmol) were dissolved in acetonitrile (30 mL). The reaction was stirred for 72 h at 90 °C. When the reaction was complete, the solvent was evaporated and the residue dissolved in a small amount of DCM. The solution was then poured into a large excess of a mixture of diethyl ether and isohexane. The precipitate formed was filtered off and washed with isohexane. After drying *in vacuo* the product was obtained as a white solid (6.24 g, 13.3 mmol, 89 %).

^{1}H NMR (300 MHz, $CDCl_3$) δ 8.30 (d, *J* = 1.7 Hz, 1H), 7.52 – 7.23 (m, 8H), 7.18 – 7.10 (m, 2H), 4.29 (t, *J* = 7.5 Hz, 2H), 2.00 (s, 3H), 1.86 – 1.74 (m, 2H), 1.21 – 1.02 (m, *J* = 10.7 Hz, 16H), 0.76 (t, *J* = 6.7 Hz, 3H).

^{13}C NMR (75 MHz, $CDCl_3$) δ 144.6, 134.0, 133.5, 132.4, 131.4, 130.9, 130.2, 129.4, 127.9, 127.4, 123.7, 123.6, 120.9, 49.7, 31.7, 29.8, 29.3, 29.3, 29.2, 29.1, 28.7, 26.1, 22.5, 17.5, 14.0.

Melting point: 127°C

Elemental analysis $C_{27}H_{37}BrN_2 \cdot 0.45\ H_2O$ calc.: C: 67.90 %, H: 8.00 %, N: 5.87 %.
found: C: 67.46 %, H: 7.56 %, N: 6.22 %.

3-butyl-1-(4-methoxyphenyl)-2-phenyl-1*H*-imidazol-3-ium bromide [33]

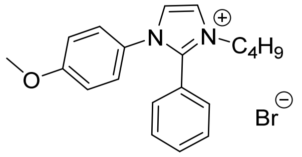

In an Ace pressure tube 1-(4-methoxyphenyl)-2-phenyl-1*H*-imidazole **15** (7.51 g, 30 mmol) and 1-brombutane (4.61 g, 33 mmol) were dissolved in acetonitrile (10 mL). The reaction was stirred for 72 h at 90 °C. When the reaction was complete, the solvent was evaporated and the residue dissolved in a small amount of DCM. The solution was then poured into a large excess of a mixture of diethyl ether and isohexane. The precipitate formed was filtered off and washed with isohexane. After drying *in vacuo* the product was obtained as a brown glassy solid (9.59 g, 24.8 mmol, 83 %).

^{1}H NMR (300 MHz, $CDCl_3$) δ 8.17 (s, 1H), 7.66 (s, 1H), 7.56 – 7.41 (m, 5H), 7.28 (d, *J* = 8.9 Hz, 2H), 6.79 (d, *J* = 8.7 Hz, 2H), 4.24 (t, *J* = 7.5 Hz, 2H), 3.72 (s, 3H), 1.80 (quint, *J* = 7.3 Hz, 2H), 1.24 (sext, *J* = 7.3 Hz, 2H), 0.78 (t, *J* = 7.3 Hz, 3H).

^{13}C NMR (75 MHz, $CDCl_3$) δ 160.4, 144.1, 132.2, 130.7, 129.4, 127.2, 123.8, 123.1, 121.1, 114.8, 55.5, 49.1, 31.7, 19.4, 13.2.

Melting point: amorphous

Elemental analysis $C_{20}H_{23}BrN_2O$ calc.: C: 62.02%, H: 5.99%, N: 7.23%.
found: C: 61.85%, H: 6.34%, N: 7.20%.

3-hexyl-1-(4-methoxyphenyl)-2-phenyl-1*H*-imidazol-3-ium bromide [34]

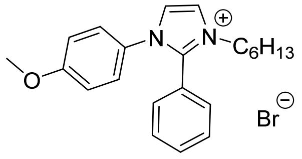

In an Ace pressure tube 1-(4-methoxyphenyl)-2-phenyl-1*H*-imidazole **15** (7.51 g, 30 mmol) and 1-bromhexane (5.5 g, 33 mmol) were dissolved in acetonitrile (10 mL). The reaction was stirred for 72 h at 90 °C. When the reaction was complete, the solvent was evaporated and the residue dissolved in a small amount of DCM. The solution was then poured into a large excess of diethyl ether. The precipitate formed was filtered off and washed with diethyl ether. After drying *in vacuo* the product was obtained as a white solid (11.9 g, 28.6 mmol, 96 %).

^{1}H NMR (300 MHz, $CDCl_3$) δ 8.14 (d, *J* = 2.1 Hz, 1H), 7.67 (d, *J* = 2.1 Hz, 1H), 7.60 – 7.42 (m, 5H), 7.31 (d, *J* = 8.9 Hz, 2H), 6.81 (d, *J* = 8.9 Hz, 2H), 4.25 (t, *J* = 7.7 Hz, 2H), 3.74 (s, 3H), 1.82 (quint, *J* = 7.5 Hz, 2H), 1.27 – 1.11 (m, 6H), 0.78 (t, *J* = 6.7 Hz, 3H).

^{13}C NMR (75 MHz, $CDCl_3$) δ 160.7, 144.4, 132.4, 131.0, 129.6, 127.5, 127.5, 124.0, 123.2, 121.5, 115.0, 55.7, 49.6, 31.0, 29.9, 26.0, 22.4, 13.9.

Melting point: 122°C

Elemental analysis $C_{22}H_{27}BrN_2O$ calc.: C: 63.62%, H: 6.55%, N: 6.74%.
found: C: 63.48%, H: 6.52%, N: 6.82%.

1-(4-methoxyphenyl)-3-octyl-2-phenyl-1*H*-imidazol-3-ium bromide [35]

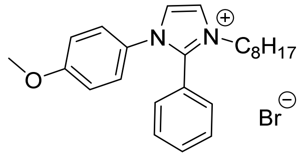

In an Ace pressure tube 1-(4-methoxyphenyl)-2-phenyl-1*H*-imidazole **15** (7.51 g, 30 mmol) and 1-bromoctane (6.37 g, 33 mmol) were dissolved in acetonitrile (10 mL). The reaction was stirred for 72 h at 90 °C. When the reaction was complete, the solvent was evaporated and the residue dissolved in a small amount of DCM. The solution was then poured into a large excess of diethyl ether. The precipitate formed was filtered off and washed with diethyl ether. After drying *in vacuo* the product was obtained as a white solid (12.3 g, 27.7 mmol, 92.4 %).

^{1}H NMR (300 MHz, $CDCl_3$) δ 8.13 (d, *J* = 1.6 Hz, 1H), 7.67 (d, *J* = 1.6 Hz, 1H), 7.61 – 7.43 (m, 5H), 7.32 (d, *J* = 8.9 Hz, 2H), 6.82 (d, *J* = 8.9 Hz, 2H), 4.25 (t, *J* = 7.7 Hz, 2H), 3.75 (s, 3H), 1.83 (quint, *J* = 7.3 Hz, 2H), 1.27 – 1.11 (m, 10H), 0.82 (t, *J* = 6.8 Hz, 3H).

^{13}C NMR (75 MHz, $CDCl_3$) δ 160.6, 144.3, 132.3, 131.0, 129.6, 127.4, 124.0, 123.2, 121.4, 115.0, 55.7, 49.6, 31.6, 29.9, 29.0, 28.8, 26.3, 22.6, 14.1.

Melting point: 122°C

Elemental analysis $C_{24}H_{31}BrN_2O \cdot 0.25\ H_2O$ calc.: C: 64.35%, H: 7.09%, N: 6.25%.
found: C: 64.09%, H: 6.91%, N: 6.51%.

1-(4-methoxyphenyl)-2-phenyl-3-undecyl-1*H*-imidazol-3-ium bromide [36]

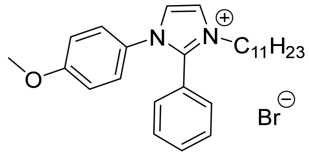

In an Ace pressure tube 1-(4-methoxyphenyl)-2-phenyl-1*H*-imidazole **15** (7.51 g, 30 mmol) and 1-bromoundecane (7.92 g, 33 mmol) were dissolved in acetonitrile (10 mL). The reaction was stirred for 72 h at 90 °C. When the reaction was complete, the solvent was evaporated and the residue dissolved in a small amount of DCM. The solution was then poured into a large excess of diethyl ether. The precipitate formed was filtered off and washed with diethyl ether. After drying *in vacuo* the product was obtained as a white solid (13.6 g, 28.0 mmol, 93.4 %).

^{1}H NMR (300 MHz, $CDCl_3$) δ 8.12 (d, *J* = 1.8 Hz, 1H), 7.66 (d, *J* = 1.8 Hz, 1H), 7.57 – 7.41 (m, 5H), 7.29 (d, *J* = 8.9 Hz, 2H), 6.79 (d, *J* = 8.9 Hz, 2H), 4.22 (t, *J* = 7.7 Hz, 2H), 3.72 (s, 3H), 1.80 (quint, *J* = 7.3 Hz, 2H), 1.22 – 1.08 (m, 16H), 0.81 (t, *J* = 6.8 Hz, 3H).

^{13}C NMR (75 MHz, $CDCl_3$) δ 160.6, 144.3, 132.3, 130.9, 129.5, 127.4, 123.9, 123.1, 121.4, 114.9, 55.6, 49.5, 31.8, 29.9, 29.5, 29.4, 29.6, 29.2, 28.8, 26.2, 22.6, 14.8.

Melting point: amorphous

Elemental analysis $C_{27}H_{37}BrN_2O$ calc.: C: 66.80%, H 7.68%, N: 5.77%.
found: C: 66.45%, H: 8.04%, N: 5.76%.

3-butyl-1-(2,4-difluorophenyl)-2-phenyl-1*H*-imidazol-3-ium bromide [37]

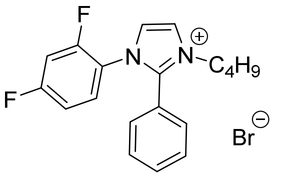

In an Ace pressure tube 1-(2,4-difluorophenyl)-2-phenyl-1*H*-imidazole **16** (6.41 g, 25 mmol) and 1-brombutane (3.81 g, 27.5 mmol) were dissolved in acetonitrile (10 mL). The reaction was stirred for 72 h at 90 °C. When the reaction was complete, the solvent was evaporated and the residue dissolved in a small amount of DCM. The solution was then poured into a large excess of diethyl ether. The precipitate formed was filtered off and washed with diethyl ether. After drying *in vacuo* the product was obtained as an off-white solid (7.46 g, 19.0 mmol, 76 %).

^{1}H NMR (300 MHz, $CDCl_3$) δ 8.22 (td, *J* = 8.7, 5.7 Hz, 1H), 8.06 (d, *J* = 2.1 Hz, 1H), 7.72 (d, *J* = 7.1 Hz, 2H), 7.63 (d, *J* = 2.1 Hz, 1H), 7.58 – 7.43 (m, 3H), 6.99 – 6.79 (m, 2H), 4.22 (t, *J* = 7.7 Hz, 2H), 1.88 (quint, *J* = 7.5 Hz, 2H), 1.31 (sext, *J* = 7.5 Hz, 2H), 0.85 (t, *J* = 7.4 Hz, 3H).

^{13}C NMR (75 MHz, $CDCl_3$) δ 163.8 (dd, *J* = 255.9, 11.0 Hz), 156.7 (dd, *J* = 255.4, 12.7 Hz), 158.4, 158.3, 155.1, 154.9, 146.1, 132.8, 132.0, 131.9, 130.8, 129.6, 124.4, 123.2, 120.9, 119.1 (dd, *J* = 12.4, 4.1 Hz), 113.2 (dd, *J* = 22.7, 3.9 Hz), 105.1 (dd, *J* = 26.9, 22.6 Hz), 49.6, 31.7, 19.7, 13.4.

Melting point: 102°C

Elemental analysis $C_{19}H_{19}BrF_2N_2$ calc.: C: 58.03%, H: 4.87%, N: 7.12%.
found: C: 57.67%, H: 4.92%, N: 7.10%.

1-(2,4-difluorophenyl)-3-hexyl-2-phenyl-1*H*-imidazol-3-ium bromide [38]

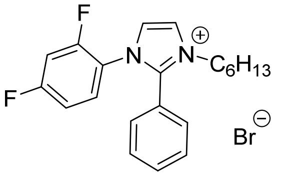

In an Ace pressure tube 1-(2,4-difluorophenyl)-2-phenyl-1*H*-imidazole **16** (6.41 g, 25 mmol) and 1-bromhexane (4.52 g, 27.5 mmol) were dissolved in acetonitrile (10 mL). The reaction was stirred for 72 h at 90 °C. When the reaction was complete, the solvent was evaporated and the residue dissolved in a small amount of DCM. The solution was then poured into a large excess of diethyl ether. The precipitate formed was filtered off and washed with diethyl ether. After drying *in vacuo* the product was obtained as a white solid (9.67 g, 23.0 mmol, 92 %).

^{1}H NMR (300 MHz, $CDCl_3$) δ 8.23 (td, *J* = 8.7, 5.7 Hz, 1H), 8.00 (d, *J* = 2.2 Hz, 1H), 7.74 (d, *J* = 7.1 Hz, 2H), 7.61 (d, *J* = 2.1 Hz, 1H), 7.60 – 7.43 (m, 3H), 7.00 – 6.80 (m, 2H), 4.22 (t, *J* = 7.6 Hz, 2H), 1.90 (quint, *J* = 7.5 Hz, 2H), 1.35 – 1.14 (m, 6H), 0.82 (t, *J* = 6.8 Hz, 3H).

^{13}C NMR (75 MHz, $CDCl_3$) δ 163.8 (dd, *J* = 256.0, 11.0 Hz), 156.7 (dd, *J* = 255.4, 12.7 Hz), 146.1, 132.8, 132.1, 131.9, 130.8, 129.6, 124.4, 123.1, 120.9, 119.1 (dd, *J* = 12.4, 4.1 Hz), 113.2 (dd, *J* = 22.7, 3.9 Hz), 105.1 (dd, *J* = 27.0, 22.6 Hz), 49.8, 31.0, 29.7, 26.0, 22.4, 14.0.

Melting point: 141°C

Elemental analysis $C_{21}H_{23}BrF_2N_2$ calc.: C: 59.87%, H: 5.50%, N: 6.65%.
found: C: 59.82%, H: 5.48%, N: 6.61%.

1-(2,4-difluorophenyl)-3-octyl-2-phenyl-1*H*-imidazol-3-ium bromide [39]

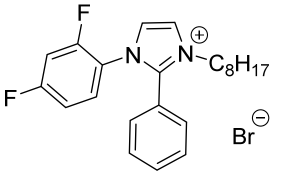

In an Ace pressure tube 1-(2,4-difluorophenyl)-2-phenyl-1*H*-imidazole **16** (6.41 g, 25 mmol) and 1-bromoctane (5.31 g, 27.5 mmol) were dissolved in acetonitrile (10 mL). The reaction was stirred for 72 h at 90 °C. When the reaction was complete, the solvent was evaporated and the residue dissolved in a small amount of DCM. The solution was then poured into a large excess of diethyl ether. The precipitate formed was filtered off and washed with diethyl ether. After drying *in vacuo* the product was obtained as a white solid (10.4 g, 23.1 mmol, 92 %).

^{1}H NMR (300 MHz, $CDCl_3$) δ 8.20 (td, *J* = 8.7, 5.7 Hz, 1H), 8.10 (d, *J* = 2.1 Hz, 1H), 7.70 (d, *J* = 7.1 Hz, 2H), 7.65 (d, *J* = 2.1 Hz, 1H), 7.56 – 7.42 (m, 3H), 6.94 – 6.80 (m, 2H), 4.20 (t, *J* = 7.7 Hz, 2H), 1.87 (quint, *J* = 7.4 Hz, 2H), 1.27 – 1.11 (m, 10H), 0.81 (t, *J* = 6.8 Hz, 3H).

^{13}C NMR (75 MHz, $CDCl_3$) δ 163.7 (dd, *J* = 255.9, 11.1 Hz), 156.6 (dd, *J* = 255.6, 12.7 Hz), 145.8, 132.7, 131.9, 131.7, 130.7, 129.5, 124.4, 123.3, 120.8, 119.0 (dd, *J* = 12.3, 4.2 Hz), 113.1 (dd, *J* = 22.7, 3.9 Hz), 105.0 (dd, *J* = 27.0, 22.6 Hz), 49.7, 31.6, 29.7, 28.9, 28.7, 26.3, 22.5, 14.0.

Melting point: amorphous

Elemental analysis $C_{23}H_{27}BrF_2N_2$ calc.: C: 61.47%, H: 6.06%, N: 6.23%.
found: C: 61.44%, H: 6.09%, N: 6.31%.

1-(2,4-difluorophenyl)- 2-phenyl-3-undecyl-1*H*-imidazol-3-ium bromide [40]

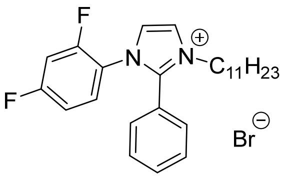

In an Ace pressure tube 1-(2,4-difluorophenyl)-2-phenyl-1*H*-imidazole **16** (6.41 g, 25 mmol) and 1-bromoundecane (6.60 g, 27.5 mmol) were dissolved in acetonitrile (10 mL). The reaction was stirred for 72 h at 90 °C. When the reaction was complete, the solvent was evaporated and the residue dissolved in a small amount of DCM. The solution was then poured into a large excess of diethyl ether. The precipitate formed was filtered off and washed with diethyl ether. After drying *in vacuo* the product was obtained as a white solid (11.2 g, 22.8 mmol, 91 %).

^{1}H NMR (300 MHz, $CDCl_3$) δ 8.20 (td, *J* = 8.7, 5.7 Hz, 1H), 8.09 (d, *J* = 2.1 Hz, 1H), 7.70 (d, *J* = 7.1 Hz, 2H), 7.65 (d, *J* = 2.1 Hz, 1H), 7.56 – 7.41 (m, 3H), 6.98 – 6.77 (m, 2H), 4.20 (t, *J* = 7.7 Hz, 2H), 1.87 (quint, *J* = 7.5 Hz, 2H), 1.26 – 1.11 (m, 16H), 0.83 (t, *J* = 6.8 Hz, 3H).

^{13}C NMR (75 MHz, $CDCl_3$) δ 163.7 (dd, *J* = 255.9, 11.0 Hz), 156.6 (dd, *J* = 255.6, 12.7 Hz), 145.9, 132.7, 131.9, 131.8, 130.7, 129.5, 124.4, 123.3, 120.9, 119.0 (dd, *J* = 12.4, 4.2 Hz), 113.1 (dd, *J* = 22.7, 3.9 Hz), 105.0 (dd, *J* = 27.0, 22.6 Hz), 49.7, 31.9, 29.7, 29.5, 29.5, 29.3, 29.3, 28.8, 26.3, 22.7, 14.1.

Melting point: 104 °C

Elemental analysis $C_{26}H_{33}BrF_2N_2$ calc.: C: 63.54%, H: 6.77%, N: 5.70%.
found: C: 63.74%, H: 6.68%, N: 5.76%.

3-butyl-2-methyl-1-phenyl-1*H*-imidazol-3-ium bromide [41]

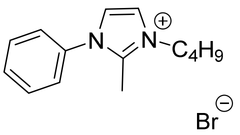

In an Ace pressure tube 2-methyl-1-phenyl-1*H*-imidazole **17** (4.75 g, 30 mmol) and 1-bromobutane (4.61 g, 33 mmol) were dissolved in acetonitrile (10 mL). The reaction was stirred for 72 h at 90 °C. When the reaction was complete, the solvent was evaporated and the residue dissolved in a small amount of DCM. The solution was then poured into a large excess of diethyl ether. The precipitate formed was filtered off and washed with diethyl ether. After drying *in vacuo* the product was obtained as an off-white solid (8.8 g, 29.8 mmol, 99 %).

^{1}H NMR (300 MHz, $CDCl_3$) δ 7.78 (d, J = 2.1 Hz, 1H), 7.55 – 7.43 (m, 5H), 7.33 (d, J = 2.1 Hz, 1H), 4.28 (t, J = 7.6 Hz, 2H), 2.66 (s, 3H), 2.01 – 1.73 (m, 2H), 1.36 (sext, J = 7.6 Hz, 2H), 0.88 (t, J = 7.3 Hz, 3H).

^{13}C NMR (75 MHz, $CDCl_3$) δ 144.5, 134.6, 131.0, 130.4, 126.3, 122.4, 122.3, 49.4, 31.5, 19.9, 13.7, 11.9.

Melting point: 120 °C

Elemental analysis $C_{14}H_{19}BrN_2$ calc.: C: 56.96 %, H: 6.49 %, N: 9.49 %.
found: C: 56.67 %, H: 6.53 %, N: 9.55 %.

3-hexyl-2-methyl-1-phenyl-1*H*-imidazol-3-ium bromide [42]

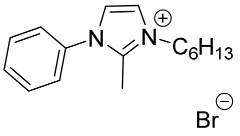

In an Ace pressure tube 2-methyl-1-phenyl-1*H*-imidazole **17** (4.75 g, 30 mmol) and 1-bromohexane (5.50 g, 33 mmol) were dissolved in acetonitrile (10 mL). The reaction was stirred for 72 h at 90 °C. When the reaction was complete, the solvent was evaporated and the residue dissolved in a small amount of DCM. The solution was then poured into a large excess of diethyl ether. The precipitate formed was filtered off and washed with diethyl ether. After drying *in vacuo* the product was obtained as an off-white solid (8.65 g, 26.7 mmol, 89 %).

^{1}H NMR (300 MHz, $CDCl_3$) δ 7.77 (d, J = 2.1 Hz, 1H), 7.62 – 7.50 (m, 5H), 7.37 (d, J = 2.1 Hz, 1H), 4.32 (t, J = 7.7 Hz, 2H), 2.71 (s, 3H), 1.89 (quint, J = 7.6 Hz, 2H), 1.43 – 1.20 (m, 6H), 0.84 (t, J = 7.0 Hz, 3H).

^{13}C NMR (75 MHz, $CDCl_3$) δ 144.6, 134.6, 131.0, 130.4, 126.3, 122.4, 122.3, 49.7, 31.3, 29.5, 26.3, 22.5, 14.0, 12.0.

Melting point: 85 °C

Elemental analysis $C_{16}H_{23}BrN_2$ calc.: C: 59,45 %, H: 7.17 %, N: 8.67 %.
found: C: 59.55 %, H: 7.21 %, N: 8.77 %

2-methyl-3-octyl-1-phenyl-1*H*-imidazol-3-ium bromide [43]

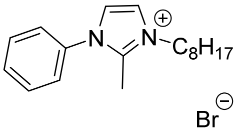

In an Ace pressure tube 2-methyl-1-phenyl-1*H*-imidazole **17** (4.75 g, 30 mmol) and 1-bromooctane (6.37 g, 33 mmol) were dissolved in acetonitrile (10 mL). The reaction was stirred for 72 h at 90 °C. When the reaction was complete, the solvent was evaporated and the residue dissolved in a small amount of DCM. The solution was then poured into a large excess of diethyl ether. The precipitate formed was filtered off and washed with diethyl ether. After drying *in vacuo* the product was obtained as a white solid (10.1 g, 28.8 mmol, 96 %).

^{1}H NMR (300 MHz, $CDCl_3$) δ 7.75 (d, *J* = 2.1 Hz, 1H), 7.64 – 7.53 (m, 5H), 7.37 (d, *J* = 2.1 Hz, 1H), 4.34 (t, *J* = 7.6 Hz, 2H), 2.73 (s, 3H), 1.91 (quint, *J* = 7.4 Hz, 2H), 1.46 – 1.16 (m, 10H), 0.85 (t, *J* = 6.7 Hz, 3H).

^{13}C NMR (75 MHz, $CDCl_3$) δ 144.5, 134.6, 131.0, 130.4, 126.3, 122.3, 122.3, 49.6, 31.7, 29.6, 29.1, 26.6, 22.6, 14.1, 11.9.

Melting point: 85 °C

Elemental analysis $C_{18}H_{27}BrN_2$ calc.: C: 61.54%, H: 7.75%, N: 7.97%.
found: C: 61.71%, H: 7.74%, N: 8.08%.

2-methyl-1-phenyl-3-undecyl-1*H*-imidazol-3-ium bromide [44]

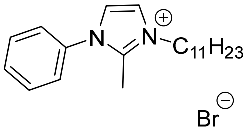

In an Ace pressure tube 2-methyl-1-phenyl-1*H*-imidazole **17** (4.75 g, 30 mmol) and 1-bromoundecane (7.92 g, 33 mmol) were dissolved in acetonitrile (10 mL). The reaction was stirred for 72 h at 90 °C. When the reaction was complete, the solvent was evaporated and the residue dissolved in a small amount of DCM. The solution was then poured into a large excess of diethyl ether. The precipitate formed was filtered off and washed with diethyl ether. After drying *in vacuo* the product was obtained as a white solid (11.1 g, 28.3 mmol, 94 %).

^{1}H NMR (300 MHz, $CDCl_3$) δ 7.76 (d, *J* = 2.1 Hz, 1H), 7.61 – 7.50 (m, 5H), 7.37 (d, *J* = 2.1 Hz, 1H), 4.31 (t, *J* = 7.7 Hz, 2H), 2.71 (s, 3H), 1.88 (quint, *J* = 7.5 Hz, 2H), 1.40 – 1.15 (m, 16H), 0.82 (t, *J* = 6.7 Hz, 3H).

^{13}C NMR (75 MHz, $CDCl_3$) δ 144.3, 134.5, 130.9, 130.3, 126.2, 122.3, 122.3, 49.5, 31.8, 29.5, 29.4, 29.4, 29.2, 29.1, 26.5, 22.6, 14.1, 11.9.

Melting point: 66 °C

Elemental analysis $C_{21}H_{33}BrN_2$ calc.: C: 64.11%, H: 8.45%, N: 7.12%.
found: C: 63.95%, H: 8.39%, N: 7.20%.

3-butyl-2-methyl-1-(2-methylphenyl)-1*H*-imidazol-3-ium bromide [45]

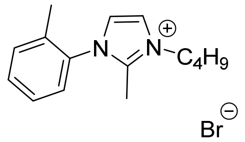

In an Ace pressure tube 1-(2-methylphenyl)-2-methyl-1*H*-imidazole **18** (2.58 g, 15 mmol) and 1-bromobutane (2.31 g, 16.5 mmol) were dissolved in acetonitrile (5 mL). The reaction was stirred for 24 h at 90 °C. When the reaction was complete, the solvent was evaporated and the residue dissolved in a small amount of DCM. The solution was then poured into a large excess of diethyl ether. The precipitate formed was filtered off and washed with diethyl ether. After drying *in vacuo* the product was obtained as a yellow solid (4.54 g, 14.7 mmol, 98 %).

^{1}H NMR (500 MHz, $CDCl_3$) δ 7.93 (d, *J* = 2.1 Hz, 1H), 7.51 – 7.40 (m, 2H), 7.38 – 7.29 (m, 2H), 7.25 (d, *J* = 2.1 Hz, 1H), 4.52 – 4.29 (m, 2H), 2.55 (s, 3H), 2.06 (s, 3H), 1.92 – 1.81 (m, 2H), 1.39 (sext, *J* = 7.6 Hz, 2H), 0.93 (t, *J* = 7.4 Hz, 3H).

^{13}C NMR (75 MHz, $CDCl_3$) δ 144.5, 134.4, 133.3, 131.8, 131.4, 127.9, 127.5, 122.8, 122.3, 49.4, 31.5, 19.7, 17.4, 13.6, 11.2.

Melting point: 122 °C

Elemental analysis $C_{15}H_{21}BrN_2$ calc.: C: 58.26%, H: 6.84%, N: 9.06%.
found: C: 58.59%, H: 7.01%, N: 9.11%.

3-hexyl-2-methyl-1-(2-methylphenyl)-1*H*-imidazol-3-ium bromide [46]

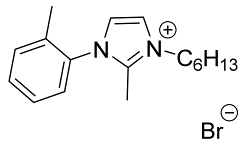

In an Ace pressure tube 1-(2-methylphenyl)-2-methyl-1*H*-imidazole **18** (2.58 g, 15 mmol) and 1-bromohexane (2.75 g, 16.5 mmol) were dissolved in acetonitrile (5 mL). The reaction was stirred for 24 h at 90 °C. When the reaction was complete, the solvent was evaporated and the residue dissolved in a small amount of DCM. The solution was then poured into a large excess of diethyl ether. The precipitate formed was filtered off and washed with diethyl ether. After drying *in vacuo* the product was obtained as a yellow solid (4.85 g, 14.4 mmol, 96 %).

^{1}H NMR (300 MHz, $CDCl_3$) δ 7.94 (d, *J* = 2.0 Hz, 1H), 7.52 – 7.40 (m, 2H), 7.38 – 7.30 (m, 2H), 7.28 (d, *J* = 2.0 Hz, 1H), 4.40 (t, *J* = 7.4 Hz, 2H), 2.56 (s, 3H), 2.07 (s, 3H), 1.90 (quint, *J* = 7.4 Hz, 2H), 1.40 – 1.19 (m, 6H), 0.81 (t, *J* = 7.0 Hz, 3H).

^{13}C NMR (75 MHz, $CDCl_3$) δ 144.4, 134.3, 133.2, 131.7, 131.3, 127.8, 127.4, 122.6, 122.2, 49.5, 31.0, 29.4, 25.9, 22.3, 17.3, 13.8, 11.2.

Melting point: 71 °C

Elemental analysis $C_{17}H_{25}BrN_2$ calc.: C: 60.54%, H: 7.47%, N: 8.31%.
found: C: 60.22%, H: 7.77%, N: 8.49%.

2-methyl-1-(2-methylphenyl)-3-octyl-1*H*-imidazol-3-ium bromide [47]

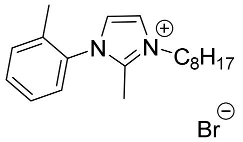

In an Ace pressure tube 1-(2-methylphenyl)-2-methyl-1*H*-imidazole **18** (2.58 g, 15 mmol) and 1-bromooctane (3.19 g, 16.5 mmol) were dissolved in acetonitrile (5 mL). The reaction was stirred for 24 h at 90 °C. When the reaction was complete, the solvent was evaporated and the residue dissolved in a small amount of DCM. The solution was then poured into a large excess of diethyl ether. The precipitate formed was filtered off and washed with diethyl ether. After drying *in vacuo* the product was obtained as a white solid (5.16 g, 14.1 mmol, 94 %).

^{1}H NMR (500 MHz, $CDCl_3$) δ 7.92 (d, *J* = 2.1 Hz, 1H), 7.53 (d, *J* = 7.9 Hz, 1H), 7.46 (td, *J* = 7.7, 1.2 Hz, 1H), 7.41 – 7.32 (m, 2H), 7.27 (d, *J* = 2.1 Hz, 1H), 4.51 – 4.33 (m, 2H), 2.58 (s, 3H), 2.09 (s, 3H), 1.97 – 1.84 (m, 2H), 1.39 – 1.15 (m, 11H), 0.82 (t, *J* = 7.0 Hz, 3H).

^{13}C NMR (75 MHz, $CDCl_3$) δ 144.5, 134.5, 133.3, 131.8, 131.5, 128.0, 127.6, 122.7, 122.3, 49.7, 31.7, 29.6, 29.0, 26.4, 22.6, 17.4, 14.0, 11.3.

Melting point: 93 °C

**Elemental analysis** $C_{19}H_{29}BrN_2$	calc.:	C: 62.46%, H: 8.00%, N: 7.67%.
	found:	C: 62.62%, H: 8.07%, N: 7.73%.

2-methyl-1-(2-methylphenyl)-3-undecyl-1*H*-imidazol-3-ium bromide [48]

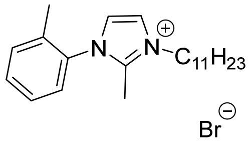

In an Ace pressure tube 1-(2-methylphenyl)-2-methyl-1*H*-imidazole **18** (2.58 g, 15 mmol) and 1-bromoundecane (3.96 g, 16.5 mmol) were dissolved in acetonitrile (5 mL). The reaction was stirred for 24 h at 90 °C. When the reaction was complete, the solvent was evaporated and the residue dissolved in a small amount of DCM. The solution was then poured into a large excess of diethyl ether. The precipitate formed was filtered off and washed with diethyl ether. After drying *in vacuo* the product was obtained as a yellow glassy solid (5.66 g, 13.9 mmol, 93 %).

^{1}H NMR (300 MHz, $CDCl_3$) δ 7.93 (s, 1H), 7.51 – 7.27 (m, 5H), 4.50 – 4.30 (m, 2H), 2.57 (s, *J* = 8.2 Hz, 3H), 2.07 (s, 3H), 1.96 – 1.83 (m, 2H), 1.45 – 1.04 (m, 18H), 0.80 (t, *J* = 6.7 Hz, 3H).

^{13}C NMR (75 MHz, $CDCl_3$) δ 144.4, 134.3, 133.2, 131.7, 131.3, 127.9, 127.5, 122.7, 122.3, 49.6, 31.7, 29.5, 29.4, 29.3, 29.3, 29.1, 29.0, 26.3, 22.5, 17.4, 14.0, 11.3.

Melting point: amorphous

**Elemental analysis** $C_{22}H_{35}BrN_2$	calc.:	C: 64.85%, H: 8.66%, N: 6.88%.
	found:	C: 64.60%, H: 8.71%, N: 6.75%.

3-butyl-1-(4-methoxyphenyl)-2-methyl-1*H*-imidazol-3-ium bromide [49]

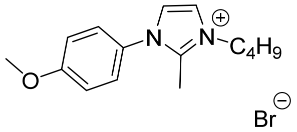

In an Ace pressure tube 1-(4-methoxyphenyl)-2-methyl-1*H*-imidazole **19** (1.88 g, 10 mmol) and 1-bromobutane (1.54 g, 11 mmol) were dissolved in acetonitrile (5 mL). The reaction was stirred for 24 h at 90 °C. When the reaction was complete, the solvent was evaporated and the residue dissolved in a small amount of DCM. The solution was then poured into a large excess of diethyl ether. The precipitate formed was filtered off and washed with diethyl ether. After drying *in vacuo* the product was obtained as a yellow solid (3.13 g, 9.6 mmol, 96 %).

^{1}H NMR (300 MHz, $CDCl_3$) δ 7.72 (d, *J* = 2.1 Hz, 1H), 7.53 (d, *J* = 9.0 Hz, 2H), 7.31 (d, *J* = 2.1 Hz, 1H), 7.02 (d, *J* = 9.0 Hz, 2H), 4.34 (t, *J* = 7.6 Hz, 2H), 3.85 (s, 3H), 2.71 (s, 3H), 1.90 (quint, *J* = 7.6 Hz, 2H), 1.45 (sext, *J* = 7.4 Hz, 2H), 0.97 (t, *J* = 7.3 Hz, 3H).

^{13}C NMR (75 MHz, $CDCl_3$) δ 161.3, 144.7, 127.7, 127.1, 122.6, 122.1, 115.4, 55.9, 49.4, 31.5, 19.9, 13.7, 11.8.

Melting point: 116 °C

Elemental analysis $C_{15}H_{21}BrN_2O$ calc.: C: 55.39%, H: 6.51%, N: 8.61%.
found: C: 55.28%, H: 6.43%, N: 8.50%.

3-hexyl-1-(4-methoxyphenyl)-2-methyl-1*H*-imidazol-3-ium bromide [50]

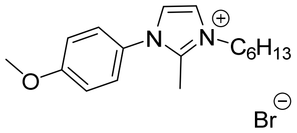

In an Ace pressure tube 1-(4-methoxyphenyl)-2-methyl-1*H*-imidazole **19** (1 g, 5.31 mmol) and 1-bromohexane (0.97 g, 5.84 mmol) were dissolved in tetrahydrofuran (5 mL). The reaction was stirred for 24 h at 90 °C. When the reaction was complete, the solvent was evaporated and the residue dissolved in a small amount of DCM. The solution was then poured into a large excess of diethyl ether. The precipitate formed was filtered off and washed with diethyl ether. After drying *in vacuo* the product was obtained as a white solid (1.63 g, 4.6 mmol, 87 %).

^{1}H NMR (300 MHz, $CDCl_3$) δ 7.65 (d, *J* = 2.1 Hz, 1H), 7.56 (d, *J* = 9.0 Hz, 2H), 7.29 (d, *J* = 2.1 Hz, 1H), 7.03 (d, *J* = 9.0 Hz, 2H), 4.33 (t, *J* = 7.8 Hz, 2H), 3.86 (s, 3H), 2.72 (s, 3H), 1.92 (quint, *J* = 7.6 Hz, 2H), 1.45 – 1.26 (m, 6H), 0.88 (t, *J* = 7.1 Hz, 3H).

^{13}C NMR (75 MHz, $CDCl_3$) δ 161.3, 144.9, 127.7, 127.2, 122.6, 121.8, 115.4, 55.9, 49.7, 31.3, 29.5, 26.3, 22.6, 14.1, 12.0.
Melting point: 59 °C

Elemental analysis $C_{17}H_{25}BrN_2O$ calc.: C: 57.74%, H: 7.13%, N: 7.93%.
found: C: 57.43%, H: 7.42%, N: 7.87%.

1-(4-methoxyphenyl)-2-methyl-3-octyl-1*H*-imidazol-3-ium bromide [51]

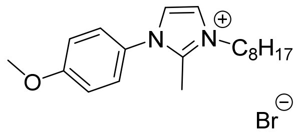

In an Ace pressure tube 1-(4-methoxyphenyl)-2-methyl-1*H*-imidazole **19** (1.88 g, 10 mmol) and 1-bromooctane (2.12 g, 11 mmol) were dissolved in acetonitrile (5 mL). The reaction was stirred for 24 h at 90 °C. When the reaction was complete, the solvent was evaporated and the residue dissolved in a small amount of DCM. The solution was then poured into a large excess of diethyl ether. The precipitate formed was filtered off and washed with diethyl ether. After drying *in vacuo* the product was obtained as a white solid (3.70 g, 9.7 mmol, 97 %).

^{1}H NMR (300 MHz, $CDCl_3$) δ 7.70 (d, *J* = 2.1 Hz, 1H), 7.52 (d, *J* = 8.9 Hz, 2H), 7.32 (d, *J* = 2.1 Hz, 1H), 7.01 (d, *J* = 9.0 Hz, 2H), 4.31 (t, *J* = 7.6 Hz, 2H), 3.84 (s, 3H), 2.69 (s, 3H), 1.90 (quint, *J* = 7.5 Hz, 2H), 1.43 – 1.17 (m, 10H), 0.84 (t, *J* = 6.7 Hz, 3H).

^{13}C NMR (75 MHz, $CDCl_3$) δ 161.3, 144.7, 127.6, 127.1, 122.6, 122.0, 115.4, 55.7, 49.6, 31.8, 29.6, 29.1, 26.6, 22.6, 14.1, 11.8.

Melting point: 72 °C

Elemental analysis $C_{19}H_{29}BrN_2O$ calc.: C: 59.84%, H: 7.66%, N: 7.35%.
found: C: 59.56%, H: 7.60%, N: 7.02%.

1-(4-methoxyphenyl)-2-methyl-3-undecyl-1*H*-imidazol-3-ium bromide [52]

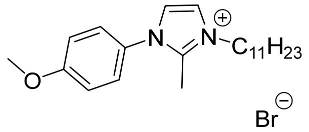

In an Ace pressure tube 1-(4-methoxyphenyl)-2-methyl-1*H*-imidazole **19** (1.88 g, 10 mmol) and 1-bromoundecane (2.64 g, 11 mmol) were dissolved in acetonitrile (5 mL). The reaction was stirred for 24 h at 90 °C. When the reaction was complete, the solvent was evaporated and the residue dissolved in a small amount of DCM. The solution was then poured into a large excess of diethyl ether. The precipitate formed was filtered off and washed with diethyl ether. After drying *in vacuo* the product was obtained as a yellow solid (4.04 g, 9.5 mmol, 95 %).

^{1}H NMR (300 MHz, $CDCl_3$) δ 7.67 (d, *J* = 2.1 Hz, 1H), 7.54 (d, *J* = 9.0 Hz, 2H), 7.31 (d, *J* = 2.1 Hz, 1H), 7.03 (d, *J* = 9.0 Hz, 2H), 4.33 (t, *J* = 7.6 Hz, 2H), 3.85 (s, 3H), 2.70 (s, 3H), 1.91 (quint, *J* = 7.4 Hz, 2H), 1.42 – 1.20 (m, 16H), 0.86 (t, *J* = 6.7 Hz, 3H).

^{13}C NMR (75 MHz, $CDCl_3$) δ 161.3, 144.8, 127.7, 127.2, 122.6, 121.9, 115.5, 55.9, 49.7, 32.0, 29.7, 29.6, 29.5, 29.4, 29.2, 26.7, 22.8, 14.2, 11.9.

Melting point: 78 °C

Elemental analysis $C_{22}H_{35}BrN_2O$ calc.: C: 62.40%, H: 8.33%, N: 6.62%.
found: C: 62.06%, H: 8.25%, N: 6.84%.

3-butyl-1-(2,4-difluorophenyl)-2-methyl-1*H*-imidazol-3-ium bromide [53]

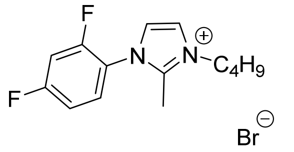

In an Ace pressure tube 1-(2,4-difluorophenyl)-2-methyl-1*H*-imidazole **20** (2.91 g, 15 mmol) and 1-bromobutane (2.31 g, 16.5 mmol) were dissolved in acetonitrile (5 mL). The reaction was stirred for 24 h at 90 °C. When the reaction was complete, the solvent was evaporated and the residue dissolved in a small amount of DCM. The solution was then poured into a large excess of diethyl ether. The precipitate formed was filtered off and washed with diethyl ether. After drying *in vacuo* the product was obtained as a white solid (4.85 g, 14.6 mmol, 98 %).

^{1}H NMR (300 MHz, $CDCl_3$) δ 8.16 (td, *J* = 8.7, 5.6 Hz, 1H), 7.85 (d, *J* = 2.2 Hz, 1H), 7.40 (d, *J* = 2.1 Hz, 1H), 7.14 – 6.95 (m, 2H), 4.31 (t, *J* = 7.6 Hz, 2H), 2.64 (s, 3H), 1.97 – 1.77 (m, 2H), 1.40 (sext, *J* = 7.5 Hz, 2H), 0.93 (t, *J* = 7.3 Hz, 3H).

^{13}C NMR (75 MHz, $CDCl_3$) δ 164.0 (dd, *J* = 256.4, 11.1 Hz), 156.6 (dd, *J* = 256.1, 12.7 Hz), 145.6, 131.4 (d, *J* = 10.3 Hz), 122.9, 122.6, 118.4 (dd, *J* = 12.3, 4.2 Hz), 113.5 (dd, *J* = 22.8, 3.9 Hz), 105.6 (dd, *J* = 26.9, 22.6 Hz), 49.5, 31.3, 19.7, 13.6, 11.7 (d, *J* = 1.4 Hz).

Melting point: 151 °C

Elemental analysis $C_{14}H_{17}BrF_2N_2 \cdot 0.15\ H_2O$ calc.: C: 50.36%, H: 5.22%, N: 8.39%.
found: C: 50.17%, H: 4.92%, N: 8.60%.

1-(2,4-difluorophenyl)-3-hexyl-2-methyl-1*H*-imidazol-3-ium bromide [54]

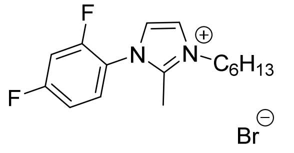

In an Ace pressure tube 1-(2,4-difluorophenyl)-2-methyl-1*H*-imidazole **20** (2.91 g, 15 mmol) and 1-bromohexane (2.75 g, 16.5 mmol) were dissolved in acetonitrile (5 mL). The reaction was stirred for 24 h at 90 °C. When the reaction was complete, the solvent was evaporated and the residue dissolved in a small amount of DCM. The solution was then poured into a large excess of diethyl ether. The precipitate formed was filtered off and washed with diethyl ether. After drying *in vacuo* the product was obtained as a white solid (5.08 g, 14.1 mmol, 94 %).

^{1}H NMR (300 MHz, $CDCl_3$) δ 8.26 (td, *J* = 8.7, 5.7 Hz, 1H), 7.78 (d, *J* = 2.1 Hz, 1H), 7.38 (d, *J* = 1.9 Hz, 1H), 7.16 – 7.00 (m, 2H), 4.31 (t, *J* = 7.7 Hz, 2H), 2.67 (s, 3H), 1.93 (quint, *J* = 7.5 Hz, 2H), 1.46 – 1.20 (m, 6H), 0.85 (t, *J* = 7.0 Hz, 3H).

^{13}C NMR (75 MHz, $CDCl_3$) δ 164.1 (dd, *J* = 256.4, 11.0 Hz), 156.7 (dd, *J* = 255.9, 12.7 Hz), 145.8, 131.7 (d, *J* = 10.3 Hz), δ 123.0, 122.5, 118.6 (dd, *J* = 12.3, 4.2 Hz), 113.6 (dd, *J* = 22.7, 3.9 Hz), 105.6 (dd, *J* = 27.0, 22.5 Hz), 49.8, 31.2, 29.4, 26.2, 22.5, 14.0, 11.8 (d, *J* = 1.2 Hz).

Melting point: 81 °C

Elemental analysis $C_{16}H_{21}BrF_2N_2$ calc.: C: 53.49%, H: 5.89%, N: 7.80%.
found: C: 53.28%, H: 5.87%, N: 7.93%.

1-(2,4-difluorophenyl)-2-methyl-3-octyl-1*H*-imidazol-3-ium bromide [55]

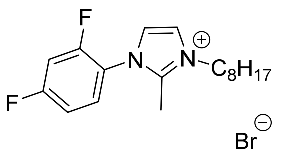

In an Ace pressure tube 1-(2,4-difluorophenyl)-2-methyl-1*H*-imidazole **20** (2.91 g, 15 mmol) and 1-bromooctane (3.19 g, 16.5 mmol) were dissolved in acetonitrile (5 mL). The reaction was stirred for 24 h at 90 °C. When the reaction was complete, the solvent was evaporated and the residue dissolved in a small amount of DCM. The solution was then poured into a large excess of diethyl ether. The precipitate formed was filtered off and washed with diethyl ether. After drying *in vacuo* the product was obtained as a white solid (5.61 g, 14.5 mmol, 97 %).

^{1}H NMR (300 MHz, $CDCl_3$) δ 8.25 (td, *J* = 8.7, 5.6 Hz, 1H), 7.79 (d, *J* = 2.2 Hz, 1H), 7.39 (d, *J* = 2.1 Hz, 1H), 7.17 – 6.99 (m, 2H), 4.31 (t, *J* = 7.6 Hz, 2H), 2.66 (s, 3H), 1.92 (quint, *J* = 7.4 Hz, 2H), 1.45 – 1.13 (m, 10H), 0.83 (t, *J* = 6.7 Hz, 3H).

^{13}C NMR (75 MHz, $CDCl_3$) δ 164.1 (dd, *J* = 256.5, 11.0 Hz), 156.7 (dd, *J* = 255.9, 12.7 Hz), 145.7, 131.6 (d, *J* = 10.3 Hz), 123.0, 122.5, 118.5 (dd, *J* = 12.3, 4.1 Hz), 113.6 (dd, *J* = 22.7, 3.9 Hz), 105.6 (dd, *J* = 26.9, 22.5 Hz), 49.8, 31.7, 29.4, 29.1, 29.1, 26.5, 22.6, 14.1, 11.78 (d, *J* = 1.3 Hz).

Melting point: 82 °C

Elemental analysis $C_{18}H_{25}BrF_2N_2$ calc.: C: 55.82%, H: 6.51%, N: 7.23%.

found: C: 55.38%, H: 6.33%, N: 7.44%.

1-(2,4-difluorophenyl)-2-methyl-3-undecyl-1*H*-imidazol-3-ium bromide [56]

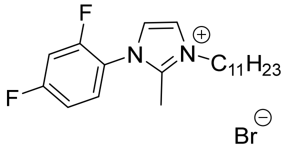

In an Ace pressure tube 1-(2,4-difluorophenyl)-2-methyl-1*H*-imidazole **20** (2.91 g, 15 mmol) and 1-bromoundecane (3.96 g, 16.5 mmol) were dissolved in acetonitrile (5 mL). The reaction was stirred for 24 h at 90 °C. When the reaction was complete, the solvent was evaporated and the residue dissolved in a small amount of DCM. The solution was then poured into a large excess of diethyl ether. The precipitate formed was filtered off and washed with diethyl ether. After drying *in vacuo* the product was obtained as a brown glassy solid (5.93 g, 13.8 mmol, 92 %).

^{1}H NMR (300 MHz, $CDCl_3$) δ 8.23 (dd, *J* = 14.2, 8.5 Hz, 1H), 7.79 (s, 1H), 7.39 (s, 1H), 7.16 – 6.96 (m, 2H), 4.30 (t, *J* = 7.4 Hz, 2H), 2.65 (s, 3H), 1.99 – 1.82 (m, 2H), 1.44 – 1.07 (m, 16H), 0.82 (t, *J* = 6.6 Hz, 3H).

^{13}C NMR (75 MHz, $CDCl_3$) δ 164.0 (dd, *J* = 256.3, 11.0 Hz), 156.6 (dd, *J* = 255.9, 12.7 Hz), 145.7, 131.6 (d, *J* = 10.3 Hz), 123.0, 122.5, 118.5 (dd, *J* = 12.4, 4.2 Hz), 113.6 (dd, *J* = 22.7, 3.9 Hz), 105.5 (dd, *J* = 27.0, 22.6 Hz), 49.8, 31.9, 29.5, 29.5, 29.4, 29.4, 29.3, 29.1, 26.5, 22.7, 14.1, 11.8 (d, *J* = 1.1 Hz).

Melting point: amorphous

Elemental analysis $C_{21}H_{31}BrF_2N_2 \cdot 1\ H_2O$ calc.: C: 56.38%, H: 7.43%, N: 6.26%.

found: C: 56.21%, H: 7.49%, N: 6.12%.

3-butyl-1-phenyl-2-propyl-1*H*-imidazol-3-ium bromide [57]

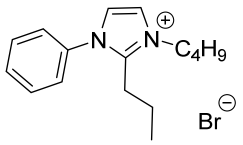

In an Ace pressure tube 1-phenyl-2-propyl-1*H*-imidazole **21** (5.59 g, 30 mmol) and 1-bromobutane (4.61 g, 33 mmol) were dissolved in acetonitrile (5 mL). The reaction was stirred for 24 h at 90 °C. When the reaction was complete, the solvent was evaporated and the residue dissolved in a small amount of DCM. The solution was then poured into a large excess of diethyl ether. The precipitate formed was filtered off and washed with diethyl ether. After drying *in vacuo* the product was obtained as a grey solid (9.16 g, 28.3 mmol, 94 %).

^{1}H NMR (300 MHz, $CDCl_3$) δ 7.92 (d, *J* = 2.1 Hz, 1H), 7.59 – 7.53 (m, 5H), 7.36 (d, *J* = 2.1 Hz, 1H), 4.33 (t, *J* = 7.7 Hz, 2H), 3.01 (t, *J* = 7.9 Hz, 2H), 1.99 – 1.86 (m, 2H), 1.58 – 1.33 (m, 4H), 0.96 (t, *J* = 7.3 Hz, 3H), 0.81 (t, *J* = 7.4 Hz, 3H).

^{13}C NMR (75 MHz, $CDCl_3$) δ 147.3, 134.6, 131.1, 130.4, 126.3, 122.9, 122.4, 49.1, 31.9, 26.3, 21.1, 19.8, 13.8, 13.6.

Melting point: 139 °C

Elemental analysis $C_{16}H_{23}BrN_2$ calc.: C: 59.45%, H: 7.17%, N: 8.67%.
found: C: 59.17%, H: 7.15%, N: 8.67%.

3-hexyl-1-phenyl-2-propyl-1*H*-imidazol-3-ium bromide [58]

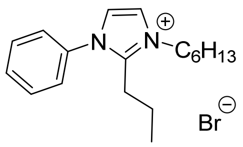

In an Ace pressure tube 1-phenyl-2-propyl-1*H*-imidazole **21** (6.5 g, 34.9 mmol) and 1-bromohexane (6.4 g, 38.4 mmol) were dissolved in tetrahydrofuran (5 mL). The reaction was stirred for 24 h at 90 °C. When the reaction was complete, the solvent was evaporated and the residue dissolved in a small amount of DCM. The solution was then poured into a large excess of diethyl ether. The precipitate formed was filtered off and washed with diethyl ether. After drying *in vacuo* the product was obtained as a white solid (7.26 g, 20.37mmol, 59 %).

^{1}H NMR (300 MHz, $CDCl_3$) δ 7.86 (d, *J* = 2.1 Hz, 1H), 7.65 – 7.55 (m, 5H), 7.35 (d, *J* = 2.1 Hz, 1H), 4.34 (t, *J* = 7.8 Hz, 2H), 3.05 (t, *J* = 8.1 Hz, 2H), 1.96 (quint, *J* = 7.7 Hz, 2H), 1.53 – 1.40 (m, 4H), 1.38 – 1.27 (m, 4H), 0.88 (t, *J* = 7.1 Hz, 3H), 0.84 (t, *J* = 7.4 Hz, 3H).

^{13}C NMR (75 MHz, $CDCl_3$) δ 147.7, 134.8, 131.2, 130.5, 126.5, 122.8, 122.3, 49.4, 31.3, 30.0, 26.6, 26.3, 22.5, 21.2, 14.1, 13.9.

Melting point: 48 °C

Elemental analysis $C_{18}H_{27}BrN_2$ calc.: C: 61.54%, H: 7.75%, N: 7.97%.
found: C: 61.28%, H: 7.90%, N: 7.99%.

3-octyl-1-phenyl-2-propyl-1*H*-imidazol-3-ium bromide [59]

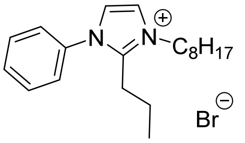

In an Ace pressure tube 1-phenyl-2-propyl-1*H*-imidazole **21** (5.59 g, 30 mmol) and 1-bromooctane (6.37 g, 33 mmol) were dissolved in acetonitrile (5 mL). The reaction was stirred for 24 h at 90 °C. When the reaction was complete, the solvent was evaporated and the residue dissolved in a small amount of DCM. The solution was then poured into a large excess of diethyl ether. The precipitate formed was filtered off and washed with diethyl ether. After drying *in vacuo* the product was obtained as a white solid (10.4 g, 27.3 mmol, 91 %).

^{1}H NMR (300 MHz, $CDCl_3$) δ 7.88 (d, *J* = 2.1 Hz, 1H), 7.60 – 7.52 (m, 5H), 7.36 (d, *J* = 2.1 Hz, 1H), 4.31 (t, *J* = 7.8 Hz, 2H), 3.00 (t, *J* = 8.0 Hz, 2H), 1.93 (quint, *J* = 7.5 Hz, 2H), 1.53 – 1.19 (m, 12H), 0.86 – 0.76 (m, 6H).

^{13}C NMR (75 MHz, $CDCl_3$) δ 147.4, 134.6, 131.1, 130.4, 126.3, 122.9, 122.3, 49.3, 31.7, 30.0, 29.0, 26.5, 26.3, 22.6, 21.1, 14.1, 13.8.

Melting point: 88 °C

Elemental analysis $C_{20}H_{31}BrN_2$ calc.: C: 63.32%, H: 8.24%, N: 7.38%.
found: C: 63.09%, H: 8.41%, N: 7.49%.

1-phenyl-2-propyl-3-undecyl-1*H*-imidazol-3-ium bromide [60]

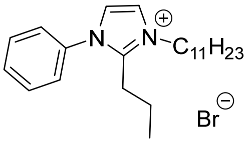

In an Ace pressure tube 1-phenyl-2-propyl-1*H*-imidazole **21** (5.59 g, 30 mmol) and 1-bromoundecane (7.92 g, 33 mmol) were dissolved in acetonitrile (5 mL). The reaction was stirred for 24 h at 90 °C. When the reaction was complete, the solvent was evaporated and the residue dissolved in a small amount of DCM. The solution was then poured into a large excess of diethyl ether. The precipitate formed was filtered off and washed with diethyl ether. After drying *in vacuo* the product was obtained as a yellow solid (11.2 g, 26.5 mmol, 88 %).

^{1}H NMR (300 MHz, $CDCl_3$) δ 7.87 (d, *J* = 2.0 Hz, 1H), 7.61 – 7.51 (m, 5H), 7.36 (d, *J* = 2.0 Hz, 1H), 4.31 (t, *J* = 7.7 Hz, 2H), 3.01 (t, *J* = 8.0 Hz, 2H), 1.93 (quint, *J* = 7.3 Hz, 2H), 1.52 – 1.18 (m, 18H), 0.88 – 0.76 (m, 6H).

^{13}C NMR (75 MHz, $CDCl_3$) δ 147.6, 134.7, 131.2, 130.5, 126.5, 122.9, 122.3, 49.4, 31.9, 30.1, 29.6, 29.6, 29.5, 29.4, 29.2, 26.7, 26.5, 22.7, 21.2, 14.2, 13.9.

Melting point: 72 °C

Elemental analysis $C_{23}H_{37}BrN_2$ calc.: C: 65.55%, H: 8.85%, N: 6.65%.
found: C: 65.25%, H: 8.85%, N: 6.80%.

3-butyl-1-(2-methylphenyl)-2-propyl-1*H*-imidazol-3-ium bromide [61]

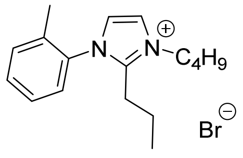

In an Ace pressure tube 1-(2-methylphenyl)-2-propyl-1*H*-imidazole **22** (3 g, 15 mmol) and 1-bromobutane (2.31 g, 16.5 mmol) were dissolved in acetonitrile (5 mL). The reaction was stirred for 24 h at 90 °C. When the reaction was complete, the solvent was evaporated and the residue dissolved in a small amount of DCM. The solution was then poured into a large excess of diethyl ether. The precipitate formed was filtered off and washed with diethyl ether. After drying *in vacuo* the product was obtained as a white solid (4.88 g, 14.5 mmol, 97 %).

^{1}H NMR (500 MHz, $CDCl_3$) δ 8.10 (d, *J* = 2.1 Hz, 1H), 7.46 – 7.42 (m, 2H), 7.37 – 7.31 (m, 2H), 7.27 (d, *J* = 2.1 Hz, 1H), 4.46 – 4.30 (m, 2H), 3.01 –2.66 (m, 2H), 2.04 (s, 3H), 1.94 – 1.86 (m, 2H), 1.41 (sext, *J* = 7.7 Hz, 4H), 0.92 (t, *J* = 7.4 Hz, 3H), 0.79 (t, *J* = 7.4 Hz, 3H).

^{13}C NMR (75 MHz, $CDCl_3$) δ 147.0, 134.2, 134.2, 133.1, 131.8, 131.4, 127.8, 127.2, 122.9, 122.6, 49.0, 31.9, 25.8, 20.7, 19.6, 17.2, 17.2, 13.7, 13.6, 13.5, 13.4.

Melting point: 94 °C

Elemental analysis $C_{17}H_{25}BrN_2$ calc.: C: 60.54%, H: 7.47%, N: 8.31%.
found: C: 60.755%, H: 7.66%, N: 8.33%.

3-hexyl-1-(2-methylphenyl)-2-propyl-1*H*-imidazol-3-ium bromide [62]

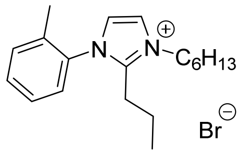

In an Ace pressure tube 1-(2-methylphenyl)-2-propyl-1*H*-imidazole **22** (3 g, 15 mmol) and 1-bromohexane (2.75 g, 16.5 mmol) were dissolved in acetonitrile (5 mL). The reaction was stirred for 24 h at 90 °C. When the reaction was complete, the solvent was evaporated and the residue dissolved in a small amount of DCM. The solution was then poured into a large excess of diethyl ether. The precipitate formed was filtered off and washed with diethyl ether. After drying *in vacuo* the product was obtained as a yellow liquid (4.49 g, 12.3 mmol, 82 %).

^{1}H NMR (300 MHz, $CDCl_3$) δ 8.10 (d, *J* = 2.0 Hz, 1H), 7.53 – 7.42 (m, 2H), 7.41 – 7.33 (m, 2H), 7.32 (d, *J* = 2.0 Hz, 1H), 4.46 – 4.26 (m, 2H), 2.86 (ddt, *J* = 76.8, 15.6, 7.8 Hz, 2H), 2.07 (s, 3H), 1.94 (quint, *J* = 7.7 Hz, 2H), 1.51 – 1.21 (m, 8H), 0.81 (t, *J* = 7.2 Hz, 6H).

^{13}C NMR (75 MHz, $CDCl_3$) δ 146.9, 134.1, 133.0, 131.7, 131.3, 127.7, 127.1, 122.8, 122.6, 49.1, 30.9, 29.9, 25.8, 25.7, 22.1, 20.6, 17.8, 13.7, 13.6.

Melting point: liquid at room temperature

Elemental analysis $C_{19}H_{29}BrN_2$ calc.: C: 62.32%, H: 8.00%, N: 7.67%.
found: C: 62.25%, H: 8.29%, N: 7.91%.

1-(2-methylphenyl)-3-octyl-2-propyl-1*H*-imidazol-3-ium bromide [63]

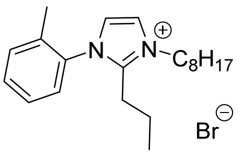

In an Ace pressure tube 1-(2-methylphenyl)-2-propyl-1*H*-imidazole **22** (3 g, 15 mmol) and 1-bromooctane (3.19 g, 16.5 mmol) were dissolved in acetonitrile (5 mL). The reaction was stirred for 24 h at 90 °C. When the reaction was complete, the solvent was evaporated and the residue dissolved in a small amount of DCM. The solution was then poured into a large excess of diethyl ether. The precipitate formed was filtered off and washed with diethyl ether. After drying *in vacuo* the product was obtained as a yellow liquid (4.98 g, 12.7 mmol, 84 %).

^{1}H NMR (500 MHz, $CDCl_3$) δ 8.02 (d, *J* = 2.1 Hz, 1H), 7.43 – 7.38 (m, 2H), 7.33 – 7.26 (m, 2H), 7.25 (d, *J* = 2.1 Hz, 1H), 4.41 – 4.24 (m, 2H), 2.95 – 2.64 (m, 2H), 2.00 (s, 3H), 1.91 – 1.82 (m, 2H), 1.40 – 1.09 (m, 12H), 0.77 – 0.71 (m, 6H).

^{13}C NMR (75 MHz, $CDCl_3$) δ 147.1, 134.3, 133.2, 131.9, 131.4, 127.8, 127.3, 122.9, 122.7, 49.3, 31.7, 30.1, 29.4, 29.3, 29.2, 29.1, 29.0, 26.3, 25.9, 22.5, 20.7, 17.3, 13.9, 13.7.

Melting point: liquid at room temperature

Elemental analysis $C_{21}H_{33}BrN_2$ calc.: C: 64.11%, H: 8.45%, N: 7.12%.
found: C: 63.87%, H: 8.62%, N: 7.37%.

1-(2-methylphenyl) -2-propyl-3-undecyl-1*H*-imidazol-3-ium bromide [64]

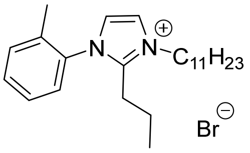

In an Ace pressure tube 1-(2-methylphenyl)-2-propyl-1*H*-imidazole **22** (3 g, 15 mmol) and 1-bromoundecane (3.96 g, 16.5 mmol) were dissolved in acetonitrile (5 mL). The reaction was stirred for 24 h at 90 °C. When the reaction was complete, the solvent was evaporated and the residue dissolved in a small amount of DCM. The solution was then poured into a large excess of diethyl ether. The precipitate formed was filtered off and washed with diethyl ether. After drying *in vacuo* the product was obtained as a yellow liquid (6.21 g, 14.3 mmol, 95 %).

^{1}H NMR (300 MHz, $CDCl_3$) δ 8.04 (d, *J* = 1.9 Hz, 1H), 7.45 (t, *J* = 7.7 Hz, 2H), 7.39 – 7.31 (m, 2H), 7.29 (d, *J* = 1.8 Hz, 1H), 4.42 – 4.30 (m, 2H), 2.83 (ddt, *J* = 74.2, 15.5, 7.6 Hz, 2H), 2.06 (s, 3H), 1.99 – 1.83 (m, 2H), 1.48 – 1.14 (m, 18H), 0.84 – 0.75 (m, 6H).

^{13}C NMR (75 MHz, $CDCl_3$) δ 147.2, 134.4, 133.3, 131.9, 131.5, 127.9, 127.5, 122.9, 122.8, 49.4, 31.8, 30.1, 29.5, 29.4, 29.3, 29.2, 29.1, 26.4, 26.0, 22.6, 20.8, 17.4, 14.0, 13.8.

Melting point: liquid at room temperature

Elemental analysis $C_{24}H_{39}BrN_2$ calc.: C: 66.19%, H: 9.03%, N: 6.43%.
found: C: 65.87%, H: 9.03%, N: 6.56%.

3-butyl-1-(4-methyoxyphenyl)-2-propyl-1*H*-imidazol-3-ium bromide [65]

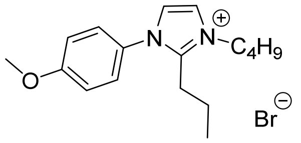

In an Ace pressure tube 1-(4-methoxyphenyl)-2-propyl-1*H*-imidazole **23** (2.16 g, 10 mmol) and 1-bromobutane (1.54 g, 11 mmol) were dissolved in acetonitrile (5 mL). The reaction was stirred for 24 h at 90 °C. When the reaction was complete, the solvent was evaporated and the residue dissolved in a small amount of DCM. The solution was then poured into a large excess of diethyl ether. The precipitate formed was filtered off and washed with diethyl ether. After drying *in vacuo* the product was obtained as a white solid (3.2 g, 9.1 mmol, 91 %).

^{1}H NMR (300 MHz, $CDCl_3$) δ 7.87 (d, *J* = 2.1 Hz, 1H), 7.50 (d, *J* = 9.0 Hz, 2H), 7.31 (d, *J* = 2.1 Hz, 1H), 7.03 (d, *J* = 9.0 Hz, 2H), 4.33 (t, *J* = 7.7 Hz, 2H), 3.85 (s, 3H), 3.00 (t, *J* = 8.0 Hz, 2H), 1.56 – 1.40 (m, 4H), 0.98 (t, *J* = 7.3 Hz, 3H), 0.84 (t, *J* = 7.4 Hz, 3H).

^{13}C NMR (75 MHz, $CDCl_3$) δ 161.3, 147.7, 127.7, 127.1, 123.2, 122.2, 115.5, 55.9, 49.1, 32.0, 26.3, 21.2, 19.9, 13.9, 13.7.

Melting point: 158 °C

Elemental analysis $C_{17}H_{25}BrN_2O$ calc.: C: 57.79%, H: 7.13%, N: 7.93%.
found: C: 57.46%, H: 7.13%, N: 7.86%.

3-hexyl-1-(4-methyoxyphenyl)-2-propyl-1*H*-imidazol-3-ium bromide [66]

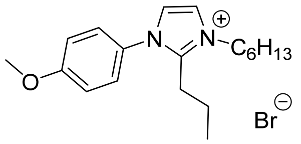

In an Ace pressure tube 1-(4-methoxyphenyl)-2-propyl-1*H*-imidazole **23** (2.16 g, 10 mmol) and 1-bromohexane (1.83 g, 11 mmol) were dissolved in acetonitrile (5 mL). The reaction was stirred for 24 h at 90 °C. When the reaction was complete, the solvent was evaporated and the residue dissolved in a small amount of DCM. The solution was then poured into a large excess of diethyl ether. The precipitate formed was filtered off and washed with diethyl ether. After drying *in vacuo* the product was obtained as a white solid (3.4 g, 9.0 mmol, 90 %).

^{1}H NMR (300 MHz, $CDCl_3$) δ 7.81 (d, *J* = 2.1 Hz, 1H), 7.53 (d, *J* = 8.9 Hz, 2H), 7.31 (d, *J* = 2.1 Hz, 1H), 7.04 (d, *J* = 8.9 Hz, 2H), 4.33 (t, *J* = 7.8 Hz, 2H), 3.87 (s, 3H), 3.01 (t, *J* = 8.0 Hz, 2H), 1.96 (quint, *J* = 7.6 Hz, 2H), 1.57 – 1.30 (m, 8H), 0.87 (q, *J* = 7.2 Hz, 6H).

^{13}C NMR (75 MHz, $CDCl_3$) δ 161.4, 147.8, 127.8, 127.2, 123.2, 122.1, 115.5, 55.9, 49.4, 31.3, 30.0, 26.4, 26.4, 22.6, 21.2, 14.1, 14,0.

Melting point: 121 °C

Elemental analysis $C_{19}H_{29}BrN_2O$ calc.: C: 59.84%, H: 7.66%, N: 7.45%.
found: C: 59.79%, H: 7.69%, N: 7.37%.

1-(4-methyoxyphenyl)-3-octyl-2-propyl-1*H*-imidazol-3-ium bromide [67]

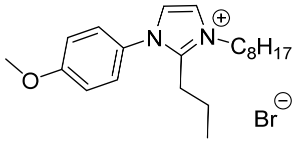

In an Ace pressure tube 1-(4-methoxyphenyl)-2-propyl-1*H*-imidazole **23** (2.16 g, 10 mmol) and 1-bromooctane (2.12 g, 11 mmol) were dissolved in acetonitrile (5 mL). The reaction was stirred for 24 h at 90 °C. When the reaction was complete, the solvent was evaporated and the residue dissolved in a small amount of DCM. The solution was then poured into a large excess of diethyl ether. The precipitate formed was filtered off and washed with diethyl ether. After drying *in vacuo* the product was obtained as a white solid (3.64 g, 8.9 mmol, 89 %).

^{1}H NMR (300 MHz, $CDCl_3$) δ 7.81 (d, *J* = 2.1 Hz, 1H), 7.52 (d, *J* = 8.9 Hz, 2H), 7.31 (d, *J* = 2.1 Hz, 1H), 7.03 (d, *J* = 8.9 Hz, 2H), 4.32 (t, *J* = 7.7 Hz, 2H), 3.87 (s, 3H), 3.01 (t, *J* = 8.0 Hz, 2H), 1.95 (quint, *J* = 7.7 Hz, 2H), 1.54 – 1.23 (m, 12H), 0.91 – 0.81 (m, 6H).

^{13}C NMR (75 MHz, $CDCl_3$) δ 161.4, 147.7, 127.8, 127.2, 123.2, 122.1, 115.5, 55.9, 49.4, 31.8, 30.1, 29.2, 26.7, 26.4, 22.7, 21.2, 14.2, 13.9.

Melting point: 84 °C

Elemental analysis $C_{21}H_{33}BrN_2O$ calc.: C: 61.61%, H: 8.12%, N: 6.84%.
found: C: 61.50%, H: 8.11%, N: 6.86%.

1-(4-methyoxyphenyl)-2-propyl-3-undecyl-1*H*-imidazol-3-ium bromide [68]

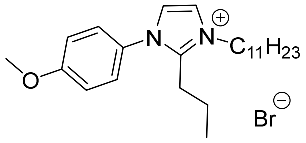

In an Ace pressure tube 1-(4-methoxyphenyl)-2-propyl-1*H*-imidazole **23** (2.16 g, 10 mmol) and 1-bromoundecane (2.64 g, 11 mmol) were dissolved in acetonitrile (5 mL). The reaction was stirred for 24 h at 90 °C. When the reaction was complete, the solvent was evaporated and the residue dissolved in a small amount of DCM. The solution was then poured into a large excess of diethyl ether. The precipitate formed was filtered off and washed with diethyl ether. After drying *in vacuo* the product was obtained as a white solid (3.87 g, 8.6 mmol, 86 %).

^{1}H NMR (300 MHz, $CDCl_3$) δ 7.80 (d, *J* = 2.1 Hz, 1H), 7.52 (d, *J* = 8.9 Hz, 2H), 7.31 (d, *J* = 2.1 Hz, 1H), 7.03 (d, *J* = 8.9 Hz, 2H), 4.31 (t, *J* = 7.7 Hz, 2H), 3.86 (s, 3H), 3.00 (t, *J* = 8.0 Hz, 2H), 1.95 (quint, *J* = 7.5 Hz, 2H), 1.54 – 1.21 (m, 18H), 0.90 – 0.81 (m, 6H).

^{13}C NMR (75 MHz, $CDCl_3$) δ 161.3, 147.7, 127.8, 127.2, 123.2, 122.1, 115.5, 55.9, 53.5, 49.4, 32.0, 30.1, 29.6, 29.6, 29.5, 29.4, 29.2, 26.7, 26.4, 22.8, 21.2, 14.2, 13.9.

Melting point: 92 °C

Elemental analysis $C_{24}H_{39}BrN_2O$ calc.: C: 63.85%, H: 8.71%, N: 6.20%.
found: C: 63.45%, H: 8.61%, N: 6.01%.

3-butyl-1-(2,4-difluorophenyl)-2-propyl-1*H*-imidazol-3-ium bromide [69]

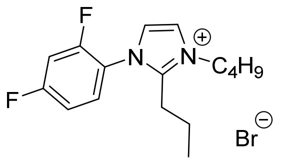

In an Ace pressure tube 1-(2,4-difluorophenyl)-2-propyl-1*H*-imidazole **24** (3.33 g, 15 mmol) and 1-bromobutane (2.31 g, 16.5 mmol) were dissolved in acetonitrile (5 mL). The reaction was stirred for 24 h at 90 °C. When the reaction was complete, the solvent was evaporated and the residue dissolved in a small amount of DCM. The solution was then poured into a large excess of diethyl ether. The precipitate formed was filtered off and washed with diethyl ether. After drying *in vacuo* the product was obtained as a white solid (5.22 g, 14.5 mmol, 97 %).

^{1}H NMR (300 MHz, $CDCl_3$) δ 8.33 (td, *J* = 8.7, 5.6 Hz, 1H), 7.85 (d, *J* = 2.2 Hz, 1H), 7.37 (d, *J* = 2.1 Hz, 1H), 7.19 – 6.98 (m, 2H), 4.30 (t, *J* = 7.8 Hz, 2H), 2.98 (t, *J* = 8.0 Hz, 2H), 1.96 (quint, *J* = 7.7 Hz, 2H), 1.57 – 1.37 (m, 4H), 0.98 (t, *J* = 7.3 Hz, 3H), 0.84 (t, *J* = 7.4 Hz, 3H).

^{13}C NMR (75 MHz, $CDCl_3$) δ 164.1 (dd, *J* = 256.6, 10.9 Hz), 157.0 (dd, *J* = 256.0, 12.7 Hz), 148.9, 131.72 (d, *J* = 10.2 Hz), 123.4, 122.4, 118.7 (dd, *J* = 12.5, 4.2 Hz), 113.6 (dd, *J* = 22.7, 3.9 Hz), 105.6 (dd, *J* = 26.9, 22.5 Hz) 49.3, 31.7, 26.8, 20.7, 19.9, 13.8, 13.7.

Melting point: 142 °C

Elemental analysis $C_{16}H_{21}BrF_2N_2$ calc.: C: 53.49%, H: 5.89%, N: 7.80%.

found: C: 53.01%, H: 5.88%, N: 7.70%.

1-(2,4-difluorophenyl)-3-hexyl-2-propyl-1*H*-imidazol-3-ium bromide [70]

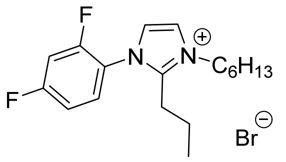

In an Ace pressure tube 1-(2,4-difluorophenyl)-2-propyl-1*H*-imidazole **24** (3.33 g, 15 mmol) and 1-bromohexane (2.75 g, 16.5 mmol) were dissolved in acetonitrile (5 mL). The reaction was stirred for 24 h at 90 °C. When the reaction was complete, the solvent was evaporated and the residue dissolved in a small amount of DCM. The solution was then poured into a large excess of diethyl ether. The precipitate formed was filtered off and washed with diethyl ether. After drying *in vacuo* the product was obtained as a white solid (5.33 g, 13.8 mmol, 92 %).

^{1}H NMR (300 MHz, $CDCl_3$) δ 8.20 (td, *J* = 8.7, 5.6 Hz, 1H), 7.86 (d, *J* = 2.2 Hz, 1H), 7.38 (d, *J* = 2.1 Hz, 1H), 7.15 – 6.94 (m, 2H), 4.25 (t, *J* = 7.8 Hz, 2H), 2.92 (t, *J* = 7.9 Hz, 2H), 1.91 (quint, *J* = 7.6 Hz, 2H), 1.49 – 1.20 (m, 8H), 0.88 – 0.71 (m, *J* = 7.1, 2.0 Hz, 6H).

^{13}C NMR (75 MHz, $CDCl_3$) δ 164.0 (dd, *J* = 256.6, 11.0 Hz), 156.8 (dd, *J* = 256.1, 12.7 Hz), 148.5, 131.4 (d, *J* = 10.3 Hz), 123.4, 122.5, 118.5 (dd, *J* = 12.5, 4.2 Hz), 113.5 (dd, *J* = 22.7, 3.9 Hz), 105.5 (dd, *J* = 26.9, 22.5 Hz), 49.4, 31.1, 29.7, 26.5, 26.1, 22.3, 20.6, 13.8, 13.6.

Melting point: 114 °C

Elemental analysis $C_{18}H_{25}BrF_2N_2$ calc.: C: 55.82%, H: 6.51%, N: 7.23%.

found: C: 55.38%, H: 6.33%, N: 7.44%.

1-(2,4-difluorophenyl)-3-octyl-2-propyl-1*H*-imidazol-3-ium bromide [71]

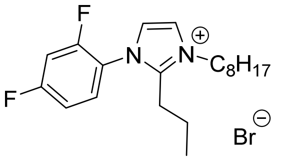

In an Ace pressure tube 1-(2,4-difluorophenyl)-2-propyl-1*H*-imidazole **24** (3.33 g, 15 mmol) and 1-bromooctane (3.19 g, 16.5 mmol) were dissolved in acetonitrile (5 mL). The reaction was stirred for 24 h at 90 °C. When the reaction was complete, the solvent was evaporated and the residue dissolved in a small amount of DCM. The solution was then poured into a large excess of diethyl ether. The precipitate formed was filtered off and washed with diethyl ether. After drying *in vacuo* the product was obtained as a white solid (5.91 g, 14.2 mmol, 95 %).

^{1}H NMR (300 MHz, $CDCl_3$) δ 8.41 (td, *J* = 8.8, 5.7 Hz, 1H), 7.73 (d, *J* = 2.2 Hz, 1H), 7.34 (d, *J* = 2.1 Hz, 1H), 7.23 – 6.98 (m, 2H), 4.29 (t, *J* = 7.8 Hz, 2H), 3.01 (t, *J* = 8.0 Hz, 2H), 1.99 (quint, *J* = 7.6 Hz, 2H), 1.53 – 1.20 (m, 12H), 0.87 (t, *J* = 7.3 Hz, 6H).

^{13}C NMR (75 MHz, $CDCl_3$) δ 164.1 (dd, *J* = 256.5, 11.0 Hz), 156.7 (dd, *J* = 255.9, 12.7 Hz), 149.1, 132.0 (d, *J* = 10.2 Hz), 123.4, 122.1, 118.8 (dd, *J* = 12.4, 4.3 Hz), 113.7 (dd, *J* = 22.6, 3.9 Hz), 105.6 (dd, *J* = 26.7, 22.5 Hz), 49.6, 31.8, 29.8, 29.2, 29.1, 27.0, 26.7, 22.7, 20.7, 14.2, 13.8.

Melting point: 70 °C

Elemental analysis $C_{20}H_{29}BrF_2N_2$ calc.: C: 57.83%, H: 7.04%, N: 6.74%.
found: C: 57.69%, H: 6.96%, N: 6.77%.

1-(2,4-difluorophenyl)-2-propyl-3-undecyl-1*H*-imidazol-3-ium bromide [72]

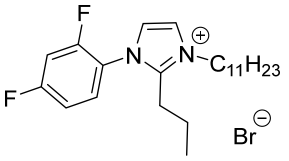

In an Ace pressure tube 1-(2,4-difluorophenyl)-2-propyl-1*H*-imidazole **24** (3.33 g, 15 mmol) and 1-bromoundecane (3.96 g, 16.5 mmol) were dissolved in acetonitrile (5 mL). The reaction was stirred for 24 h at 90 °C. When the reaction was complete, the solvent was evaporated and the residue dissolved in a small amount of DCM. The solution was then poured into a large excess of diethyl ether. The precipitate formed was filtered off and washed with diethyl ether. After drying *in vacuo* the product was obtained as a white solid (6.58 g, 14.4 mmol, 96 %).

^{1}H NMR (300 MHz, $CDCl_3$) δ 8.39 (td, *J* = 8.8, 5.7 Hz, 1H), 7.75 (d, *J* = 2.2 Hz, 1H), 7.35 (d, *J* = 2.1 Hz, 1H), 7.20 – 6.99 (m, 2H), 4.29 (t, *J* = 7.8 Hz, 2H), 3.00 (t, *J* = 8.0 Hz, 2H), 1.98 (quint, *J* = 7.5 Hz, 2H), 1.53 – 1.19 (m, 18H), 0.91 – 0.81 (m, 6H).

^{13}C NMR (75 MHz, $CDCl_3$) δ 164.2 (dd, *J* = 256.7, 11.0 Hz), 157.0 (dd, *J* = 255.9, 12.6 Hz), 149.1, 131.9 (d, *J* = 10.2 Hz), 123.4, 122.2, 118.8 (dd, *J* = 12.3, 4.3 Hz), 113.7 (dd, *J* = 22.7, 3.9 Hz), 105.6 (dd, *J* = 27.0, 22.5 Hz), 49.6, 32.0, 29.8, 29.6, 29.6, 29.5, 29.4, 29.2, 27.0, 26.7, 22.8, 20.7, 14.2, 13.8.

Melting point: 86 °C

Elemental analysis $C_{23}H_{35}BrF_2N_2$ calc.: C: 60.39%, H: 7.71%, N: 6.12%.
found: C: 60.21%, H: 7.70%, N: 6.13%.

5. General synthetic procedure for the synthesis of NTf_2-TAAILs

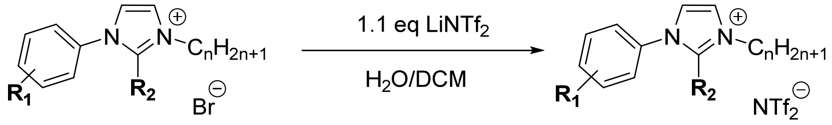

In a round bottom flask, the corresponding bromide salt (1 equiv.) was dissolved in dichloromethane. $LiNTf_2$ (1.1 equiv. 70% aq. solution) and additional water was added to the reaction mixture. The 2-phase system was stirred at room temperature for 24 h. The organic phase was extracted twice with DCM, washed twice with water and dried over $MgSO_4$. The solvent was removed *in vacuo* to yield the corresponding ionic liquid.

3-butyl-1,2-diphenyl-1*H*-imidazol-3-ium bis(trifluoromethylsulfonyl)amide [73]

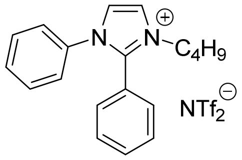

Following the general procedure with bromide salt **25** (62.5 g, 175 mmol) and $LiNTf_2$ (78.9 g, 192 mmol, 70 % aq.) to yield **73** (92.2 g, 165 mmol, 95 %) as a brown liquid.

^{1}H NMR (300 MHz, $CDCl_3$) δ 7.65 (d, J = 2.2 Hz, 1H), 7.61 – 7.27 (m, 11H), 4.13 (t, J = 7.5 Hz, 2H), 1.82 (quint, J = 7.5 Hz, 2H), 1.29 (sext, J = 7.5 Hz, 2H), 0.85 (t, J = 7.3 Hz, 3H).

^{13}C NMR (75 MHz, $CDCl_3$) δ 144.5, 134.6, 132.5, 130.4, 130.4, 129.9, 129.6, 125.8, 123.3, 122.2, 121.9, 120.8, 117.7, 49.1, 31.5, 19.3, 13.0.

Elemental analysis $C_{21}H_{21}F_6N_3O_4S_2$ calc.: C: 45.24%, H: 3.80%, N: 7.54%, S: 11.50%.
found: C: 45.57%, H: 3.71%, N: 7.62%, S: 11.60%.

3-hexyl-1,2-diphenyl-1*H*-imidazol-3-ium bis(trifluoromethylsulfonyl)amide [74]

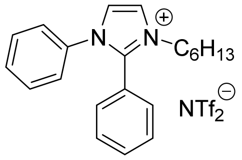

Following general procedure with bromide salt **26** (31.4 g, 81.5 mmol) and $LiNTf_2$ (36.8 g, 89.7 mmol, 70 % aq.) to yield **74** (43.1 g, 73.6 mmol, 90 %) as a brown liquid.

^{1}H NMR (300 MHz, $CDCl_3$) δ 7.57 (d, J = 2.2 Hz, 1H), 7.53 – 7.19 (m, 11H), 4.04 (t, J = 7.7 Hz, 2H), 1.73 (quint, J = 7.7 Hz, 2H), 1.23 – 1.06 (m, 6H), 0.74 (t, J = 6.8 Hz, 3H).

^{13}C NMR (126 MHz, $CDCl_3$) δ 144.6, 134.7, 132.7, 130.6, 130.1, 129.8, 126.0, 123.5, 122.5, 121.3, 121.0, 118.7, 49.5, 30.8, 29.7, 25.9, 22.3, 13.8.

Elemental analysis $C_{23}H_{25}F_6N_3O_4S_2$ calc.: C: 47.18%, H: 4.30%, N: 7.18%, S: 10.95%.
found: C: 47.29%, H: 4.29%, N: 7.55%, S: 10.75%.

3-octyl-1,2-diphenyl-1*H*-imidazol-3-ium bis(trifluoromethylsulfonyl)amide [75]

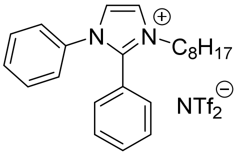

Following general procedure with bromide salt **27** (36.5 g, 88.2 mmol) and $LiNTf_2$ (39.8 g, 22 mmol, 70 % aq.) to yield **75** (53.6 g, 87.4 mmol, 99 %) as a brown liquid.

^{1}H NMR (300 MHz, $CDCl_3$) δ 7.65 – 7.26 (m, 12H), 4.11 (t, *J* = 7.8 Hz, 2H), 1.80 (quint, *J* = 7.5 Hz, 2H), 1.30 – 1.10 (m, 10H), 0.83 (t, *J* = 6.8 Hz, 3H).

^{13}C NMR (75 MHz, $CDCl_3$) δ 144.6, 134.7, 132.7, 130.6, 130.1, 129.8, 125.9, 123.5, 122.5, 122.1, 121.0, 117.9, 49.5, 31.7, 29.8, 28.9, 28.7, 26.3, 22.6, 14.1.

Elemental analysis $C_{25}H_{29}F_6N_3O_4S_2$ calc.: C: 48.93 %, H: 4.76 %, N: 6.85 %, S: 10.45 %.
found: C: 49.21 %, H: 4.53 %, N: 7.12 %, S: 10.09 %.

1,2-diphenyl-3-undecyl-1*H*-imidazol-3-ium bis(trifluoromethylsulfonyl)amide [76]

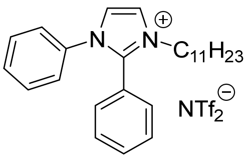

Following general procedure with bromide salt **28** (47.4 g, 104 mmol) and $LiNTf_2$ (47 g, 115 mmol, 70 % aq.) to yield **76** (65.1 g, 99.3 mmol, 96 %) as a brown liquid.

^{1}H NMR (300 MHz, $CDCl_3$) δ 7.66 – 7.26 (m, 12H), 4.12 (t, *J* = 7.7 Hz, 2H), 1.80 (quint, *J* = 7.5 Hz, 2H), 1.30 – 1.15 (m, 16H), 0.86 (t, *J* = 6.8 Hz, 3H).

^{13}C NMR (75 MHz, $CDCl_3$) δ 144.6, 134.7, 132.7, 130.6, 130.1, 129.8, 126.0, 123.6, 122.6, 122.1, 121.0, 117.9, 49.5, 32.0, 29.9, 29.6, 29.5, 29.4, 29.3, 28.8, 26.3, 22.8, 14.2.

Elemental analysis $C_{28}H_{35}F_6N_3O_4S_2$ calc.: C: 51.29%, H: 5.38%, N: 6.41%, S: 9.78%.
found: C: 51.40%, H: 5.91%, N: 6.19%, S: 9.66%.

3-butyl-1-(2-methylphenyl)-2-phenyl-1*H*-imidazol-3-ium bis(trifluoromethylsulfonyl)amide [77]

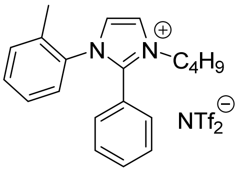

Following general procedure with bromide salt **29** (22.2 g, 60 mmol) and $LiNTf_2$ (27 g, 66 mmol, 70 % aq.) to yield **77** (33.7 g, 59 mmol, 99 %) as a brown liquid.

^{1}H NMR (300 MHz, $CDCl_3$) δ 7.66 (d, *J* = 2.1 Hz, 1H), 7.51 – 7.24 (m, 8H), 7.17 (t, *J* = 6.7 Hz, 2H), 4.11 (t, *J* = 7.7 Hz, 2H), 1.97 (s, 3H), 1.76 (quint, *J* = 7.5 Hz, 2H), 1.22 (sext, *J* = 7.5 Hz, 2H), 0.77 (t, *J* = 7.3 Hz, 3H).

^{13}C NMR (75 MHz, $CDCl_3$) δ 145.1, 134.3, 133.6, 132.8, 131.7, 131.3, 130.1, 129.7, 127.8, 127.7, 123.7, 122.8, 122.1, 120.8, 117.9, 49.40, 31.8, 19.50, 17.3, 13.2.

Elemental analysis $C_{22}H_{23}F_6N_3O_4S_2$ calc.: C: 46.23 %, H: 4.06 %, N: 7.35 %, S: 11.22 %.
found: C: 46.35 %, H: 4.17 %, N: 7.53 %, S: 11.08 %.

3-hexyl-1-(2-methylphenyl)-2-phenyl-1*H*-imidazol-3-ium bis(trifluoromethylsulfonyl)amide [78]

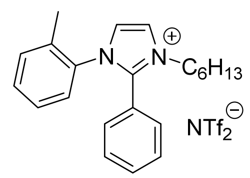

Following general procedure with bromide salt **30** (48.6 g, 122 mmol) and $LiNTf_2$ (55 g, 134 mmol, 70 % aq.) to yield **77** (71.8 g, 120 mmol, 98 %) as a brown liquid.

^{1}H NMR (300 MHz, $CDCl_3$) δ 7.73 (d, *J* = 2.1 Hz, 1H), 7.58 – 7.21 (m, 10H), 4.18 (t, *J* = 7.7 Hz, 2H), 2.04 (s, 3H), 1.83 (quint, *J* = 7.4 Hz, 2H), 1.30 – 1.15 (m, 6H), 0.82 (t, *J* = 6.7 Hz, 3H).

^{13}C NMR (75 MHz, $CDCl_3$) δ 145.1, 134.3, 133.6, 132.8, 131.7, 131.3, 130.2, 129.8, 127.8, 127.7, 123.7, 122.9, 122.1, 120.8, 117.9, 49.7, 30.9, 29.8, 25.9, 22.4, 17.4, 13.9.

Elemental analysis $C_{24}H_{27}F_6N_3O_4S_2$ calc.: C: 48.07 %, H: 4.54 %, N: 7.01 %, S: 10.70 %.
found: C: 48.31 %, H: ´4.92 %, N: 7.14 %, S: 10.86 %.

1-(2-methylphenyl)- 3-octyl-2-phenyl-1*H*-imidazol-3-ium bis(trifluoromethylsulfonyl)amide [79]

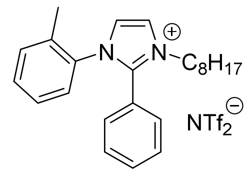

Following general procedure with bromide salt **31** (52.6 g, 123 mmol) and $LiNTf_2$ (55.5 g, 135 mmol, 70 % aq.) to yield **79** (74.6 g, 119 mmol, 97 %) as a brown liquid.

^{1}H NMR (300 MHz, $CDCl_3$) δ 7.72 (d, *J* = 2.1 Hz, 1H), 7.58 – 7.17 (m, 10H), 4.17 (t, *J* = 7.7 Hz, 2H), 2.03 (s, 3H), 1.82 (quint, *J* = 7.4 Hz, 2H), 1.33 – 1.10 (m, 10H), 0.83 (t, *J* = 6.8 Hz, 3H).

^{13}C NMR (75 MHz, $CDCl_3$) δ 145.1, 134.2, 133.6, 132.8, 131.7, 131.3, 130.1, 129.7, 127.9, 127.7, 123.7, 122.8, 122.1, 120.8, 117.9, 49.7, 31.7, 29.8, 28.9, 28.7, 26.2, 22.6, 17.3, 14.1.

Elemental analysis $C_{26}H_{31}F_6N_3O_4S_2$ calc.: C: 49.75 %, H: 4.98 %, N: 6.69 %, S: 10.22 %.
found: C: 50.20 %, H: 5.48 %, N: 6.53 %, S: 10.42 %.

1-(2-methylphenyl)-2-phenyl-3-undecyl-1*H*-imidazol-3-ium bis(trifluoromethylsulfonyl)amide [80]

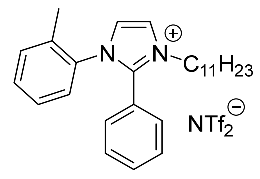

Following general procedure with bromide salt **32** (47.4 g, 104 mmol) and $LiNTf_2$ (47.0 g, 115 mmol, 70 % aq.) to yield **80** (65.1 g, 99.3 mmol, 96 %) as a brown liquid.

^{1}H NMR (300 MHz, $CDCl_3$) δ 7.72 (s, 1H), 7.59 – 7.22 (m, 10H), 4.17 (t, *J* = 7.6 Hz, 2H), 2.04 (s, 3H), 1.92 – 1.78 (m, 2H), 1.32 – 1.12 (m, 16H), 0.86 (t, *J* = 6.7 Hz, 3H).

^{13}C NMR (75 MHz, $CDCl_3$) δ 145.1, 134.3, 133.6, 132.8, 131.7, 131.3, 130.2, 129.8, 127.8, 127.7, 123.7, 122.8, 122.1, 120.8, 117.9, 49.7, 32.0, 29.9, 29.6, 29.5, 29.4, 29.3, 28.8, 26.3, 22.8, 17.3, 14.2.

Elemental analysis $C_{29}H_{37}F_6N_3O_4S_2$ calc.: C: 52.01 %, H: 5.57 %, N: 6.27 %, S: 9.58 %.
found: C: 51.42 %, H: 5.73 %, N: 6.65 %, S: 9.76 %.

3-butyl-1-(4-methoxyphenyl)-2-phenyl-1*H*-imidazol-3-ium bis(trifluoromethylsulfonyl)amide [81]

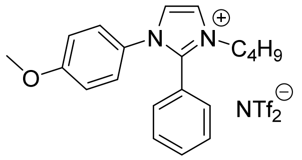

Following general procedure with bromide salt **33** (5.81 g, 15 mmol) and $LiNTf_2$ (6.77 g, 16.5 mmol, 70 % aq.) to yield **81** (8.69 g, 14.8 mmol, 99 %) as a brown liquid.

^{1}H NMR (300 MHz, $CDCl_3$) δ 7.64 – 7.38 (m, 7H), 7.20 (d, *J* = 9.0 Hz, 2H), 6.85 (d, *J* = 9.0 Hz, 2H), 4.12 (t, *J* = 7.6 Hz, 2H), 3.78 (s, 3H), 1.80 (quint, *J* = 7.4 Hz, 2H), 1.29 (sext, *J* = 7.3 Hz, 2H), 0.85 (t, *J* = 7.3 Hz, 3H).

^{13}C NMR (75 MHz, $CDCl_3$) δ 160.9, 144.7, 132.7, 130.6, 129.8, 127.3, 127.3, 123.9, 122.4, 122.1, 121.1, 117.9, 115.2, 55.8, 49.3, 31.9, 19.6, 13.3.

Elemental analysis $C_{22}H_{23}F_6N_3O_5S_2$ calc.: C: 44.97%, H: 3.95%, N: 7.15%, S: 10.91%.
found: C: 45.32%, H: 3.60%, N: 7.37%, S: 10.92%.

3-hexyl-1-(4-methoxyphenyl)-2-phenyl-1*H*-imidazol-3-ium bis(trifluoromethylsulfonyl)amide [82]

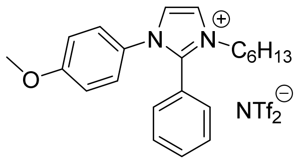

Following general procedure with bromide salt **34** (8.31 g, 20 mmol) and $LiNTf_2$ (9.02 g, 22 mmol, 70 % aq.) to yield **82** (11.2 g, 18.2 mmol, 91 %) as a brown liquid.

^{1}H NMR (300 MHz, $CDCl_3$) δ 7.63 – 7.38 (m, 7H), 7.20 (d, *J* = 9.0 Hz, 2H), 6.85 (d, *J* = 9.0 Hz, 2H), 4.11 (t, *J* = 7.6 Hz, 2H), 3.77 (s, 3H), 1.81 (quint, *J* = 7.5 Hz, 2H), 1.31 – 1.13 (m, 6H), 0.82 (t, *J* = 6.8 Hz, 3H).

^{13}C NMR (75 MHz, $CDCl_3$) δ 160.9, 144.7, 132.7, 130.6, 129.8, 127.3, 127.3, 123.9, 122.4, 122.1, 121.2, 117.9, 115.2, 55.8, 49.5, 30.9, 29.9, 26.0, 22.4, 13.9.

Elemental analysis $C_{24}H_{27}F_6N_3O_5S_2$ calc.: C: 46.83%, H: 4.42%, N: 6.83%, S: 10.42%.
found: C: 46.72%, H: 4.70%, N: 6.35%, S: 10.46%.

1-(4-methoxyphenyl)-3-octyl-2-phenyl-1*H*-imidazol-3-ium bis(trifluoromethylsulfonyl)amide [83]

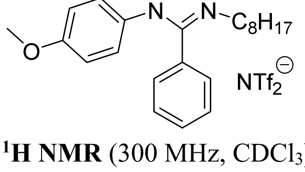

Following general procedure with bromide salt **35** (4.43 g, 10 mmol) and $LiNTf_2$ (4.51 g, 11 mmol, 70 % aq.) to yield **83** (5.8 g, 9.0 mmol, 90 %) as a brown liquid.

^{1}H NMR (300 MHz, $CDCl_3$) δ 7.62 – 7.38 (m, 7H), 7.19 (d, *J* = 9.0 Hz, 2H), 6.85 (d, *J* = 9.0 Hz, 2H), 4.10 (t, *J* = 7.7 Hz, 2H), 3.76 (s, 3H), 1.87 – 1.72 (m, 2H), 1.30 – 1.12 (m, 10H), 0.84 (t, *J* = 6.8 Hz, 3H).

^{13}C NMR (75 MHz, $CDCl_3$) δ 160.9, 144.7, 132.6, 130.6, 129.8, 127.3, 127.3, 123.8, 122.7, 122.2, 121.1, 117.9, 115.1, 55.7, 49.5, 31.7, 29.9, 28.9, 28.7, 26.3, 22.6, 14.1.

Elemental analysis $C_{26}H_{31}F_6N_3O_5S_2$ calc.: C: 48.52%, H: 4.85%, N: 6.53%, S: 9.96%.
found: C: 48.49%, H: 5.12%, N: 6.27%, S: 9.96%.

1-(4-methoxyphenyl)-2-phenyl-3-undecyl-1*H*-imidazol-3-ium bis(trifluoromethylsulfonyl)amide [84]

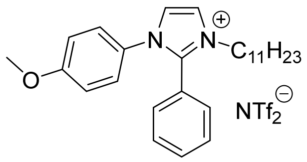

Following general procedure with bromide salt **36** (4.86 g, 10 mmol) and $LiNTf_2$ (4.51 g, 11 mmol, 70 % aq.) to yield **84** (6.49 g, 9.5 mmol, 95 %) as a brown liquid.

^{1}H NMR (300 MHz, $CDCl_3$) δ 7.61 – 7.38 (m, 7H), 7.19 (d, *J* = 9.0 Hz, 2H), 6.85 (d, *J* = 9.0 Hz, 2H), 4.10 (t, *J* = 7.6 Hz, 2H), 3.77 (s, *J* = 1.2 Hz, 3H), 1.80 (quint, *J* = 7.3 Hz, 1H), 1.31 – 1.14 (m, 16H), 0.86 (t, *J* = 6.7 Hz, 3H).

^{13}C NMR (75 MHz, $CDCl_3$) δ 160.9, 144.7, 132.6, 130.6, 129.8, 127.3, 127.3, 123.8, 122.3, 122.1, 121.1, 117.9, 115.2, 55.7, 49.5, 32.0, 29.9, 29.6, 29.5, 29.4, 29.3, 28.8, 26.3, 22.8, 14.2.

Elemental analysis $C_{29}H_{37}F_6N_3O_5S_2$ calc.: C: 50.79 %, H: 5.44%, N: 6.13%, S: 9.35%.
found: C: 50.99%, H: 5.35%, N: 6.11%, S: 9.43%.

3-butyl-1-(2,4-difluorophenyl)-2-phenyl-1*H*-imidazol-3-ium bis(trifluoromethylsulfonyl)amide [85]

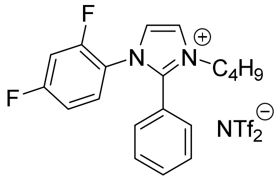

Following general procedure with bromide salt **37** (3.93 g, 10 mmol) and $LiNTf_2$ (4.51 g, 11 mmol, 70 % aq.) to yield **85** (5.3 g, 8.9 mmol, 89 %) as a gray solid.

^{1}H NMR (300 MHz, $CDCl_3$) δ 7.71 – 7.42 (m, 8H), 7.04 – 6.82 (m, 2H), 4.14 (t, *J* = 7.7 Hz, 2H), 1.84 (quint, *J* = 7.5 Hz, 2H), 1.31 (sext, *J* = 7.3 Hz, 2H), 0.87 (t, *J* = 7.3 Hz, 3H).

^{13}C NMR (75 MHz, $CDCl_3$) δ 164.1 (dd, *J* = 256.5, 11.2 Hz), 156.7 (dd, *J* = 256.0, 12.7 Hz), 146.2, 133.1, 130.9, 130.8, 130.2, 129.9, 124.3, 122.8, 122.1, 120.5, 118.9 (dd, *J* = 12.6, 4.2 Hz), 117.9, 113.5 (dd, *J* = 22.9, 3.9 Hz), 105.5 (dd, *J* = 26.9, 22.6 Hz), 49.7, 31.8, 19.6, 13.3.

Melting point: 40°C

Elemental analysis $C_{21}H_{19}F_8N_3O_4S_2$ calc.: C: 42.50%, H: 3.23%, N: 7.08%, S: 10.81%.
found: C: 42.52%, H: 3.19%, N: 7.19%, S: 10.84%.

1-(2,4-difluorophenyl)- 3-hexyl-2-phenyl-1*H*-imidazol-3-ium bis(trifluoromethylsulfonyl)amide [86]

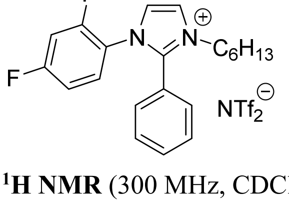

Following general procedure with bromide salt **38** (4.21 g, 10 mmol) and $LiNTf_2$ (4.51 g, 11 mmol, 70 % aq.) to yield **86** (5.6 g, 9.0 mmol, 90 %) as a red liquid.

^{1}H NMR (300 MHz, $CDCl_3$) δ 7.70 – 7.40 (m, 8H), 7.01 – 6.84 (m, 2H), 4.12 (t, *J* = 7.7 Hz, 2H), 1.83 (quint, *J* = 7.5 Hz, 2H), 1.31 – 1.15 (m, 6H), 0.82 (t, *J* = 6.7 Hz, 3H).

^{13}**C NMR** (75 MHz, $CDCl_3$) δ 164.1 (dd, *J* = 256.3, 11.1 Hz), 156.7 (dd, *J* = 256.3, 12.8 Hz), 146.1, 133.1, 130.8, 130.6, 130.2, 129.9, 126.4, 124.3, 122.8, 122.1, 120.4, 118.8 (dd, *J* = 12.6, 4.2 Hz), 117.8, 113.3 (dd, *J* = 22.9, 3.9 Hz), 105.5 (dd, *J* = 26.9, 22.6 Hz), 49.9, 30.9, 29.7, 25.9, 22.3, 13.8.

Elemental analysis $C_{23}H_{23}F_8N_3O_4S_2$ calc.: C: 44.44%, H: 3.73%, N: 6.76%, S: 10.32%.

found: C: 44.46%, H: 3.70%, N: 6.30%, S: 10.28%.

1-(2,4-difluorophenyl)- 3-octyl-2-phenyl-1*H*-imidazol-3-ium bis(trifluoromethylsulfonyl)amide [87]

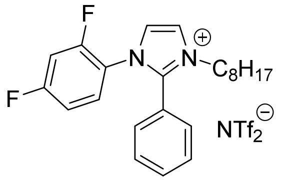

Following general procedure with bromide salt **39** (9.49 g, 10 mmol) and $LiNTf_2$ (4.51 g, 11 mmol, 70 % aq.) to yield **87** (6.0 g, 9.2 mmol, 92 %) as a red liquid.

^{1}**H NMR** (500 MHz, $CDCl_3$) δ 7.68 (d, *J* = 2.2 Hz, 1H), 7.65 – 7.56 (m, 2H), 7.53 – 7.48 (m, 3H), 7.44 – 7.39 (m, 2H), 6.97 – 6.85 (m, 2H), 4.11 (t, *J* = 7.7 Hz, 2H), 1.83 (quint, *J* = 7.5 Hz, 2H), 1.28 – 1.14 (m, 10H), 0.83 (t, *J* = 7.1 Hz, 3H).

^{13}**C NMR** (75 MHz, $CDCl_3$) δ 164.0 (dd, *J* = 256.2, 11.1 Hz), 156.6 (dd, *J* = 256.4, 12.8 Hz), 146.0, 133.1, 130.6, 130.5, 130.1, 129.8, 126.3, 124.2, 122.7, 122.0, 120.3, 118.8 (dd, *J* = 12.5, 4.3 Hz), 117.8, 113.2 (dd, *J* = 23.0, 3.8 Hz), 105.5 (dd, *J* = 27.0, 22.6 Hz), 49.8, 31.6, 29.7, 28.9, 28.7, 26.2, 22.5, 14.0.

Elemental analysis $C_{25}H_{27}F_8N_3O_4S_2$ calc.: C: 46.22%, H: 4.19%, N: 6.47%, S: 9.87%.

found: C: 46.07%, H: 4.20%, N 6.64%, S: 9.75%.

1-(2,4-difluorophenyl)- 2-phenyl-3-undecyl-1*H*-imidazol-3-ium bis(trifluoromethylsulfonyl)amide [88]

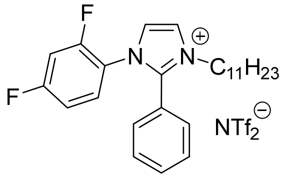

Following general procedure with bromide salt **40** (4.91 g, 10 mmol) and $LiNTf_2$ (4.51 g, 11 mmol, 70 % aq.) to yield **88** (6.1g, 8.8 mmol, 88 %) as a red liquid.

^{1}**H NMR** (300 MHz, $CDCl_3$) δ 7.70 – 7.40 (m, 8H), 7.03 – 6.83 (m, 2H), 4.12 (t, *J* = 7.7 Hz, 2H), 1.84 (quint, *J* = 7.5 Hz, 2H), 1.32 – 1.12 (m, 16H), 0.86 (t, *J* = 6.7 Hz, 3H).

^{13}**C NMR** (75 MHz, $CDCl_3$) δ 164.0 (dd, *J* = 256.4, 11.1 Hz), 156.7 (dd, *J* = 256.1, 12.8 Hz), 146.1, 133.1, 130.8, 130.7, 130.2, 129.9, 126.3, 124.3, 122.8, 122.1, 120.4, 118.8 (dd, *J* = 12.5, 4.2 Hz), 117.8, 113.4 (dd, *J* = 22.9, 3.9 Hz), 105.5 (dd, *J* = 27.0, 22.6 Hz), 49.9, 32.0, 29.8, 29.6, 29.5, 29.4, 29.3, 28.8, 26.3, 22.8, 14.2.

Elemental analysis $C_{28}H_{33}F_8N_3O_4S_2$ calc.: C: 48.62%, H: 4.81%, N: 6.07%, S: 9.27%.

found: C: 48.36%, H: 4.77%, N: 6.28%, S: 9.17%.

3-butyl-2-methyl-1-phenyl-1*H*-imidazol-3-ium bis(trifluoromethylsulfonyl)amide [89]

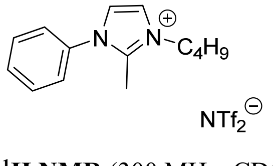

Following general procedure with bromide salt **41** (2.95 g, 10 mmol) and $LiNTf_2$ (4.51 g, 11 mmol, 70 % aq.) to yield **89** (4.7 g, 9.5 mmol, 95 %) as a green liquid.

^{1}**H NMR** (300 MHz, $CDCl_3$) δ 7.62 – 7.56 (m, 3H), 7.48 – 7.41 (m, 2H), 7.39 (d, *J* = 2.2 Hz, 1H), 7.28 (d, *J* = 2.2 Hz, 1H), 4.15 (t, *J* = 7.7 Hz, 2H), 2.54 (s, 3H), 1.94 – 1.81 (m, 2H), 1.44 (sext, *J* = 7.6 Hz, 2H), 0.99 (t, *J* = 7.3 Hz, 3H).

^{13}**C NMR** (75 MHz, $CDCl_3$) δ 144.2, 134.4, 131.3, 130.6, 126.0, 122.5, 121.8, 119.9 (q, *J* = 321.4 Hz), 49.1, 31.3, 19.8, 13.5, 10.4.

Elemental analysis $C_{16}H_{19}F_6N_3O_4S_2$ calc.: C: 38.79%, H: 3.87%, N: 8.48%, S: 12.94%.

found: C: 39.05%, H: 3.88%, N: 8.76%, S: 12.79%.

3-hexyl-2-methyl-1-phenyl-1*H*-imidazol-3-ium bis(trifluoromethylsulfonyl)amide [90]

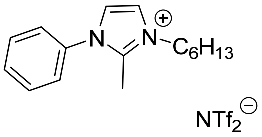

Following general procedure with bromide salt **42** (3.23 g, 10 mmol) and $LiNTf_2$ (4.51 g, 11 mmol, 70 % aq.) to yield **90** (5.0 g, 9.6 mmol, 96 %) as a green liquid.

^{1}**H NMR** (300 MHz, $CDCl_3$) δ 7.62 – 7.54 (m, 3H), 7.48 – 7.41 (m, 2H), 7.38 (d, *J* = 2.2 Hz, 1H), 7.28 (d, *J* = 2.2 Hz, 1H), 4.15 (t, *J* = 7.7 Hz, 2H), 2.54 (s, 3H), 1.89 (quint, *J* = 7.5 Hz, 2H), 1.46 – 1.27 (m, 6H), 0.89 (t, *J* = 7.0 Hz, 3H).

^{13}**C NMR** (75 MHz, $CDCl_3$) δ 144.14, 134.43, 131.26, 130.59, 126.04, 122.49, 121.73, 119.9 (q, *J* = 321.5 Hz), 49.34, 31.15, 29.40, 26.15, 22.46, 13.97, 10.47.

Elemental analysis $C_{18}H_{23}F_6N_3O_4S_2$ calc.: C: 41.30%, H: 4.43%, N: 8.03%, S: 12.25%.

found: C: 41.08%, H: 4.34%, N: 8.28%, S: 12.51%.

2-methyl-3-octyl-1-phenyl-1*H*-imidazol-3-ium bis(trifluoromethylsulfonyl)amide [91]

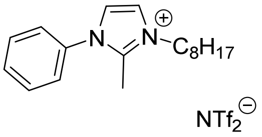

Following general procedure with bromide salt **43** (5.27 g, 15 mmol) and $LiNTf_2$ (6.77 g, 16.5 mmol, 70 % aq.) to yield **91** (7.89g, 14.3 mmol, 95 %) as a green liquid.

^{1}**H NMR** (300 MHz, $CDCl_3$) δ 7.61 – 7.55 (m, 3H), 7.47 – 7.42 (m, 2H), 7.38 (d, *J* = 2.2 Hz, 1H), 7.28 (d, *J* = 2.2 Hz, 1H), 4.14 (t, *J* = 7.7 Hz, 2H), 2.54 (s, 3H), 1.88 (quint, *J* = 7.6 Hz, 2H), 1.42 – 1.23 (m, 10H), 0.87 (t, *J* = 6.8 Hz, 3H).

^{13}**C NMR** (75 MHz, $CDCl_3$) δ 144.1, 134.4, 131.2, 130.6, 126.0, 122.5, 121.7, 119.9 (q, *J* = 321.6 Hz), 49.3, 31.7, 29.4, 29.1, 29.0, 26.5, 22.7, 14.1, 10.4.

Elemental analysis $C_{20}H_{27}F_6N_3O_4S_2$ calc.: C: 43.55%, H: 4.93%, N: 7.62%, S: 11.63%.

found: C: 43.44%, H: 5.04%, N: 7.82%, S: 11.34%.

2-methyl-1-phenyl-3-undecyl-1*H*-imidazol-3-ium bis(trifluoromethylsulfonyl)amide [92]

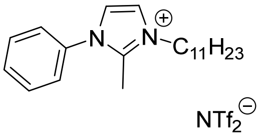

Following general procedure with bromide salt **44** (5.9 g, 10 mmol) and $LiNTf_2$ (6.77 g, 11 mmol, 70 % aq.) to yield **92** (8.47 g, 14.3 mmol, 95 %) as a green liquid.

^{1}**H NMR** (300 MHz, $CDCl_3$) δ 7.63 – 7.55 (m, 3H), 7.49 – 7.40 (m, 2H), 7.38 (d, *J* = 2.2 Hz, 1H), 7.28 (d, *J* = 2.2 Hz, 1H), 4.15 (t, *J* = 7.7 Hz, 2H), 2.54 (s, 3H), 1.89 (quint, *J* = 7.6 Hz, 2H), 1.44 – 1.21 (m, 16H), 0.87 (t, *J* = 6.7 Hz, 3H).

^{13}C NMR (75 MHz, $CDCl_3$) δ 144.1, 134.4, 131.3, 130.6, 126.1, 122.5, 121.7, 119.9 (q, *J* = 321.5 Hz), 49.4, 32.0, 29.6, 29.6, 29.5, 29.4, 29.1, 26.5, 22.8, 14.2, 10.5.

Elemental analysis $C_{23}H_{33}F_6N_3O_4S_2$ calc.: C: 46.53%, H: 5.60%, N: 7.08%, S: 10.80%.
found: C: 46.24%, H: 5.58%, N: 7.34%, S: 10.89%.

3-butyl-2-methyl-1-(2-methylphenyl)-1*H*-imidazol-3-ium bis(trifluoromethylsulfonyl)amide [93]

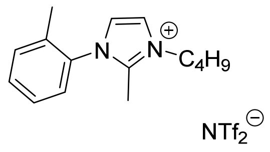

Following general procedure with bromide salt **45** (3.09 g, 10 mmol) and $LiNTf_2$ (4.51 g, 11 mmol, 70 % aq.) to yield **93** (4.9 g, 9.6 mmol, 96 %) as a yellow liquid.

^{1}H NMR (300 MHz, $CDCl_3$) δ 7.55 – 7.31 (m, 5H), 7.20 (d, *J* = 2.1 Hz, 1H), 4.19 (t, *J* = 7.6 Hz, 2H), 2.42 (s, 3H), 2.07 (s, 3H), 1.88 (quint, *J* = 7.5 Hz, 2H), 1.42 (sext, *J* = 7.4 Hz, 2H), 0.99 (t, *J* = 7.3 Hz, 3H).

^{13}C NMR (75 MHz, $CDCl_3$) δ 144.5, 134.6, 133.3, 132.1, 131.8, 128.2, 127.3, 122.5, 122.1, 119.9 (q, *J* = 321.4 Hz), 49.2, 31.4, 19.7, 17.1, 13.5, 10.1.

Elemental analysis $C_{17}H_{21}F_6N_3O_4S_2$ calc.: C: 40.08%, H: 4.15%, N: 8.25%, S: 12.59%.
found: C: 39.72%, H: 4.12%, N: 8.12%, S: 12.62%.

3-hexyl-2-methyl-1-(2-methylphenyl)-1*H*-imidazol-3-ium bis(trifluoromethylsulfonyl)amide [94]

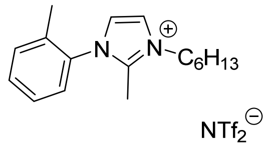

Following general procedure with bromide salt **46** (3.37 g, 10 mmol) and $LiNTf_2$ (4.51 g, 11 mmol, 70 % aq.) to yield **94** (4.91 g, 9.1 mmol, 91 %) as a yellow liquid.

^{1}H NMR (300 MHz, $CDCl_3$) δ 7.54 – 7.31 (m, 5H), 7.20 (d, *J* = 2.2 Hz, 1H), 4.18 (t, *J* = 7.6 Hz, 2H), 2.42 (s, 3H), 2.07 (s, 3H), 1.89 (quint, *J* = 7.3 Hz, 2H), 1.44 – 1.27 (m, 6H), 0.88 (t, *J* = 6.9 Hz, 3H).

^{13}C NMR (75 MHz, $CDCl_3$) δ 144.4, 134.6, 133.3, 132.1, 131.7, 128.2, 127.3, 122.5, 122.0, 119.9 (q, *J* = 321.5 Hz), 49.3, 31.1, 29.4, 26.0, 22.4, 17.0, 13.9, 10.0.

Elemental analysis $C_{19}H_{25}F_6N_3O_4S_2$ calc.: C: 42.45%, H: 4.69%, N: 7.82%, S: 11.93%.
found: C: 42.38%, H: 4.87%, N: 7.80%, S: 11.91%.

2-methyl-1-(2-methylphenyl)-3-octyl-1*H*-imidazol-3-ium bis(trifluoromethylsulfonyl)amide [95]

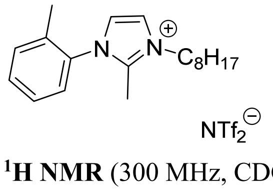

Following general procedure with bromide salt **47** (3.65 g, 10 mmol) and $LiNTf_2$ (4.51 g, 11 mmol, 70 % aq.) to yield **95** (5.28 g, 9.3 mmol, 93 %) as a yellow liquid.

^{1}H NMR (300 MHz, $CDCl_3$) δ 7.55 – 7.33 (m, 5H), 7.21 (d, *J* = 2.1 Hz, 1H), 4.26 – 4.11 (m, 2H), 2.43 (s, 3H), 2.08 (s, 3H), 1.97 – 1.82 (m, 2H), 1.44 – 1.19 (m, 10H), 0.87 (t, *J* = 6.5 Hz, 3H), 0.06 (s, 1H).

^{13}C NMR (75 MHz, $CDCl_3$) δ 144.5, 134.6, 133.3, 132.1, 131.8, 128.2, 127.3, 122.5, 122.1, 119.9 (q, *J* = 321.4 Hz), 49.4, 31.7, 29.5, 29.1, 29.0, 26.4, 22.7, 17.1, 14.1, 10.1.

Elemental analysis $C_{21}H_{29}F_6N_3O_4S_2$ calc.: C: 44.60%, H: 5.17%, N: 7.42%, S: 11.34%.

found: C: 44.37%, H: 5.09%, N: 7.55%, S: 11.19%.

2-methyl-1-(2-methylphenyl)-3-undecyl-1*H*-imidazol-3-ium bis(trifluoromethylsulfonyl)amide [96]

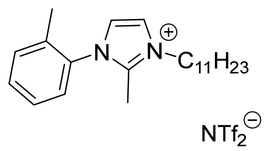

Following general procedure with bromide salt **48** (4.07 g, 10 mmol) and $LiNTf_2$ (4.51 g, 11 mmol, 70 % aq.) to yield **96** (5.8 g, 9.5 mmol, 95 %) as a yellow liquid.

^{1}H NMR (300 MHz, $CDCl_3$) δ 7.55 – 7.33 (m, 5H), 7.21 (d, *J* = 2.1 Hz, 1H), 4.19 (t, *J* = 7.5 Hz, 2H), 2.43 (s, 3H), 2.08 (s, 3H), 1.90 (quint, *J* = 7.5 Hz, 2H), 1.41 – 1.19 (m, 16H), 0.87 (t, *J* = 6.6 Hz, 3H).

^{13}C NMR (75 MHz, $CDCl_3$) δ 144.5, 134.6, 133.3, 132.1, 131.8, 128.2, 127.3, 122.5, 122.1, 119.9 (q, *J* = 321.6 Hz), 49.4, 32.0, 29.6, 29.6, 29.5, 29.4, 29.1, 26.5, 22.8, 17.1, 14.2, 10.1.

Elemental analysis $C_{24}H_{35}F_6N_3O_4S_2$ calc.: C: 47.44%, H: 5.81%, N: 6.92%, S: 10.55%.

found: C: 47.28%, H: 6.12%, N: 6.89%, S: 10.52%.

3-butyl-1-(4-methoxyphenyl)-2-methyl-1*H*-imidazol-3-ium bis(trifluoromethylsulfonyl)amide [97]

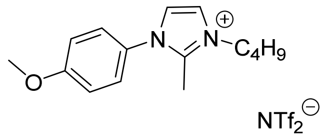

Following general procedure with bromide salt **49** (2.6 g, 8 mmol) and $LiNTf_2$ (3.61 g, 8.8 mmol, 70 % aq.) to yield **97** (4.1 g, 7.8 mmol, 98 %) as a brown liquid.

^{1}H NMR (300 MHz, $CDCl_3$) δ 7.40 – 7.30 (m, 3H), 7.22 (d, *J* = 2.2 Hz, 1H), 7.04 (d, *J* = 9.0 Hz, 2H), 4.12 (t, *J* = 7.6 Hz, 2H), 3.86 (s, 3H), 2.51 (s, 3H), 1.86 (quint, *J* = 7.6 Hz, 2H), 1.42 (sext, *J* = 7.5 Hz, 2H), 0.98 (t, *J* = 7.3 Hz, 3H).

^{13}C NMR (75 MHz, $CDCl_3$) δ 161.4, 144.3, 127.3, 126.9, 122.8, 121.5, 119.9 (q, *J* = 321.5 Hz), 115.5, 55.9, 49.0, 31.3, 19.7, 13.4, 10.3.

3-hexyl-1-(4-methoxyphenyl)-2-methyl-1*H*-imidazol-3-ium bis(trifluoromethylsulfonyl)amide [98]

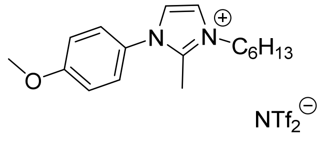

Following general procedure with bromide salt **50** (1.0 g, 2.83 mmol) and $LiNTf_2$ (1.27 g, 3.11 mmol, 70 % aq.) to yield **98** (1.37 g, 2.5 mmol, 88 %) as a brown liquid.

^{1}H NMR (300 MHz, $CDCl_3$) δ 7.43 – 7.31 (m, 3H), 7.23 (d, *J* = 2.1 Hz, 1H), 7.04 (d, *J* = 8.9 Hz, 2H), 4.13 (t, *J* = 7.7 Hz, 2H), 3.86 (s, 3H), 2.51 (s, 3H), 1.87 (quint, *J* = 7.5 Hz, 2H), 1.46 – 1.26 (m, 6H), 0.89 (t, *J* = 6.9 Hz, 3H).

^{13}C NMR (75 MHz, $CDCl_3$) δ 161.4, 144.3, 127.3, 126.9, 122.8, 121.4, 119.9 (q, J = 321.6 Hz), 115.5, 55.9, 49.3, 31.1, 29.4, 26.1, 22.4, 13.9, 10.3.

**Elemental analysis** $C_{19}H_{25}F_6N_3O_5S_2$	calc.:	C: 41.23%, H: 4.55%, N: 7.59%, S: 11.58%.
	found:	C: 41.08%, H: 4.90%, N: 7.56%, S: 11.54%.

1-(4-methoxyphenyl)-2-methyl-3-octyl-1*H*-imidazol-3-ium bis(trifluoromethylsulfonyl)amide [99]

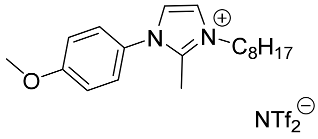

Following general procedure with bromide salt **51** (3.1 g, 8 mmol) and $LiNTf_2$ (3.61 g, 8.8 mmol, 70 % aq.) to yield **99** (4.56 g, 7.8 mmol, 98 %) as a brown liquid.

^{1}H NMR (300 MHz, $CDCl_3$) δ 7.38 – 7.31 (m, 3H), 7.23 (d, J = 2.1 Hz, 1H), 7.04 (d, J = 9.0 Hz, 2H), 4.11 (t, J = 7.6 Hz, 2H), 3.86 (s, 3H), 2.51 (s, 3H), 1.87 (quint, J = 7.5 Hz, 2H), 1.42 – 1.23 (m, 10H), 0.86 (t, J = 6.7 Hz, 3H).

^{13}C NMR (75 MHz, $CDCl_3$) δ 161.4, 144.3, 127.3, 126.9, 122.8, 121.4, 119.9 (q, J = 321.4 Hz), 115.5, 55.9, 49.3, 31.7, 29.4, 29.1, 29.0, 26.5, 22.6, 14.1, 10.3.

1-(4-methoxyphenyl)-2-methyl-3-undecyl-1*H*-imidazol-3-ium bis(trifluoromethylsulfonyl)amide [100]

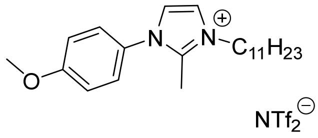

Following general procedure with bromide salt **52** (3.39 g, 8 mmol) and $LiNTf_2$ (3.61 g, 8.8 mmol, 70 % aq.) to yield **100** (4.58 g, 7.3 mmol, 92 %) as a brown liquid.

^{1}H NMR (300 MHz, $CDCl_3$) δ 7.43 – 7.30 (m, 3H), 7.23 (d, J = 2.1 Hz, 1H), 7.04 (d, J = 9.0 Hz, 2H), 4.12 (t, J = 7.6 Hz, 2H), 3.86 (s, 3H), 2.51 (s, 3H), 1.87 (quint, J = 7.5 Hz, 2H), 1.40 – 1.18 (m, 16H), 0.87 (t, J = 6.7 Hz, 3H).

^{13}C NMR (75 MHz, $CDCl_3$) δ 161.4, 144.3, 127.4, 126.9, 122.8, 121.4, 119.9 (q, J = 321.6 Hz), 115.6, 55.9, 49.3, 32.0, 29.6, 29.6, 29.5, 29.4, 29.4, 29.1, 26.5, 22.8, 14.2, 10.4.

3-butyl-1-(2,4-difluorophenyl)-2-methyl-1*H*-imidazol-3-ium bis(trifluoromethylsulfonyl)amide [101]

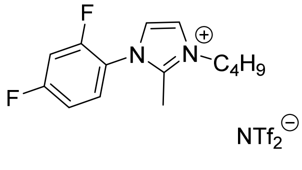

Following general procedure with bromide salt **53** (3.31 g, 10 mmol) and $LiNTf_2$ (4.51 g, 11 mmol, 70 % aq.) to yield **101** (5.0 g, 9.4 mmol, 94 %) as a red liquid.

^{1}H NMR (300 MHz, $CDCl_3$) δ 7.64 (td, *J* = 8.6, 5.4 Hz, 1H), 7.44 (d, *J* = 2.2 Hz, 1H), 7.29 (d, *J* = 2.2 Hz, 1H), 7.15 – 7.04 (m, 2H), 4.14 (t, *J* = 7.7 Hz, 2H), 2.48 (s, 3H), 1.85 (quint, *J* = 7.5 Hz, 2H), 1.40 (sext, *J* = 7.4 Hz, 2H), 0.95 (t, *J* = 7.3 Hz, 3H).

^{13}C NMR (75 MHz, $CDCl_3$) δ 164.2 (dd, *J* = 256.3, 11.1 Hz), 156.7 (dd, *J* = 256.5, 12.9 Hz), 145.3, 130.2 (d, *J* = 10.5 Hz), 123.0, 122.1, 119.8 (q, *J* = 321.5 Hz),118.3 (dd, *J* = 12.6, 4.2 Hz), 113.6 (dd, *J* = 23.0, 3.9 Hz), 105.9 (dd, *J* = 27.0, 22.6 Hz), 49.3, 31.2, 19.5, 13.3.

1-(2,4-difluorophenyl)-3-hexyl-2-methyl-1*H*-imidazol-3-ium bis(trifluoromethylsulfonyl)amide [102]

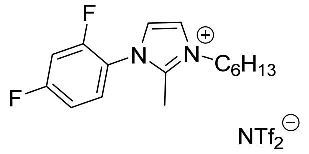

Following general procedure with bromide salt **54** (3.59 g, 10 mmol) and $LiNTf_2$ (4.51 g, 11 mmol, 70 % aq.) to yield **102** (5.4 g, 9.6 mmol, 96 %) as a red liquid.

^{1}H NMR (300 MHz, $CDCl_3$) δ 7.72 (td, *J* = 8.7, 5.5 Hz, 1H), 7.43 (d, *J* = 2.2 Hz, 1H), 7.29 (d, *J* = 2.2 Hz, 1H), 7.22 – 7.04 (m, 2H), 4.17 (t, *J* = 7.7 Hz, 2H), 2.51 (s, *J* = 0.7 Hz, 3H), 1.90 (quint, *J* = 7.4 Hz, 2H), 1.47 – 1.23 (m, 6H), 0.89 (t, *J* = 7.0 Hz, 3H).

^{13}C NMR (75 MHz, $CDCl_3$) δ 164.4 (dd, *J* = 256.8, 11.1 Hz), 156.8 (dd, *J* = 256.2, 12.8 Hz), 145.4, 130.5 (d, *J* = 10.5 Hz), 123.1, 122.2, 119.9 (q, *J* = 321.4 Hz), 118.4 (d, *J* = 12.6 Hz), 113.8 (dd, *J* = 22.9, 3.9 Hz), 105.9 (dd, *J* = 27.0, 22.6 Hz), 49.7, 31.1, 29.4, 26.9, 22.5, 13.9, 10.3 (d, *J* = 1.8 Hz).

1-(2,4-difluorophenyl)-2-methyl-3-octyl-1*H*-imidazol-3-ium bis(trifluoromethylsulfonyl)amide [103]

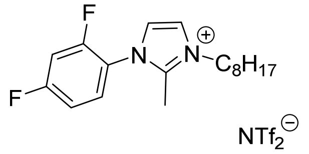

Following general procedure with bromide salt **55** (3.87 g, 10 mmol) and $LiNTf_2$ (4.51 g, 11 mmol, 70 % aq.) to yield **103** (5.44 g, 9.3 mmol, 93 %) as a red liquid.

^{1}H NMR (300 MHz, $CDCl_3$) δ 7.74 – 7.64 (m, 1H), 7.43 (d, *J* = 2.2 Hz, 1H), 7.30 (d, *J* = 2.1 Hz, 1H), 7.18 – 7.06 (m, 2H), 4.16 (t, *J* = 7.7 Hz, 2H), 2.51 (s, 3H), 1.89 (quint, *J* = 7.3 Hz, 2H), 1.42 – 1.18 (m, 10H), 0.86 (t, *J* = 6.7 Hz, 3H).

^{13}C NMR (75 MHz, $CDCl_3$) δ 164.3 (dd, *J* = 256.7, 11.1 Hz), 156.7 (dd, *J* = 256.3, 12.8 Hz), 145.4, 130.4 (d, *J* = 10.5 Hz), 123.1, 122.1, 119.9 (q, *J* = 321.5 Hz), 118.4 (dd, *J* = 12.5, 4.2 Hz), 113.8 (dd, *J* = 22.9, 3.8 Hz), 105.9 (dd, *J* = 26.9, 22.6 Hz), 49.6, 31.7, 29.4, 29.1, 28.0, 26.4, 22.6, 14.1, 10.2 (d, *J* = 1.7 Hz).

1-(2,4-difluorophenyl)-2-methyl-3-undecyl-1*H*-imidazol-3-ium bis(trifluormethansulfonyl)-imid [104]

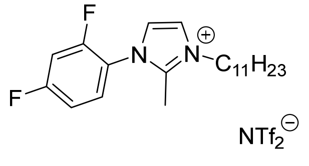

Following general procedure with bromide salt **56** (4.29 g, 10 mmol) and $LiNTf_2$ (4.51 g, 11 mmol, 70 % aq.) to yield **104** (6.0 g, 9.5 mmol, 95 %) as a red liquid.

^{1}H NMR (300 MHz, $CDCl_3$) δ 7.77 – 7.63 (m, 1H), 7.43 (d, *J* = 2.2 Hz, 1H), 7.30 (d, *J* = 2.2 Hz, 1H), 7.17 – 7.05 (m, 2H), 4.16 (t, *J* = 7.6 Hz, 2H), 2.50 (s, 3H), 1.87 (quint, *J* = 7.3 Hz, 2H), 1.30 (m, 16H), 0.86 (t, *J* = 6.6 Hz, 3H).

^{13}C NMR (75 MHz, $CDCl_3$) δ 164.3 (dd, *J* = 256.7, 11.2 Hz), 156.7 (dd, *J* = 256.4, 12.8 Hz), 145.4, 130.4 (d, *J* = 10.5 Hz), 123.1, 122.1, 119.9 (q, *J* = 321.3 Hz), 118.4 (dd, *J* = 12.6, 4.3 Hz), 113.8 (dd, *J* = 23.0, 3.9 Hz), 105.9 (dd, *J* = 27.0, 22.6 Hz), 49.6, 32.0, 29.6, 29.6, 29.4, 29.4, 29.3, 29.0, 26.4, 22.8, 14.2, 10.2 (d, *J* = 1.8 Hz).

3-butyl-1-phenyl-2-propyl-1*H*-imidazol-3-ium bis(trifluoromethylsulfonyl)amide [105]

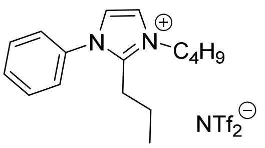

Following general procedure with bromide salt **57** (3.23 g, 10 mmol) and $LiNTf_2$ (4.51 g, 11 mmol, 70 % aq.) to yield **105** (4.71 g, 9.0 mmol, 90 %) as a yellow liquid.

^{1}H NMR (300 MHz, $CDCl_3$) δ 7.69 – 7.55 (m, 3H), 7.50 – 7.40 (m, 3H), 7.28 (d, *J* = 2.1 Hz, 1H), 4.17 (t, *J* = 7.8 Hz, 2H), 2.87 (t, *J* = 8.0 Hz, 2H), 2.01 – 1.85 (m, 2H), 1.59 – 1.41 (m, 4H), 1.01 (t, *J* = 7.3 Hz, 3H), 0.86 (t, *J* = 7.4 Hz, 3H).

^{13}C NMR (75 MHz, $CDCl_3$) δ 147.2, 134.5, 131.5, 130.7, 126.2, 123.1, 121.8, 120.0 (q, *J* = 321.5 Hz), 48.9, 31.9, 25.5, 21.0, 19.8, 13.8, 13.5.

**Elemental analysis** $C_{18}H_{23}F_6N_3O_4S_2$	calc.:	C: 41.30%, H: 4.43%, N: 8.03%, S: 12.25%.
	found:	C: 41.09%, H: 4.65%, N: 8.02%, S: 12.13%.

3-hexyl-1-phenyl-2-propyl-1*H*-imidazol-3-ium bis(trifluoromethylsulfonyl)amide [106]

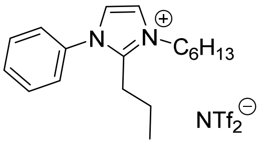

Following general procedure with bromide salt **58** (5.27 g, 15 mmol) and $LiNTf_2$ (6.77 g, 16.5 mmol, 70 % aq.) to yield **106** (7.9 g, 14.4 mmol, 96 %) as a yellow liquid.

^{1}H NMR (300 MHz, $CDCl_3$) δ 7.66 – 7.55 (m, 3H), 7.49 – 7.41 (m, 3H), 7.28 (d, *J* = 2.2 Hz, 1H), 4.15 (t, *J* = 7.8 Hz, 2H), 2.86 (t, *J* = 8.0 Hz, 2H), 1.92 (quint, *J* = 7.5 Hz, 2H), 1.58 – 1.30 (m, 8H), 0.94 – 0.81 (m, 6H).

^{13}C NMR (75 MHz, $CDCl_3$) δ 147.1, 134.5, 131.4, 130.6, 126.2, 123.0, 121.7, 120.0 (q, *J* = 321.6 Hz), 49.0, 31.1, 29.8, 26.1, 25.4, 22.4, 20.9, 13.9, 13.7.

**Elemental analysis** $C_{20}H_{27}F_6N_3O_4S_2$	calc.:	C: 43.55%, H: 4.93%, N: 7.62%, S: 11.63%.
	found:	C: 43.44%, H: 4.90%, N: 7.80%, S: 11.52%.

3-octyl-1-phenyl-2-propyl-1*H*-imidazol-3-ium bis(trifluoromethylsulfonyl)amide [107]

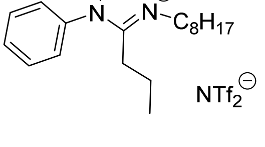

Following general procedure with bromide salt **59** (3.79 g, 10 mmol) and $LiNTf_2$ (4.51 g, 11 mmol, 70 % aq.) to yield **107** (5.5 g, 9.5 mmol, 95 %) as a yellow liquid.

^{1}H NMR (300 MHz, $CDCl_3$) δ 7.65 – 7.56 (m, 3H), 7.49 – 7.41 (m, 3H), 7.28 (d, *J* = 2.1 Hz, 1H), 4.16 (t, *J* = 7.8 Hz, 2H), 2.87 (t, *J* = 8.0 Hz, 2H), 1.92 (t, *J* = 7.4 Hz, 2H), 1.58 – 1.22 (m, 12H), 0.92 – 0.81 (m, 6H).

^{13}C NMR (75 MHz, $CDCl_3$) δ 147.2, 134.5, 131.4, 130.7, 126.2, 123.1, 121.7, 120.0 (q, *J* = 321.5 Hz), 49.1, 31.8, 29.9, 29.1, 29.0, 26.6, 25.5, 22.7, 21.0, 14.1, 13.8.

Elemental analysis $C_{22}H_{31}F_6N_3O_4S_2$ calc.: C: 45.59%, H: 5.39%, N: 7.25%, S: 11.06%.

found: C: 45.36%, H: 5.38%, N: 7.50%, S: 10.86%.

1-phenyl-2-propyl-3-undecyl-1*H*-imidazol-3-ium bis(trifluoromethylsulfonyl)amide [108]

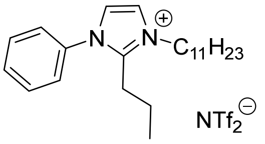

Following general procedure with bromide salt **60** (4.21 g, 10 mmol) and $LiNTf_2$ (4.51 g, 11 mmol, 70 % aq.) to yield **108** (5.8 g, 9.3 mmol, 93 %) as a yellow liquid.

^{1}H NMR (300 MHz, $CDCl_3$) δ 7.65 – 7.55 (m, 3H), 7.49 – 7.40 (m, 3H), 7.28 (d, *J* = 2.1 Hz, 1H), 4.15 (t, *J* = 7.8 Hz, 2H), 2.87 (t, *J* = 8.0 Hz, 2H), 1.91 (quint, *J* = 7.5 Hz, 2H), 1.58 – 1.22 (m, 18H), 0.91 – 0.82 (m, 6H).

^{13}C NMR (75 MHz, $CDCl_3$) δ 147.2, 134.5, 131.4, 130.6, 126.2, 123.1, 121.7, 120.0 (q, *J* = 321.6 Hz), 49.1, 31.9, 29.9, 29.6, 29.6, 29.4, 29.4, 29.1, 26.5, 25.5, 22.8, 21.0, 14.2, 13.8.

Elemental analysis $C_{25}H_{37}F_6N_3O_4S_2$ calc.: C: 48.30%, H: 6.00%, N: 6.76%, S: 10.32%.

found: C: 48.08%, H: 5.91%, N: 7.13%, S: 10.24%.

3-butyl-1-(2-methylphenyl)-2-propyl-1*H*-imidazol-3-ium bis(trifluoromethylsulfonyl)amide [109]

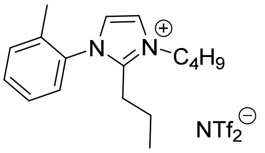

Following general procedure with bromide salt **61** (3.37 g, 10 mmol) and $LiNTf_2$ (4.51 g, 11 mmol, 70 % aq.) to yield **109** (5.3 g, 9.9 mmol, 99 %) as a brown liquid.

^{1}H NMR (300 MHz, $CDCl_3$) δ 7.52 – 7.46 (m, 2H), 7.43 – 7.28 (m, 3H), 7.18 (d, *J* = 2.2 Hz, 1H), 4.16 (t, *J* = 7.7 Hz, 2H), 2.72 (ddt, *J* = 72.3, 15.5, 7.6 Hz, 2H), 2.04 (s, 3H), 1.95 – 1.81 (m, 2H), 1.52 – 1.34 (m, 4H), 0.96 (t, *J* = 7.3 Hz, 3H), 0.82 (t, *J* = 7.4 Hz, 3H).

^{13}C NMR (75 MHz, $CDCl_3$) δ 147.3, 134.6, 133.2, 132.2, 131.8, 128.1, 127.2, 122.9, 122.1, 119.9 (q, *J* = 321.4 Hz) 48.9, 31.8, 25.4, 20.7, 19.7, 17.1, 13.7, 13.5.

3-hexyl-1-(2-methylphenyl)-2-propyl-1H-imidazol-3-ium bis(trifluoromethylsulfonyl)amide [110]

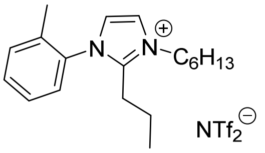

Following general procedure with bromide salt **62** (3.65 g, 10 mmol) and $LiNTf_2$ (4.51 g, 11 mmol, 70 % aq.) to yield **110** (5.37 g, 9.5 mmol, 95 %) as a brown liquid.

^{1}H NMR (300 MHz, $CDCl_3$) δ 7.59 – 7.49 (m, 2H), 7.45 – 7.31 (m, 3H), 7.21 (d, *J* = 2.1 Hz, 1H), 4.19 (t, *J* = 7.7 Hz, 2H), 2.75 (ddt, *J* = 69.6, 15.7, 8.0 Hz, 2H), 2.08 (s, 3H), 1.92 (quint, *J* = 7.5 Hz, 2H), 1.55 – 1.27 (m, 8H), 0.93 – 0.82 (m, 6H).

^{13}C NMR (75 MHz, $CDCl_3$) δ 147.3, 134.6, 133.2, 132.2, 131.8, 128.1, 127.2, 122.9, 122.1, 119.9 (q, *J* = 321.4 Hz), 49.1, 31.1, 29.9, 26.1, 25.4, 22.4, 20.8, 17.1, 13.9, 13.8.

1-(2-methylphenyl)-3-octyl-2-propyl-1*H*-imidazol-3-ium bis(trifluoromethylsulfonyl)amide [111]

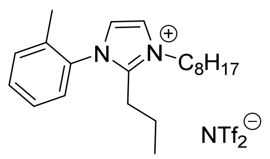

Following general procedure with bromide salt **63** (3.93 g, 10 mmol) and $LiNTf_2$ (4.51 g, 11 mmol, 70 % aq.) to yield **111** (5.71 g, 9.6 mmol, 96 %) as a brown liquid.

^{1}H NMR (300 MHz, $CDCl_3$) δ 7.57 – 7.49 (m, 2H), 7.47 – 7.29 (m, 3H), 7.22 (d, *J* = 2.1 Hz, 1H), 4.19 (t, *J* = 7.0 Hz, 2H), 2.77 (ddt, *J* = 69.0, 15.4, 7.6 Hz, 2H), 2.08 (s, 3H), 1.92 (quint, *J* = 6.9 Hz, 2H), 1.54 – 1.21 (m, 12H), 0.87 (t, *J* = 6.1 Hz, 6H).

^{13}C NMR (75 MHz, $CDCl_3$) δ 147.3, 134.6, 133.2, 132.2, 131.8, 128.1, 127.2, 122.9, 122.1, 119.9 (q, *J* = 321.4 Hz).49.1, 31.7, 29.9, 29.0, 29.0, 26.4, 25.4, 22.6, 20.7, 17.1, 14.1, 13.7.

1-(2-methylphenyl) -2-propyl-3-undecyl-1*H*-imidazol-3-ium bis(trifluoromethylsulfonyl)amide [112]

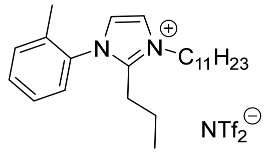

Following general procedure with bromide salt **64** (4.35 g, 10 mmol) and $LiNTf_2$ (4.51 g, 11 mmol, 70 % aq.) to yield **112** (5.87 g, 9.2 mmol, 92 %) as a brown liquid.

^{1}H NMR (300 MHz, $CDCl_3$) δ 7.57 – 7.50 (m, 2H), 7.46 – 7.33 (m, 3H), 7.22 (d, *J* = 2.1 Hz, 1H), 4.20 (t, *J* = 7.7 Hz, 2H), 2.75 (ddt, *J* = 67.9, 15.7, 7.7 Hz, 2H), 2.09 (s, 3H), 1.91 (quint, *J* = 7.4 Hz, 2H), 1.55 – 1.23 (m, 18H), 0.87 (t, *J* = 7.2 Hz, 6H).

^{13}C NMR (75 MHz, $CDCl_3$) δ 147.3, 134.6, 133.3, 132.2, 131.8, 128.1, 127.3, 122.9, 122.2, 119.9 (q, *J* = 321.5 Hz) 49.2, 32.0, 30.0, 29.6, 29.6, 29.4, 29.4, 29.1, 26.5, 25.5, 22.8, 20.8, 17.1, 14.2, 13.8.

3-butyl-1-(4-methyoxyphenyl)-2-propyl-1*H*-imidazol-3-ium bis(trifluoromethylsulfonyl)amide [113]

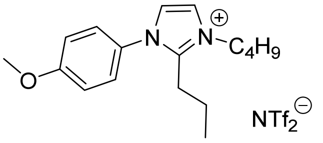

Following general procedure with bromide salt **65** (2.83 g, 8 mmol) and $LiNTf_2$ (3.61 g, 8.8 mmol, 70 % aq.) to yield **113** (4.25 g, 7.7 mmol, 96 %) as a brown liquid.

^{1}H NMR (300 MHz, $CDCl_3$) δ 7.38 (d, *J* = 2.1 Hz, 1H), 7.33 (d, *J* = 9.0 Hz, 2H), 7.21 (d, *J* = 2.1 Hz, 1H), 7.02 (d, *J* = 9.0 Hz, 2H), 4.11 (t, *J* = 7.8 Hz, 2H), 3.84 (s, 3H), 2.81 (t, *J* = 8.0 Hz, 2H), 1.87 (quint, *J* = 7.7 Hz, 2H), 1.56 – 1.35 (m, 4H), 0.96 (t, *J* = 7.3 Hz, 3H), 0.83 (t, *J* = 7.4 Hz, 3H).

^{13}C NMR (75 MHz, $CDCl_3$) δ 161.4, 147.3, 127.4, 126.8, 123.2, 121.3, 119.8 (q, *J* = 321.6 Hz), 115.5, 55.7, 48.6, 31.7, 25.3, 20.8, 19.62, 13.6, 13.3.

3-hexyl-1-(4-methyoxyphenyl)-2-propyl-1*H*-imidazol-3-ium bis(trifluoromethylsulfonyl)amide [114]

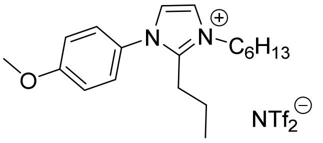

Following general procedure with bromide salt **66** (3.05 g, 8 mmol) and $LiNTf_2$ (3.61 g, 8.8 mmol, 70 % aq.) to yield **114** (4.4 g, 7.53 mmol, 94 %) as a brown liquid.

^{1}H NMR (300 MHz, $CDCl_3$) δ 7.38 (d, *J* = 2.1 Hz, 1H), 7.32 (d, *J* = 9.0 Hz, 2H), 7.21 (d, *J* = 2.1 Hz, 1H), 7.02 (d, *J* = 9.0 Hz, 2H), 4.10 (t, *J* = 7.8 Hz, 2H), 3.83 (s, 3H), 2.81 (t, *J* = 7.9 Hz, 2H), 1.87 (quint, *J* = 7.7 Hz, 2H), 1.55 – 1.25 (m, 8H), 0.89 – 0.78 (m, 6H).

^{13}C NMR (75 MHz, $CDCl_3$) δ 161.4, 147.2, 127.4, 126.8, 123.2, 121.3, 119.8 (q, *J* = 321.6 Hz), 115.4, 55.7, 48.9, 31.6, 29.7, 28.9, 28.9, 26.3, 25.3, 22.5, 20.8, 14.0, 13.6.

1-(4-methyoxyphenyl)-3-octyl-2-propyl-1*H*-imidazol-3-ium bis(trifluoromethylsulfonyl)amide [115]

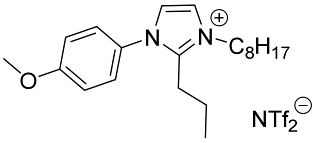

Following general procedure with bromide salt **67** (3.28 g, 8 mmol) and $LiNTf_2$ (3.61 g, 8.8 mmol, 70 % aq.) to yield **115** (4.6 g, 7.61 mmol, 95 %) as a brown liquid.

^{1}H NMR (300 MHz, $CDCl_3$) δ 7.37 (d, *J* = 2.1 Hz, 1H), 7.33 (d, *J* = 8.9 Hz, 2H), 7.21 (d, *J* = 2.1 Hz, 1H), 7.02 (d, *J* = 8.9 Hz, 2H), 4.10 (t, *J* = 7.8 Hz, 2H), 3.84 (s, 3H), 2.81 (t, *J* = 8.0 Hz, 2H), 1.88 (quint, *J* = 7.5 Hz, 2H), 1.55 – 1.21 (m, 12H), 0.83 (t, *J* = 7.3 Hz, 6H).

^{13}C NMR (75 MHz, $CDCl_3$) δ 161.4, 147.2, 127.4, 126.8, 123.2, 121.3, 119.8 (q, *J* = 321.6 Hz), 115.4, 55.7, 48.8, 31.0, 29.7, 26.0, 25.2, 22.3, 20.8, 13.8, 13.6.

1-(4-methyoxyphenyl)-2-propyl-3-undecyl-1*H*-imidazol-3-ium bis(trifluoromethylsulfonyl)amide [116]

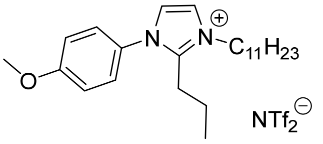

Following general procedure with bromide salt **68** (3.61 g, 8 mmol) and $LiNTf_2$ (3.61 g, 8.8 mmol, 70 % aq.) to yield **116** (5.0 g, 7.64 mmol, 96 %) as a brown liquid.

^{1}H NMR (300 MHz, $CDCl_3$) δ 7.39 (d, *J* = 2.1 Hz, 1H), 7.35 (d, *J* = 8.9 Hz, 2H), 7.23 (d, *J* = 2.1 Hz, 1H), 7.05 (d, *J* = 8.9 Hz, 2H), 4.13 (t, *J* = 7.8 Hz, 2H), 3.87 (s, 3H), 2.83 (t, *J* = 8.0 Hz, 2H), 1.90 (quint, *J* = 7.5 Hz, 2H), 1.57 – 1.19 (m, 18H), 0.86 (t, *J* = 7.2 Hz, 6H).

^{13}C NMR (75 MHz, $CDCl_3$) δ 161.5, 147.4, 127.5, 126.9, 123.4, 121.4, 119.9 (q, *J* = 321.6 Hz), 115.6, 55.9, 49.0, 32.0, 29.9, 29.6, 29.6, 29.4, 29.4, 29.3, 26.5, 25.4, 22.8, 21.0, 14.1, 13.8.

3-butyl-1-(2,4-difluorophenyl)-2-propyl-1*H*-imidazol-3-ium bis(trifluoromethylsulfonyl)amide [117]

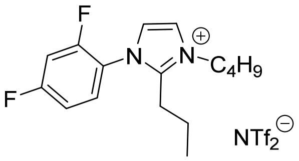

Following general procedure with bromide salt **69** (3.59 g, 10 mmol) and $LiNTf_2$ (4.51 g, 11 mmol, 70 % aq.) to yield **117** (5.5 g, 9.8 mmol, 98 %) as a brown liquid.

^{1}H NMR (300 MHz, $CDCl_3$) δ 7.78 – 7.63 (m, 1H), 7.49 (d, *J* = 2.2 Hz, 1H), 7.29 (d, *J* = 2.2 Hz, 1H), 7.21 – 7.03 (m, 2H), 4.17 (t, *J* = 8.0 Hz, 2H), 2.82 (t, *J* = 8.0 Hz, 2H), 1.99 – 1.79 (m, 2H), 1.63 – 1.30 (m, 4H), 0.99 (t, *J* = 7.3 Hz, 3H), 0.87 (t, *J* = 7.4 Hz, 3H).

^{13}C NMR (75 MHz, $CDCl_3$) δ 164.42 (dd, *J* = 256.9, 11.0 Hz), 157.02 (dd, *J* = 256.4, 12.8 Hz), 148.33, 130.61 (d, *J* = 10.5 Hz), 123.64, 122.13, 119.89 (q, *J* = 321.4 Hz), 118.41 (dd, *J* = 12.7, 4.3 Hz), 113.78 (dd, *J* = 22.9, 3.9 Hz), 106.00 (dd, *J* = 26.9, 22.5 Hz), 49.10, 31.70, 25.55, 20.57, 19.71, 13.62, 13.44.

1-(2,4-difluorophenyl)-3-hexyl-2-propyl-1*H*-imidazol-3-ium bis(trifluoromethylsulfonyl)amide [118]

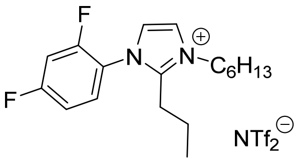

Following general procedure with bromide salt **70** (3.87 g, 10 mmol) and $LiNTf_2$ (4.51 g, 11 mmol, 70 % aq.) to yield **118** (5.7 g, 9.7 mmol, 97 %) as a brown liquid.

^{1}H NMR (300 MHz, $CDCl_3$) δ 7.73 (td, *J* = 8.7, 5.5 Hz, 1H), 7.48 (d, *J* = 2.2 Hz, 1H), 7.29 (d, *J* = 2.2 Hz, 1H), 7.22 – 7.04 (m, 2H), 4.17 (t, *J* = 7.8 Hz, 2H), 2.82 (t, *J* = 8.0 Hz, 2H), 1.92 (quint, *J* = 7.5 Hz, 2H), 1.59 – 1.25 (m, 8H), 0.99 – 0.81 (m, 6H).

^{13}C NMR (75 MHz, $CDCl_3$) δ 164.4 (dd, *J* = 257.1, 11.2 Hz), 157.0 (dd, *J* = 256.3, 12.8 Hz), 148.3, 130.7 (d, *J* = 10.5 Hz), 123.7, 122.1, 119.9 (q, *J* = 321.4 Hz), 118.4 (dd, *J* = 12.7, 4.3 Hz), 113.9 (dd, *J* = 22.9, 3.9 Hz), 106.0 (dd, *J* = 26.9, 22.5 Hz), 49.4, 31.1, 29.8, 26.1, 25.6, 22.4, 20.6, 13.9, 13.7.

1-(2,4-difluorophenyl)-3-octyl-2-propyl-1*H*-imidazol-3-ium bis(trifluoromethylsulfonyl)amide [119]

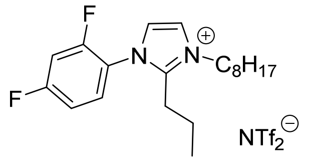

Following general procedure with bromide salt **71** (4.15 g, 10 mmol) and $LiNTf_2$ (4.51 g, 11 mmol, 70 % aq.) to yield **119** (5.8 g, 9.4 mmol, 94 %) as a brown liquid.

^{1}H NMR (300 MHz, $CDCl_3$) δ 7.74 (td, *J* = 8.6, 5.5 Hz, 1H), 7.47 (d, *J* = 2.2 Hz, 1H), 7.29 (d, *J* = 2.2 Hz, 1H), 7.22 – 7.04 (m, 2H), 4.17 (t, *J* = 7.8 Hz, 2H), 2.82 (t, *J* = 8.0 Hz, 2H), 1.92 (quint, *J* = 7.5 Hz, 2H), 1.60 – 1.20 (m, 12H), 0.94 – 0.75 (m, 6H).

^{13}C NMR (75 MHz, $CDCl_3$) δ 164.4 (dd, *J* = 257.1, 11.1 Hz), 157.0 (dd, *J* = 256.3, 12.7 Hz), 148.3, 130.7 (d, *J* = 10.5 Hz), 123.7, 122.1, 119.9 (q, *J* = 321.4 Hz), 118.4 (dd, *J* = 12.7, 4.3 Hz), 113.9 (dd, *J* = 22.9, 3.9 Hz), 106.0 (dd, *J* = 26.9, 22.6 Hz), 49.4, 31.8, 29.8, 29.1, 29.0, 26.5, 25.6, 22.7, 20.6, 14.1, 13.7.

1-(2,4-difluorophenyl)-2-propyl-3-undecyl-1*H*-imidazol-3-ium bis(trifluoromethylsulfonyl)amide [120]

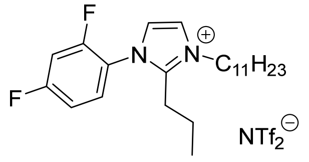

Following general procedure with bromide salt **72** (4.57 g, 10 mmol) and $LiNTf_2$ (4.51 g, 11 mmol, 70 % aq.) to yield **120** (6.31g, 9.6 mmol, 96 %) as a brown liquid.

^{1}H NMR (300 MHz, $CDCl_3$) δ 7.81 – 7.64 (m, 1H), 7.47 (d, *J* = 2.2 Hz, 1H), 7.29 (d, *J* = 2.2 Hz, 1H), 7.19 – 7.06 (m, 2H), 4.16 (t, *J* = 7.7 Hz, 2H), 2.82 (t, *J* = 7.9 Hz, 2H), 1.92 (quint, *J* = 7.4 Hz, 2H), 1.59 – 1.20 (m, 18H), 0.93 – 0.80 (m, 6H).

^{13}C NMR (75 MHz, $CDCl_3$) δ 164.4 (dd, *J* = 257.1, 11.1 Hz), 157.0 (dd, *J* = 256.4, 12.7 Hz), 148.3, 130.7 (d, *J* = 10.1 Hz), 123.7, 122.1, 119.9 (q, *J* = 321.4 Hz), 118.4 (dd, *J* = 12.7, 4.3 Hz), 113.8 (dd, *J* = 23.0, 3.7 Hz), 106.0 (dd, *J* = 26.9, 22.5 Hz), 49.6, 32.0, 29.8, 29.6, 29.5, 29.4, 29.4, 29.1, 26.5, 25.6, 22.8, 20.6, 14.2, 13.7.

6. Electrochemical measurements

Linear sweep voltammetry (LSV) was performed in pure IL using a BioLogic SP-150 potentiostat with a glassy carbon working electrode (diameter 3mm), a Pt-wire counter electrode and an Ag-wire as pseudo reference electrode. The measurement started at 0 V with separate measurements for the anodic and cathodic potential with a sweep rate of 50 mV/s. The electrochemical window was determined with a cut-off current density of 0.1 mA/cm^2.

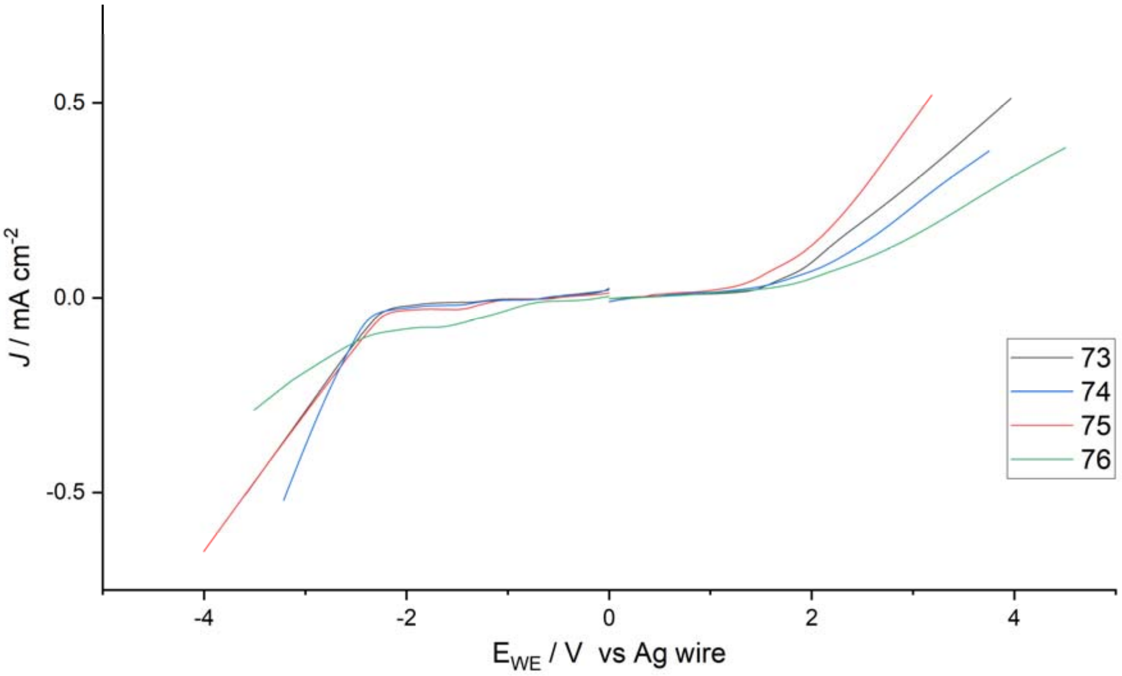

Figure S1. Linear sweep voltammetry of compounds **73-76**.

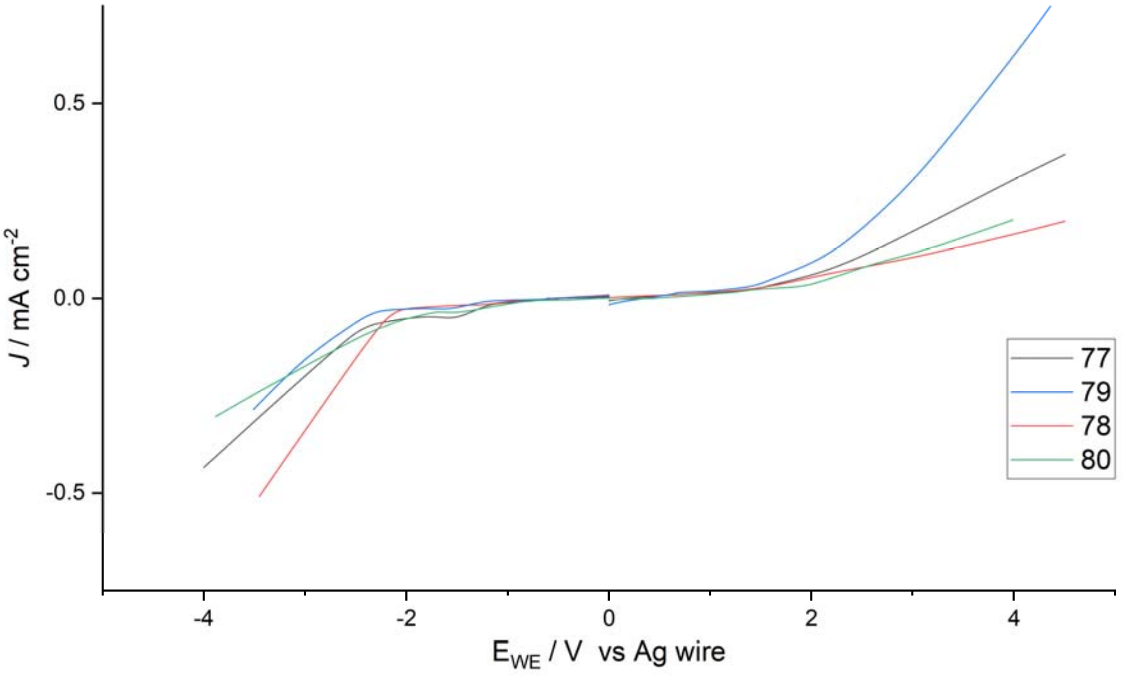

Figure S2. Linear sweep voltammetry of compounds **77-80**.

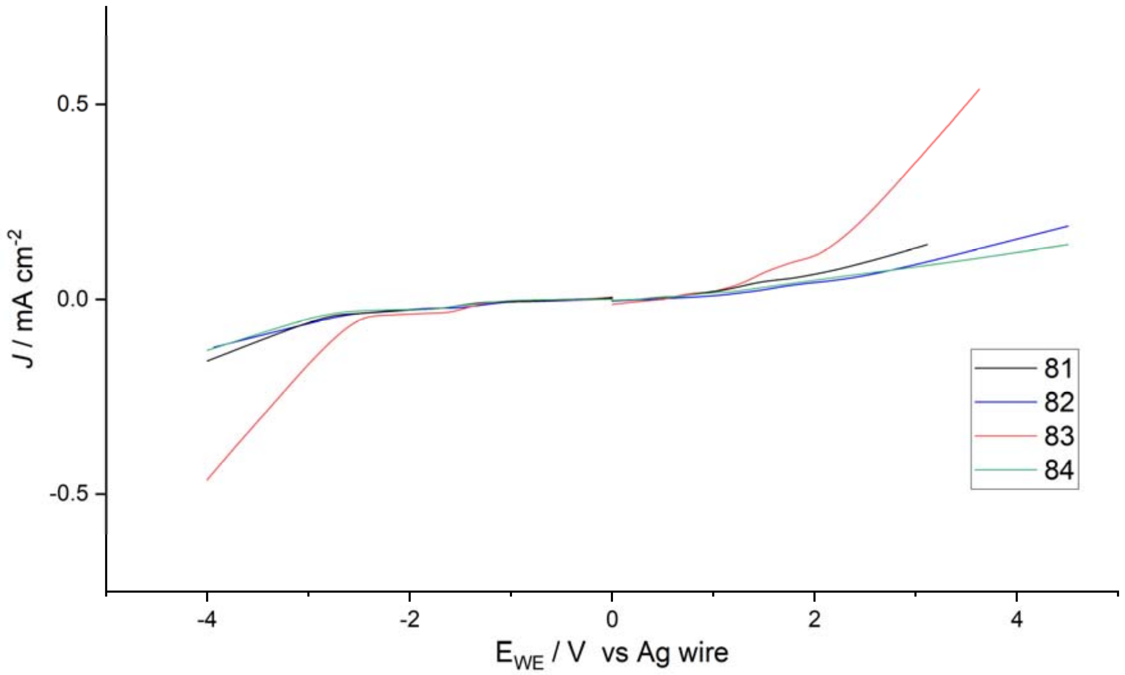

Figure S3. Linear sweep voltammetry of compounds **81-84**.

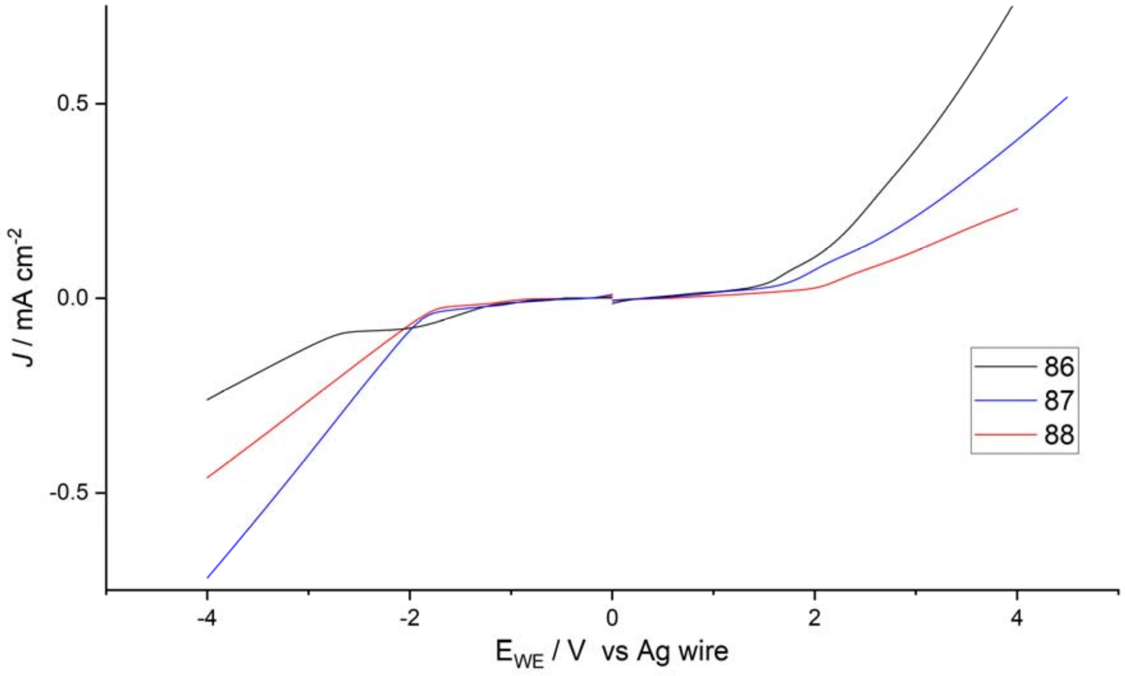

Figure S4. Linear sweep voltammetry of compounds **86-88**.

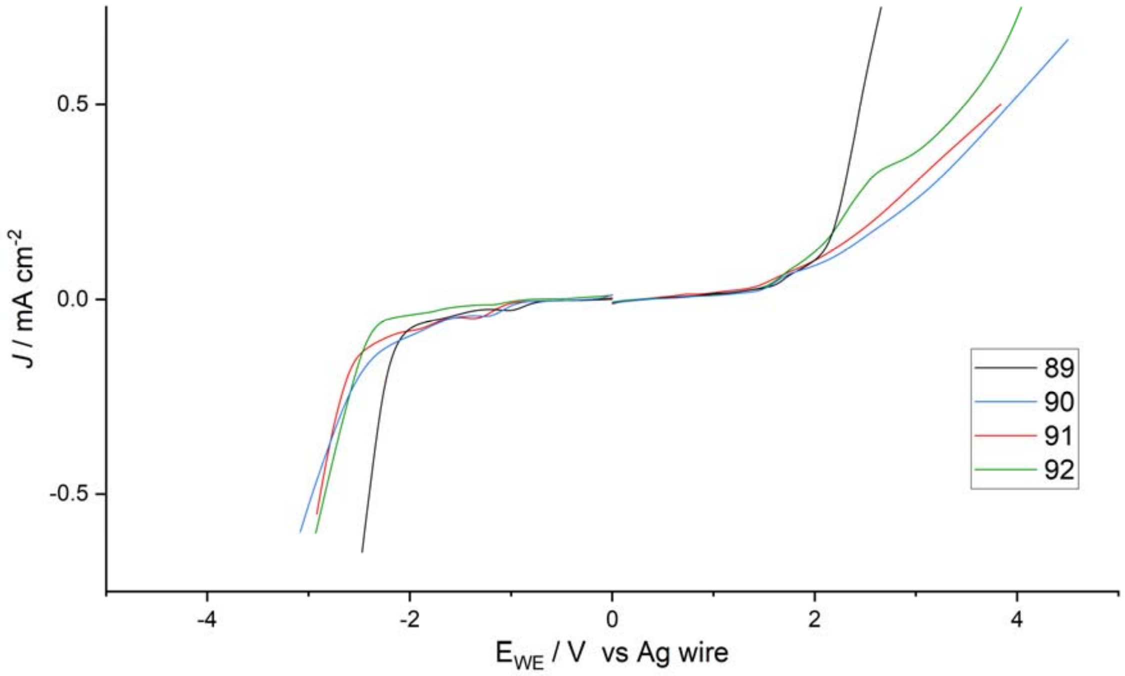

Figure S5. Linear sweep voltammetry of compounds **89-92**.

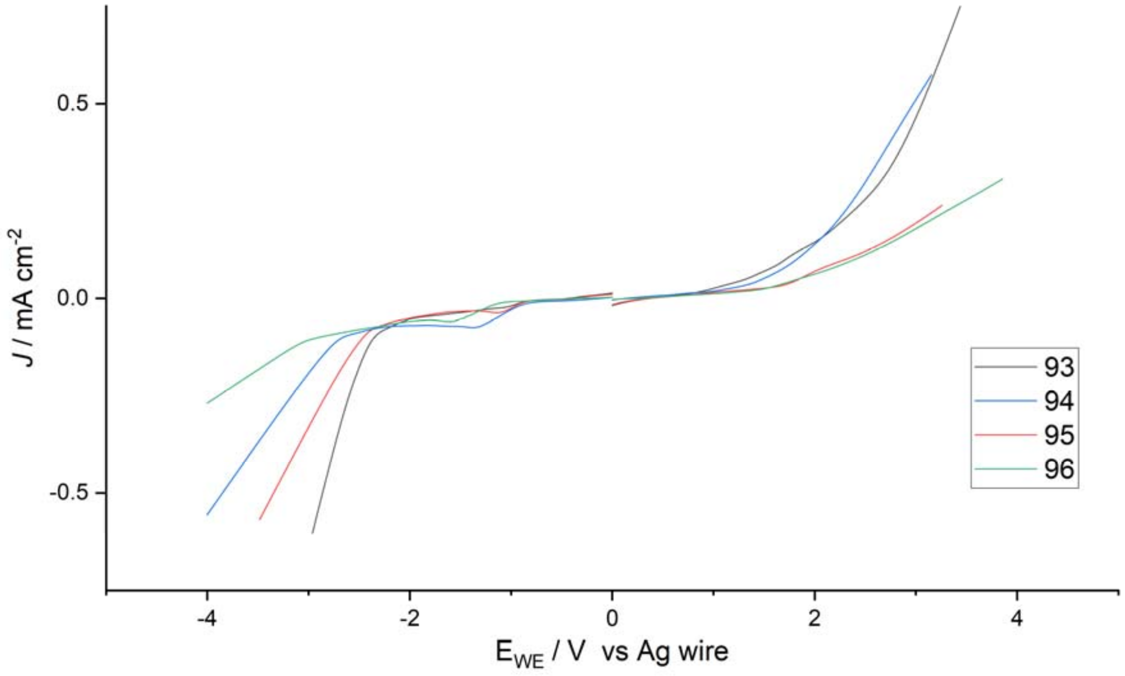

Figure S6. Linear sweep voltammetry of compounds **93-96**.

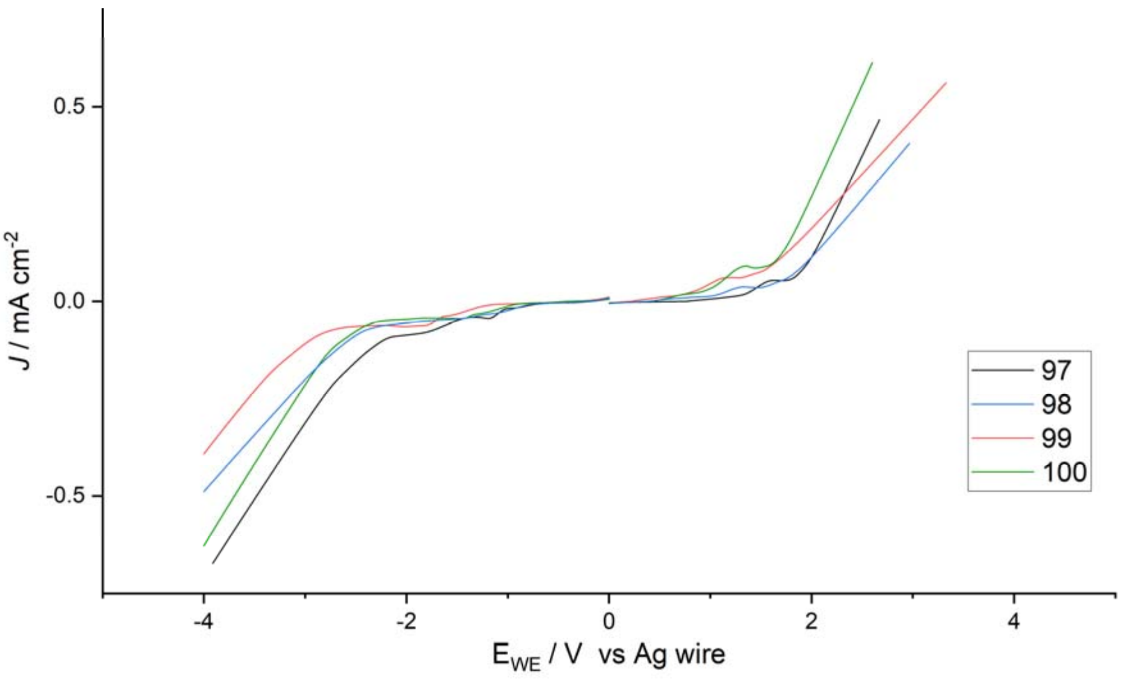

Figure S7. Linear sweep voltammetry of compounds **97-100**.

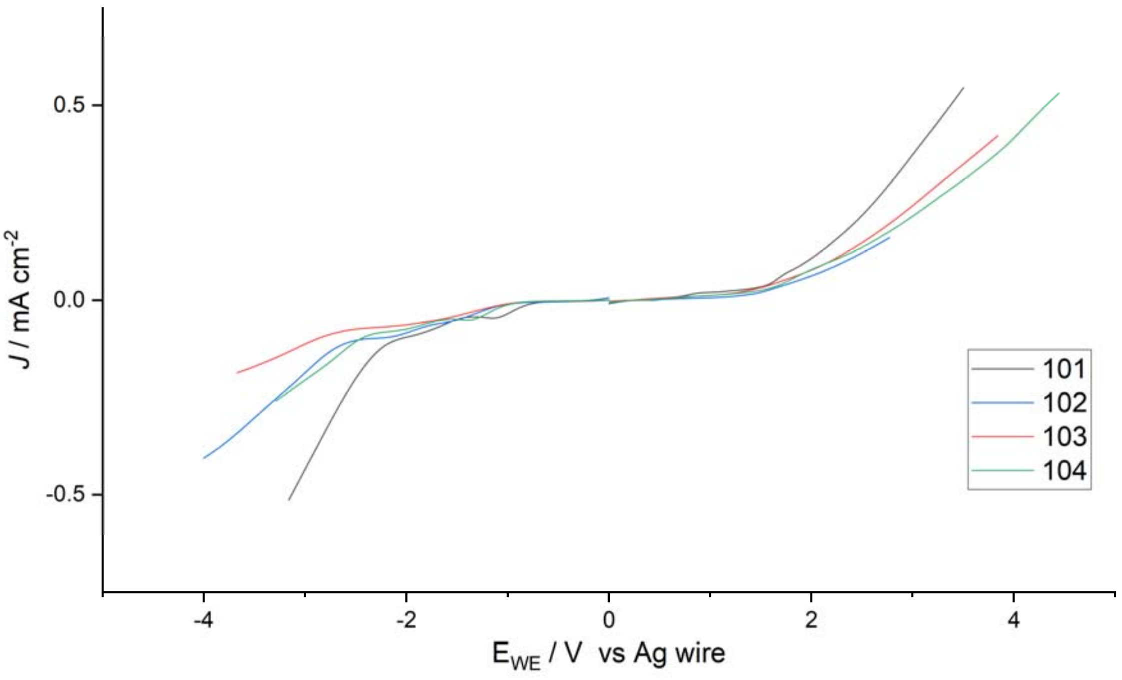

Figure S8. Linear sweep voltammetry of compounds **101-104**.

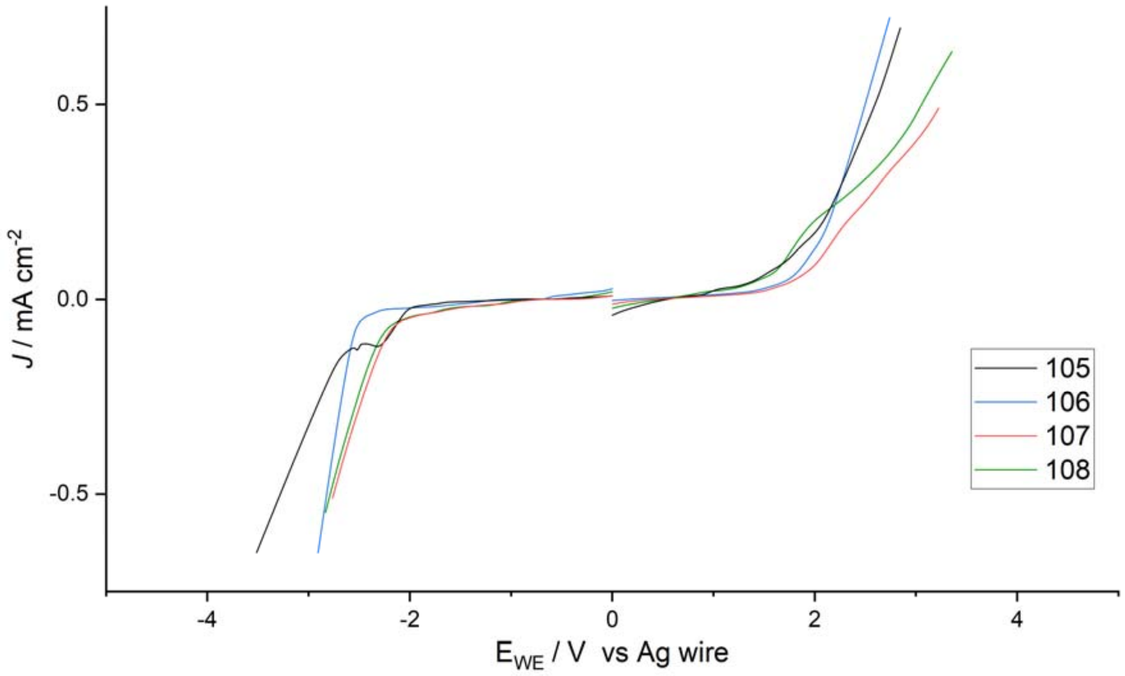

Figure S9. Linear sweep voltammetry of compounds **105-108**.

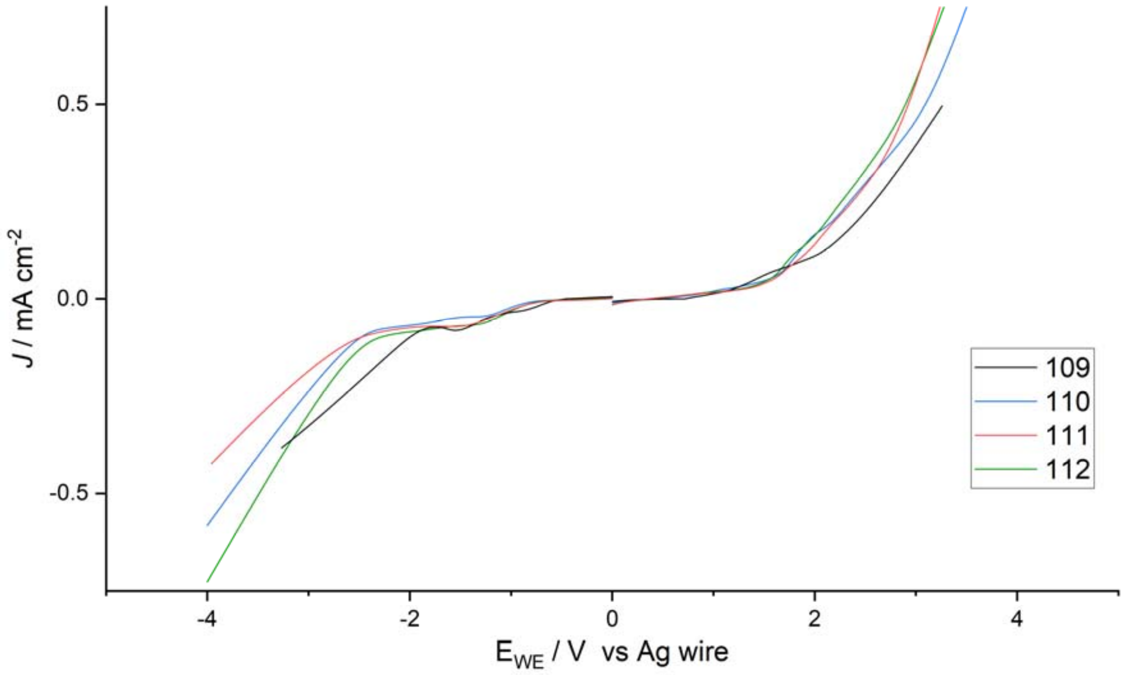

Figure S10. Linear sweep voltammetry of compounds **109-112**.

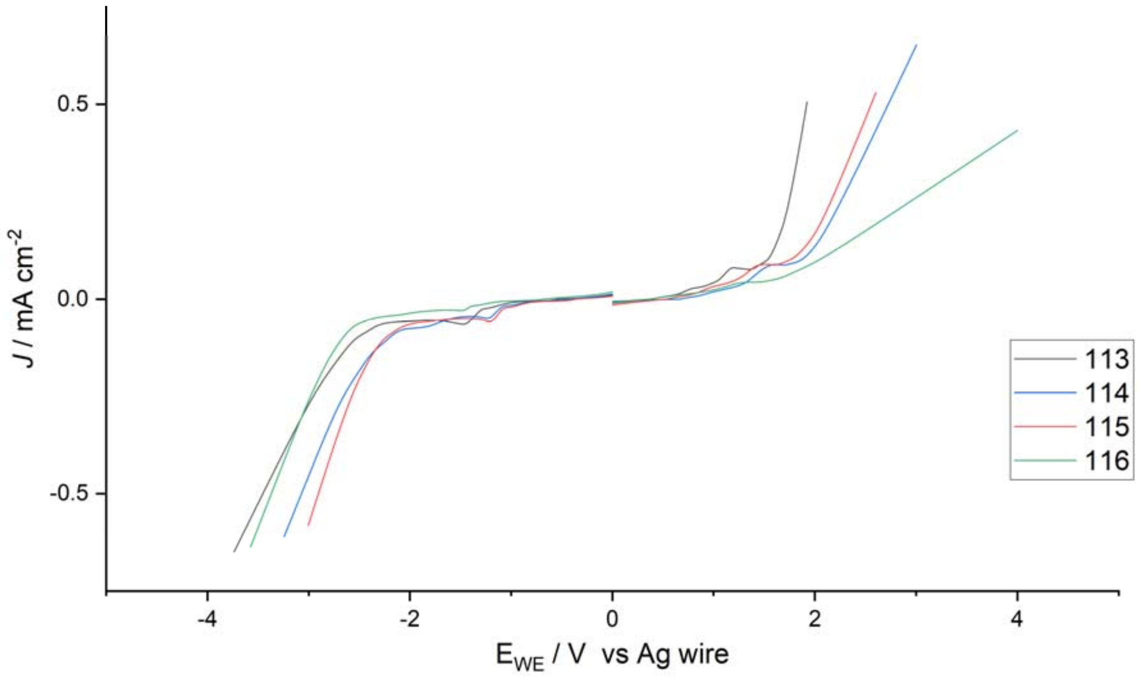

Figure S11. Linear sweep voltammetry of compounds **113-116**.

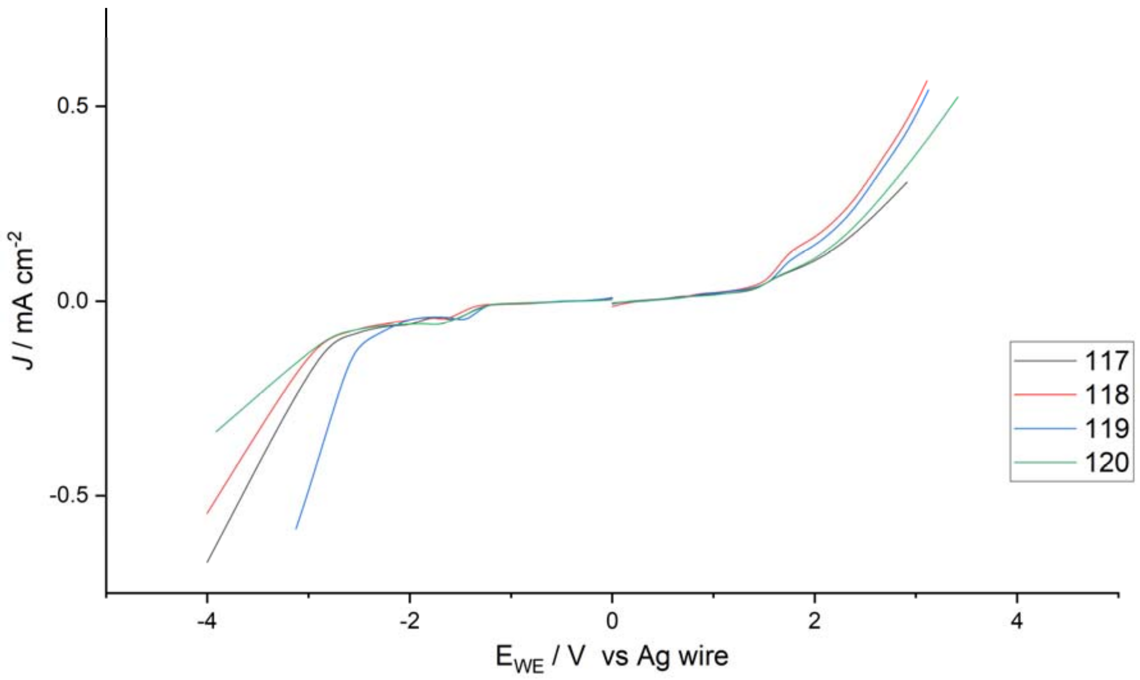

Figure S12. Linear sweep voltammetry of compounds **117-120**.

7. Viscosity measurements

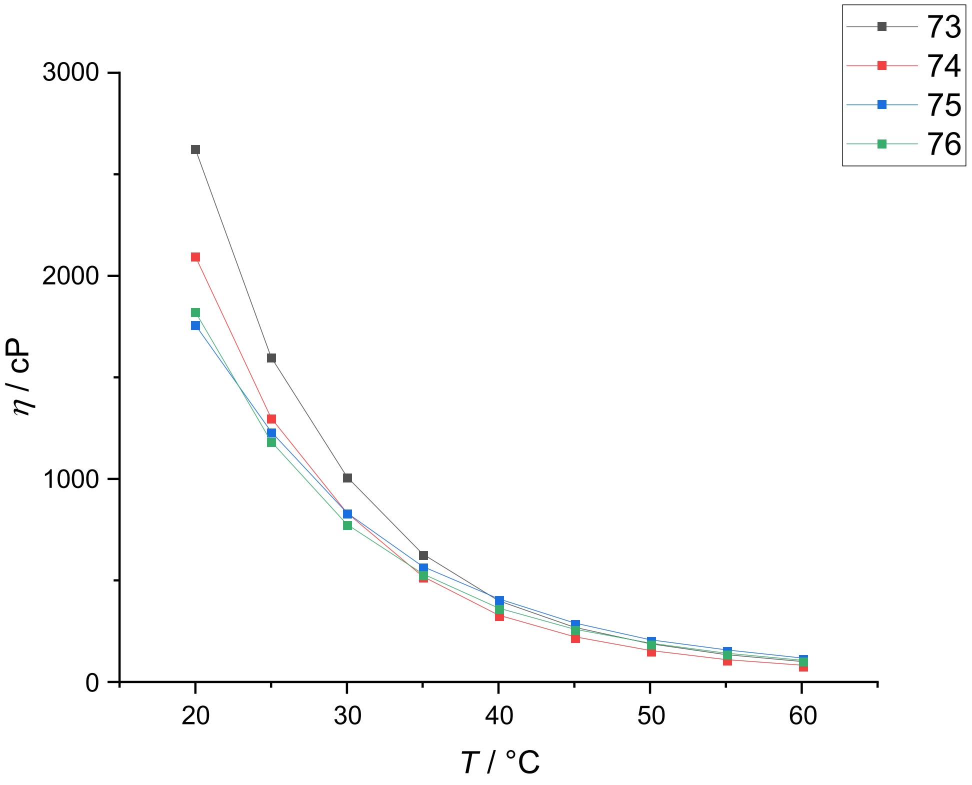

Figure S13. Viscosity measurements of compounds **73-76**.

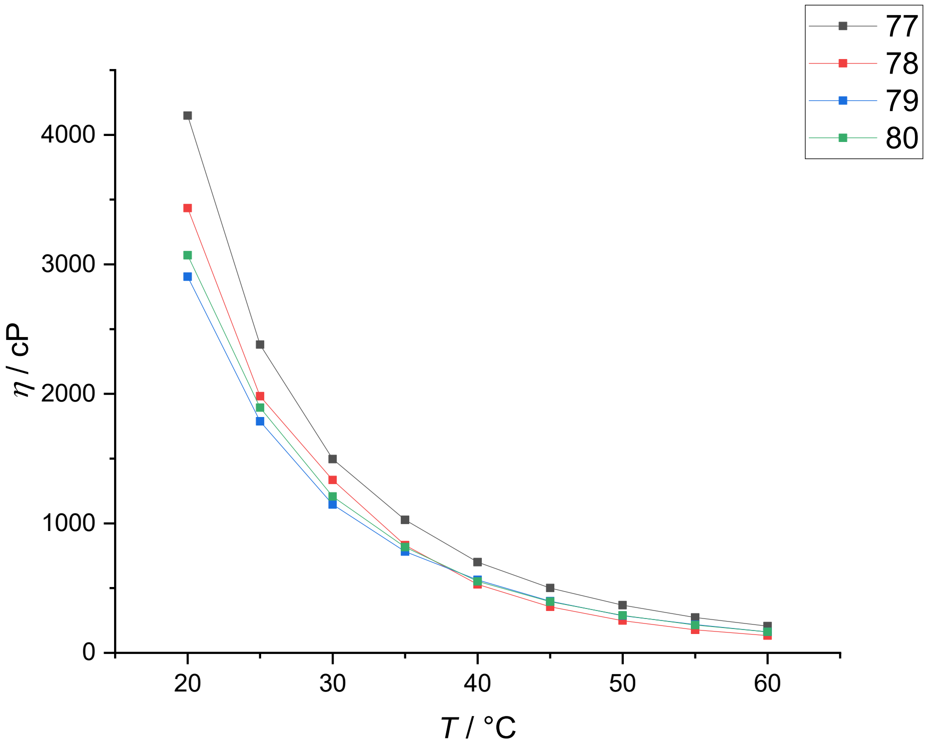

Figure S14. Viscosity measurements of compounds **77-80**.

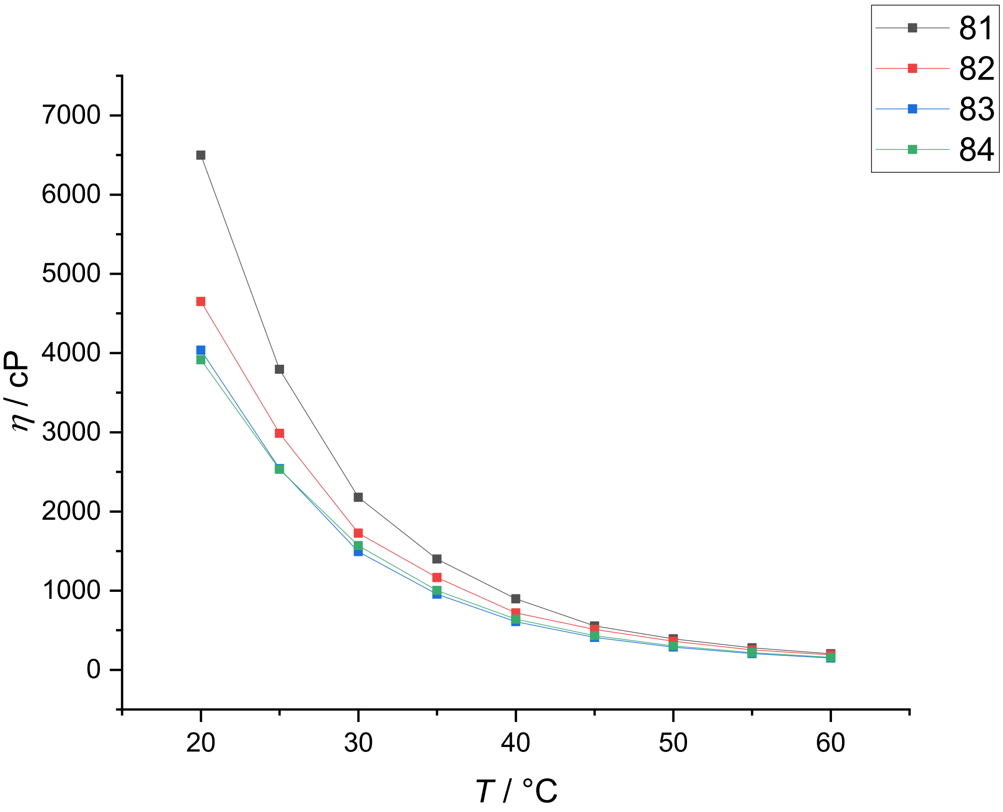

Figure S15. Viscosity measurements of compounds **81-84**.

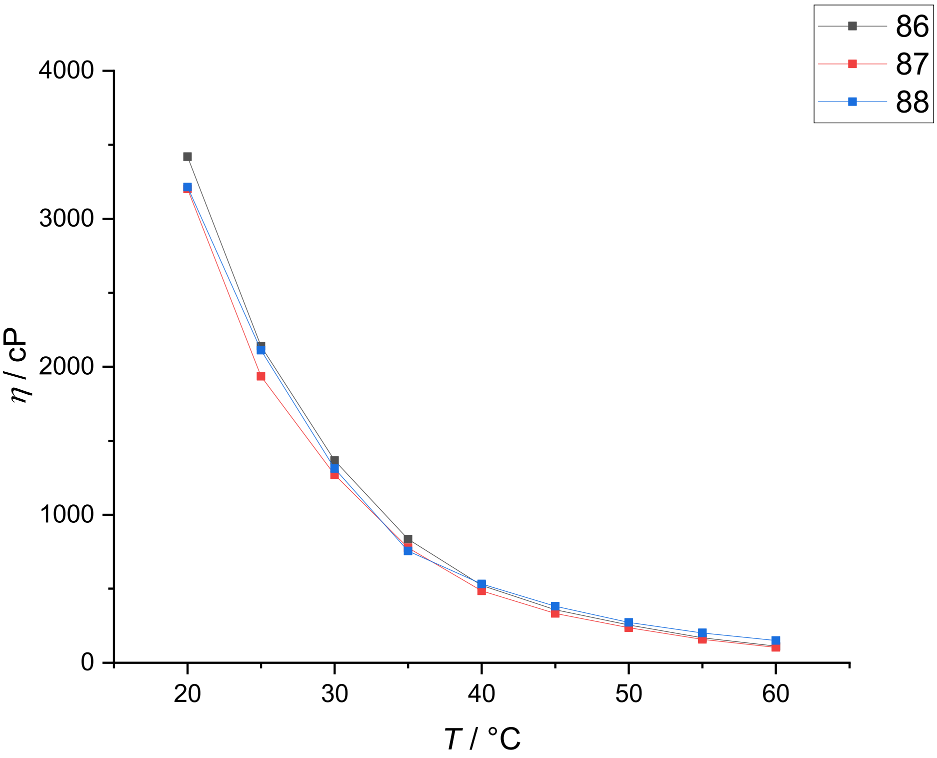

Figure S16. Viscosity measurements of compounds **86-88**.

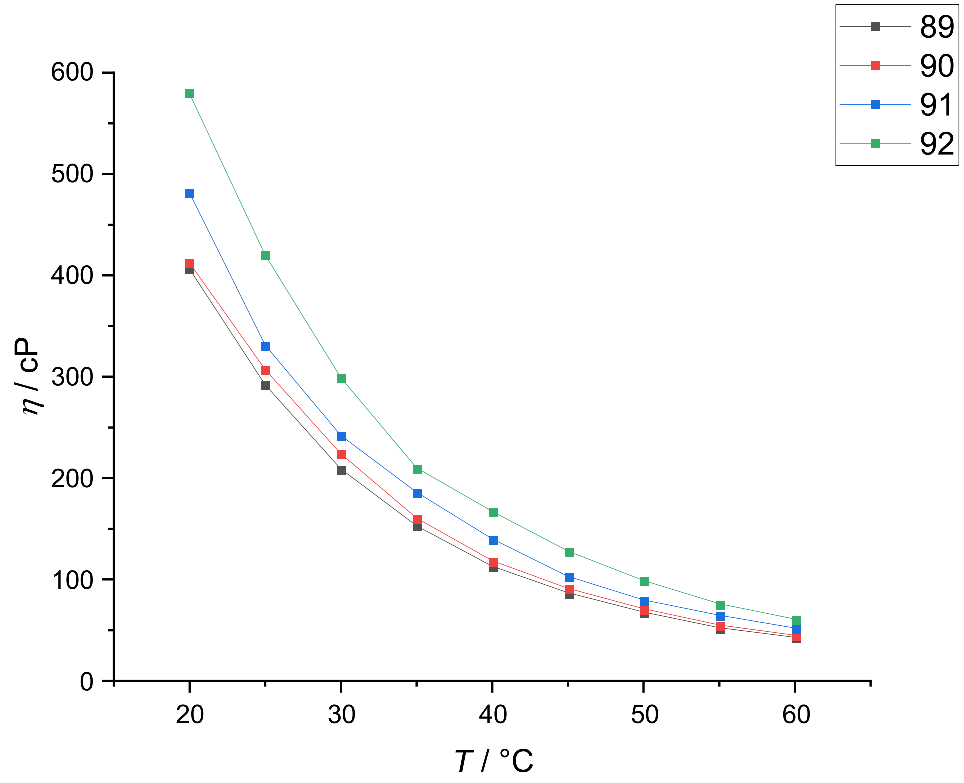

Figure S17. Viscosity measurements of compounds **89-92**.

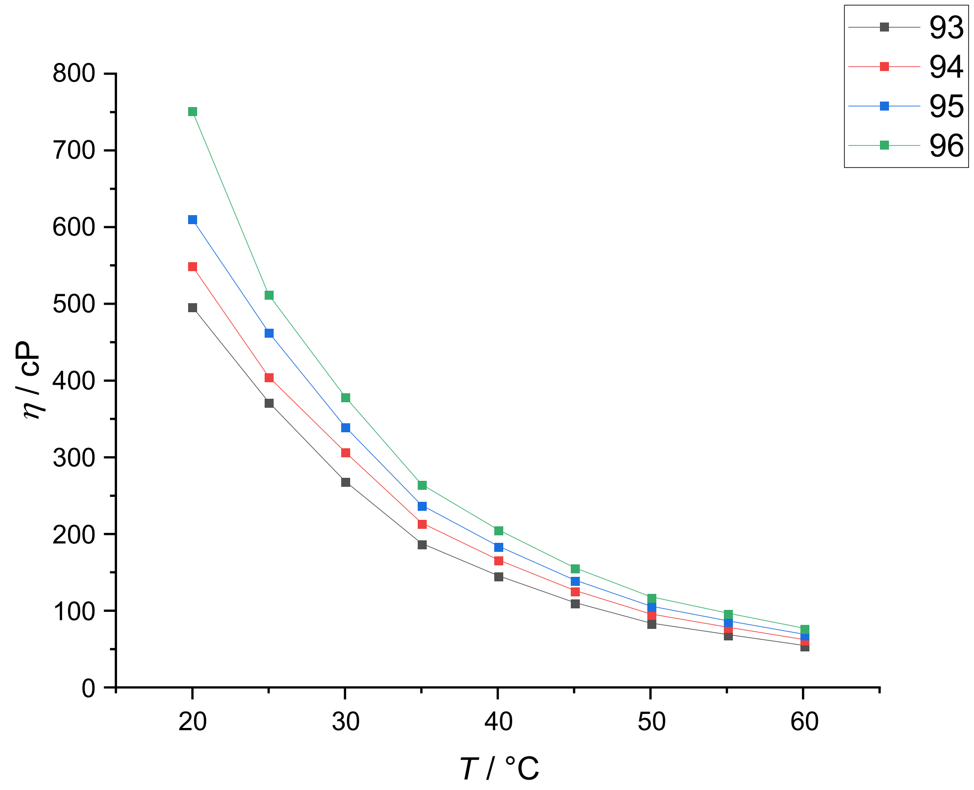

Figure S18. Viscosity measurements of compounds **93-96**.

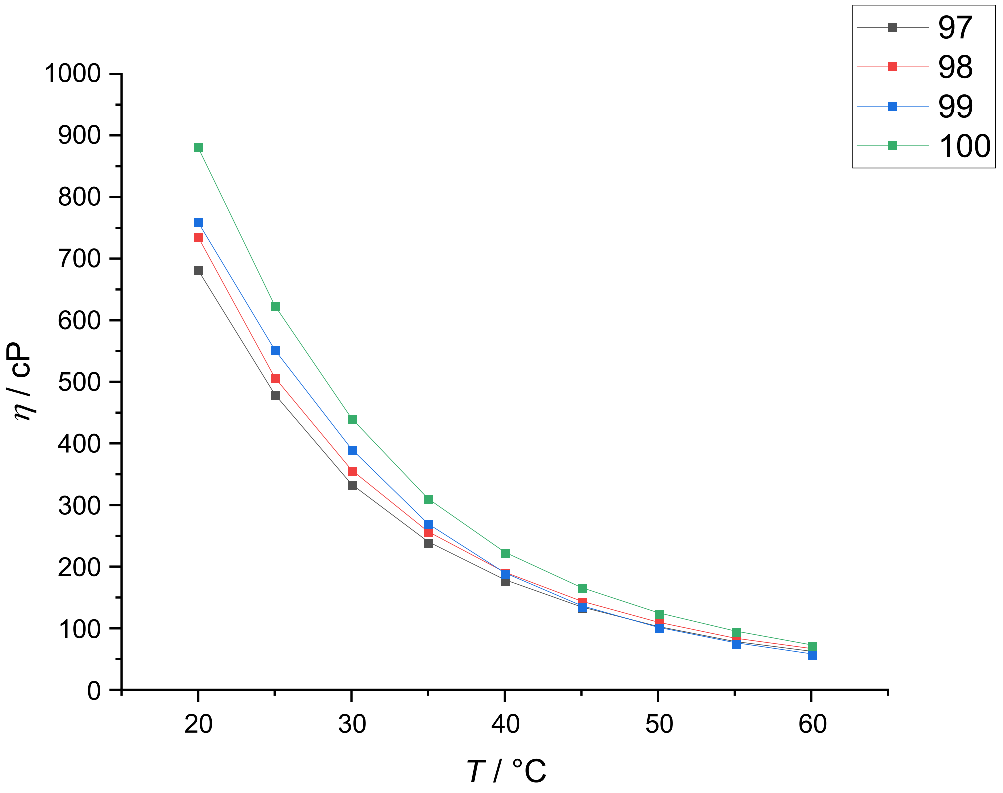

Figure S19. Viscosity measurements of compounds **97-100**.

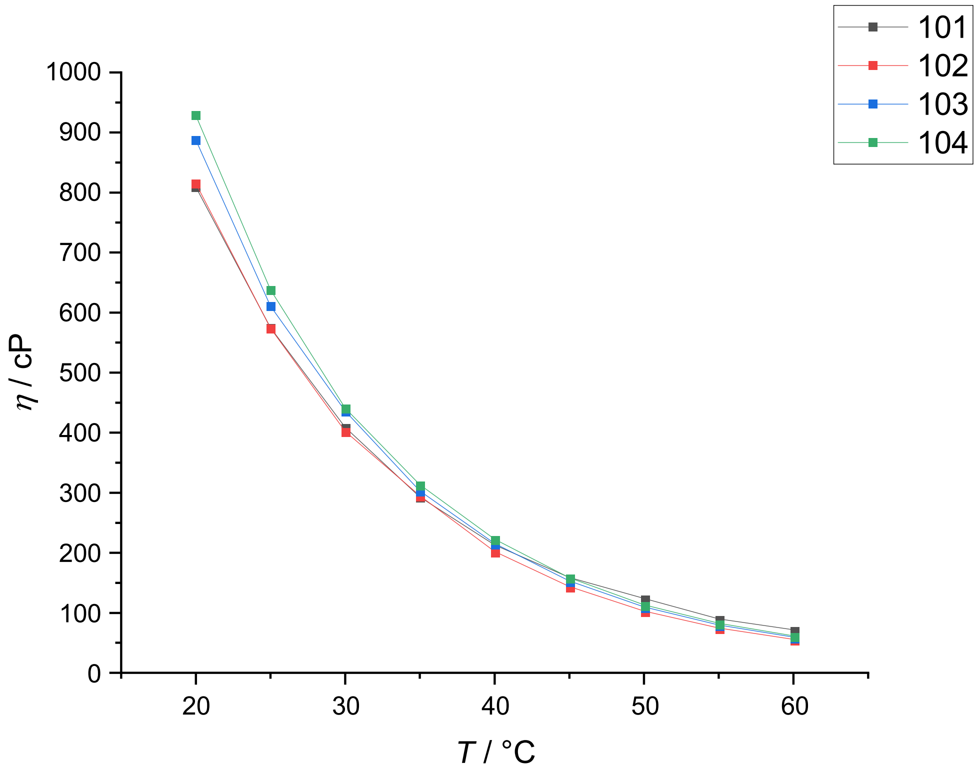

Figure S20. Viscosity measurements of compounds **101-104**.

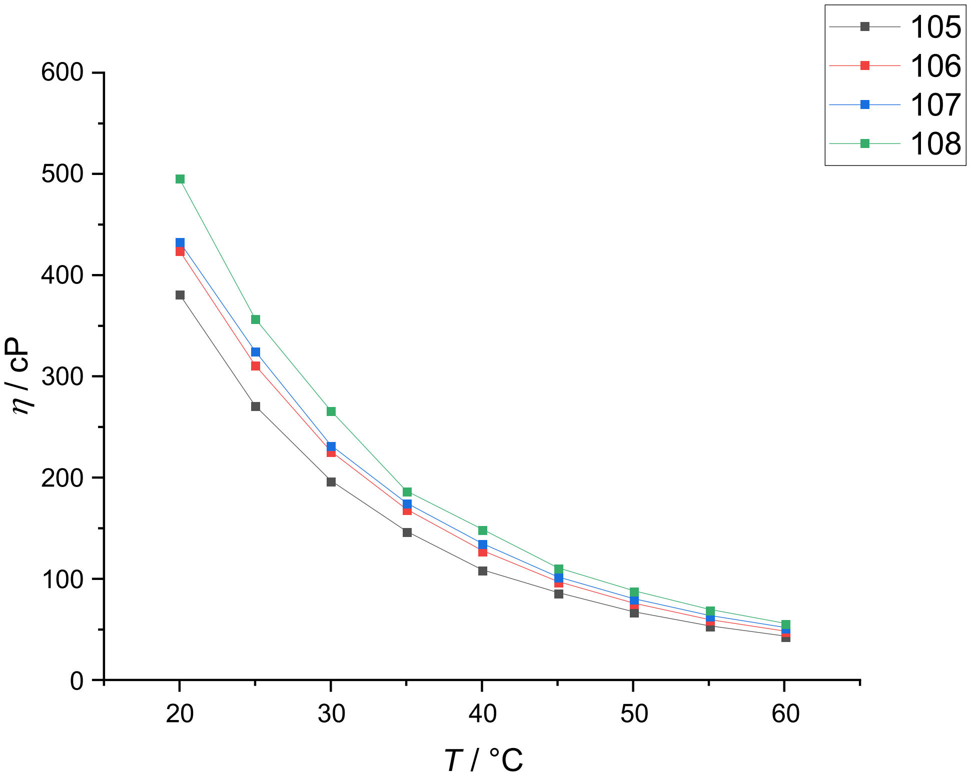

Figure S21. Viscosity measurements of compounds **105-108**.

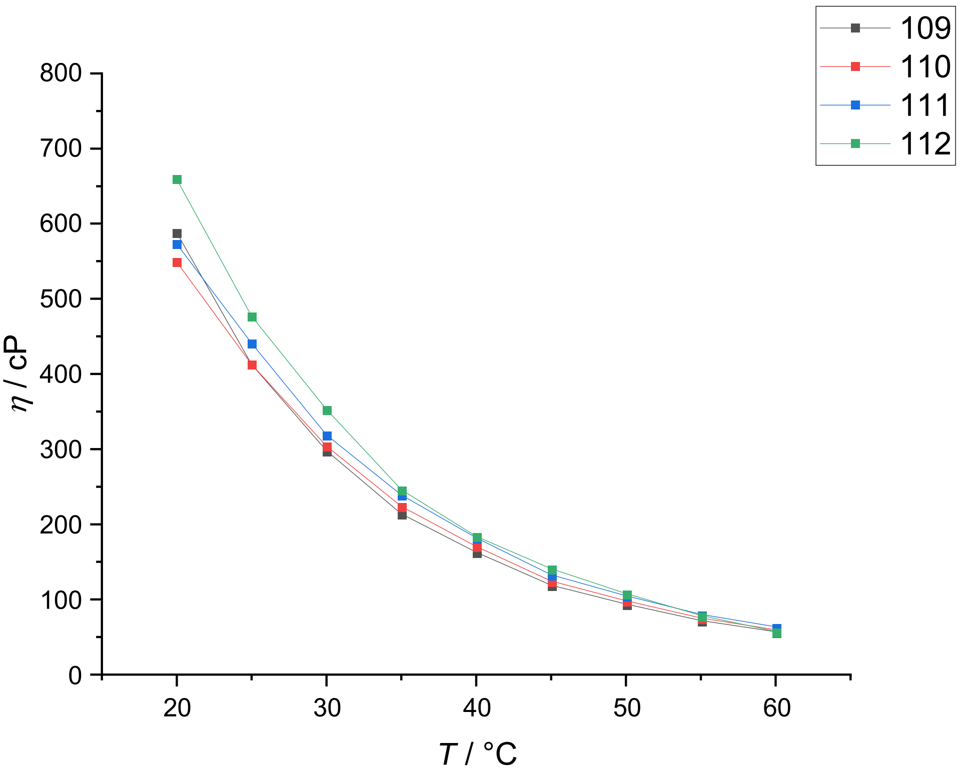

Figure S22. Viscosity measurements of compounds **109-112**.

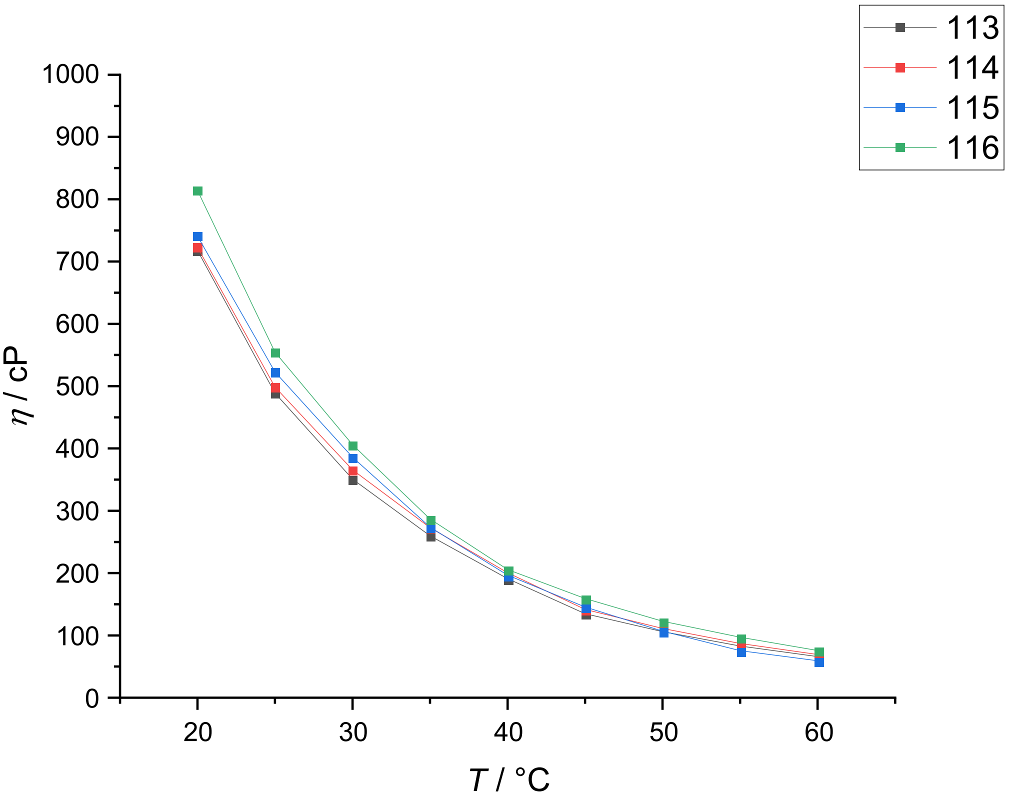

Figure S23. Viscosity measurements of compounds **113-116**.

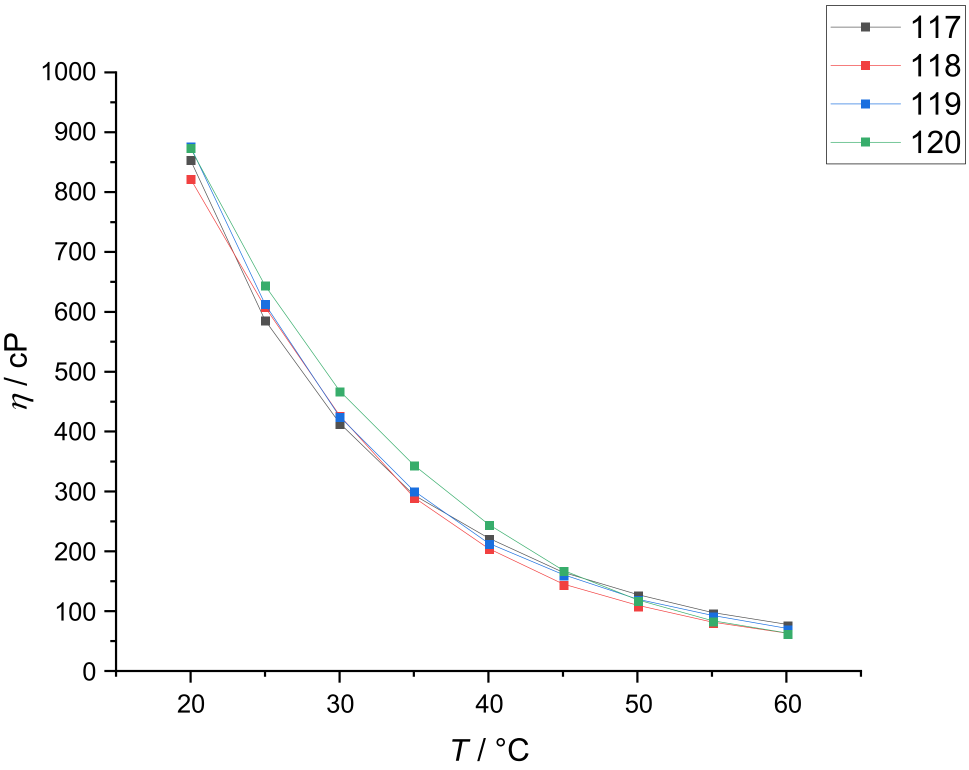

Figure S24. Viscosity measurements of compounds **117-120**.
